# The Anatomy and Phylogenetic Relationships of *“Pelorosaurus“ becklesii* (Neosauropoda, Macronaria) from the Early Cretaceous of England

**DOI:** 10.1371/journal.pone.0125819

**Published:** 2015-06-03

**Authors:** Paul Upchurch, Philip D. Mannion, Michael P. Taylor

**Affiliations:** 1 Department of Earth Sciences, University College London, London, United Kingdom; 2 Department of Earth Science and Engineering, Imperial College London, London, United Kingdom; 3 School of Earth Sciences, University of Bristol, Bristol, United Kingdom; State Natural History Museum, GERMANY

## Abstract

The sauropod dinosaur *“Pelorosaurus” becklesii* was named in 1852 on the basis of an associated left humerus, ulna, radius and skin impression from the Early Cretaceous (Berriasian-Valanginian) Hastings Beds Group, near Hastings, East Sussex, southeast England, United Kingdom. The taxonomy and nomenclature of this specimen have a complex history, but most recent workers have agreed that *“P*.*” becklesii* represents a distinct somphospondylan (or at least a titanosauriform) and is potentially the earliest titanosaur body fossil from Europe or even globally. The Hastings specimen is distinct from the approximately contemporaneous *Pelorosaurus conybeari* from Tilgate Forest, West Sussex. *“P*.*” becklesii* can be diagnosed on the basis of five autapomorphies, such as: a prominent anteriorly directed process projecting from the anteromedial corner of the distal humerus; the proximal end of the radius is widest anteroposteriorly along its lateral margin; and the unique combination of a robust ulna and slender radius. The new generic name *Haestasaurus* is therefore erected for *“P*.*” becklesii*. Three revised and six new fore limb characters (e.g. the presence/absence of condyle-like projections on the posterodistal margin of the radius) are discussed and added to three cladistic data sets for Sauropoda. Phylogenetic analysis confirms that *Haestasaurus becklesii* is a macronarian, but different data sets place this species either as a non-titanosauriform macronarian, or within a derived clade of titanosaurs that includes *Malawisaurus* and Saltasauridae. This uncertainty is probably caused by several factors, including the incompleteness of the *Haestasaurus* holotype and rampant homoplasy in fore limb characters. *Haestasaurus* most probably represents a basal macronarian that independently acquired the robust ulna, enlarged olecranon, and other states that have previously been regarded as synapomorphies of clades within Titanosauria. There is growing evidence that basal macronarian taxa survived into the Early Cretaceous of Europe and North America.

## Introduction

Sauropod dinosaurs were globally distributed mega-herbivores that dominated many Mesozoic terrestrial ecosystems [1–3]. Several aspects of the evolution of sauropods remain poorly understood, ranging from taxonomic and nomenclatural issues (e.g. [1]), to the causes of large-scale fluctuations in their diversity [2] and the impact of gigantic body size on their growth, physiology and biomechanics [3]. Nevertheless, in recent years, significant progress in our understanding of sauropod evolution has been driven by several factors, including an influx of information on new taxa (e.g., see [4]: fig 4), phylogenetic analysis [1,5–19], and new technology such as CT-scanning and Finite Element Analysis (e.g., [20,21]). These advances provide fresh opportunities to revisit material that was first discovered in the 19th or early 20th centuries, in order to address previously intractable taxonomic, phylogenetic or other problems. The current study represents one such reappraisal, focussed on the Early Cretaceous taxon “*Pelorosaurus*” *becklesii* Mantell 1852 [22]. This taxon is significant regionally and globally for several reasons: it potentially represents the earliest known European titanosaur [6,18]; it provides a glimpse of sauropod evolution during the otherwise poorly represented Berriasian-Valanginian, a period that documents the initial recovery of sauropods from a significant extinction event around the Jurassic-Cretaceous boundary [2,23–25]; and it includes the first dinosaurian skin impression recognised by science [26]. Here, we survey the complex and convoluted taxonomic history of “*Pelorosaurus*” *becklesii*, describe and illustrate the specimen in detail, and identify a suite of character states that diagnose this animal as a new taxon. As a by-product of this investigation, we note a number of new or revised characters in the sauropod fore limb. These characters are discussed and incorporated into phylogenetic analyses in order to assess the relationships of”*Pelorosaurus*” *becklesii*. Finally, we consider the new information on “*P*.” *becklesii* in the wider context of sauropod evolutionary history.

## Institutional Abbreviations

AAOD, Australian Age of Dinosaurs Natural History Museum, Winton, Queensland, Australia; AMNH, American Museum of Natural History, New York, USA; ANS, Academy of Natural Sciences, Philadelphia, USA; CM, Carnegie Museum of Natural History, Pittsburgh, USA; CPSGM, Collections Paléontologiques du Service Géologique du Maroc, Direction de la Géologie, Ministère de l’Énergie et des Mines, Rabat, Morocco; CPT, Museo de la Fundación Conjunto Paleontologico de Teruel-Dinopolis, Spain; DMNH, Denver Museum of Natural History, Denver, USA; FMNH, Field Museum of Natural History, Chicago, USA; GCP, Grupo Cultural Paleontológico, Museo Paleontológico de Elche, Spain; HBV, Shijiazhuang University Museum, Shijiazhuang, People’s Republic of China; HMNS, Houston Museum of Natural Science, Houston, USA; I.G., Museo Provincial de Teruel, Spain; IVPP, Institute for Vertebrate Palaeontology and Palaeoanthropology, Beijing, People’s Republic of China; MACN, Museo Argentino de Ciencias Naturales “Bernardino Rivadavia”, Buenos Aires, Argentina; MAL, Malawi Department of Antiquities Collection, Lilongwe and Nguludi, Malawi; MfN, Museum für Naturkunde, Berlin, Germany; MGIGM, Museu Geológico do Instituto Geológico e Mineiro, Lisbon, Portugal; MGUAN, Museu de Geologia da Universidade Agostinho Neto—PaleoAngola Project, Luanda, Angola; ML, Museu da Lourinha, Portugal; MLP, Museo de La Plata, Argentina; MNN, Musée National du Niger, Niamey, Republic of Niger; MPEF, Museo Paleontológico Egidio Feruglio, Trelew, Argentina; MWC, Museum of Western Colorado, Fruita, USA; NHMUK, The Natural History Museum, London, UK; NMB, Staatlisches Naturhistorisches Museum, Braunschweig, Germany; NSMT, National Science Museum, Tokyo, Japan; PIN, Russian Academy of Sciences, Moscow, Russia; PVL, Fundacion Miguel Lillo, Universidad Nacional de Tucumán, San Miguel de Tucuman, Argentina; QM, Queensland Museum, Brisbane, Australia; UNPSJB, Universidad Nacional de la Patagonia San Juan Bosco, Comodoro Rivadavia, Argentina; USNM, United States National Museum of Natural History, The Smithsonian Institute, Washington D.C., USA; WDC, Wyoming Dinosaur Center, Thermopolis, USA: YPM, Yale Peabody Museum, New Haven, USA; ZDM, Zigong Dinosaur Museum, Zigong, People’s Republic of China; ZPAL, Institute of Paleobiology, Polish Academy of Sciences, Warsaw, Poland (N.B. the holotype of *Opisthocoelicaudia* was held at this institution when it was described in 1977 [27], but it has subsequently been returned to Ulan Bator, Mongolia).

## Historical Background: Discovery and Taxonomy of “*Pelorosaurus*” *becklesii* [22]

The first sauropod specimens to be described scientifically were discovered and studied in the United Kingdom during the early and mid-19^th^ Century [28–30]. This ‘head start’ has had some unfortunate consequences because of the combined deleterious effects of fragmentary material, a tendency for early workers to refer non-overlapping specimens to the same taxon, the absence of opportunities to make comparisons with well-preserved and complete skeletons, over-enthusiastic naming of new taxa, and the phenomenon of historically obsolete diagnostic characters [31]. As a result, the taxonomy and nomenclature of British sauropods is notorious for its complexity and confused nature, and it is only in the past two decades that it has been possible to begin to resolve these problems using carefully evaluated synapomorphies and autapomorphies (e.g. [1,6,17,18,28,32–39]. “*Pelorosaurus*” *becklesii* became thoroughly enmeshed in this taxonomic tangle during the 1880s, and numerous claims concerning its status and affinities have been sporadically proposed since then. Here, therefore, we briefly summarise the history of this species in order to present background information relevant to the revised diagnosis and other conclusions resulting from this reassessment of “*P*.” *becklesii*.

**Fig 1:**
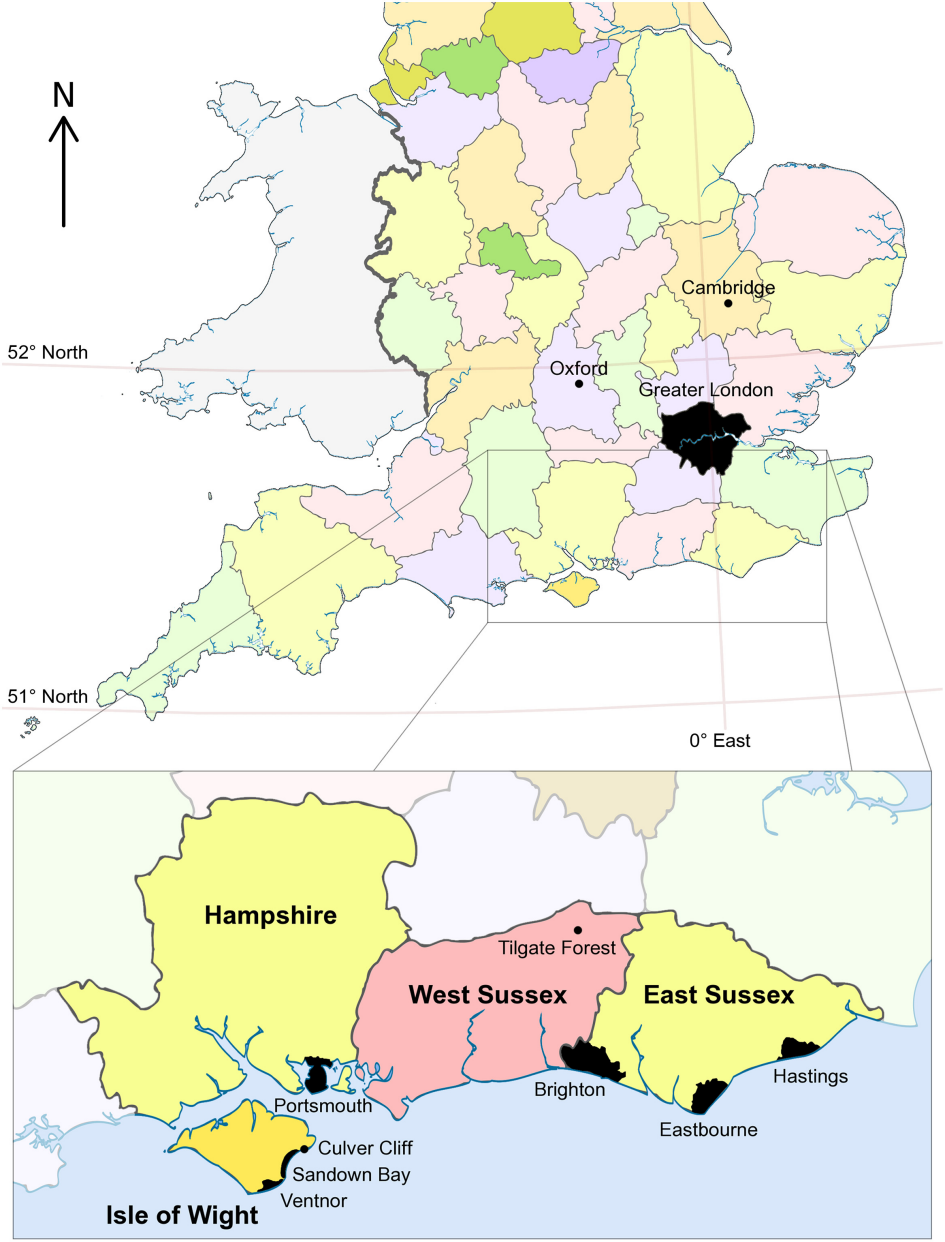
Map showing England and Wales, with boundaries for English counties. The magnified inset shows the Isle of Wight and East and West Sussex in more detail, marking the positions of selected major towns/cities and the fossil localities mentioned in the main text. Based on "English ceremonial counties 1998" by Dr. Greg (see the Wikipedia website at http://en.wikipedia.org/wiki/File:English_ceremonial_counties_1998.svg.: accessed 1^st^ August 2014): CC By-SA 3.0.

In 1841, Richard Owen [40] named *Cetiosaurus* (without a species name) on the basis of fragmentary sauropod remains from various localities in England [28,35]. At this time, and for many years subsequently, Owen maintained that these and other sauropod specimens belonged to gigantic carnivorous marine reptiles, and he later went on to exclude them from Dinosauria [41,42]. Owen [41] named four species of *Cetiosaurus*, one of which was *C*. *brevis* based on several vertebrae and chevrons, such as NHMUK R10390 from Sandown Bay and Culver Cliff, Isle of Wight, and NHMUK R2133, R2115, R2544–2550 from the Hastings Beds in what is now West Sussex (Fig 1). Most of these specimens were re-identified as belonging to *Iguanodon* by Melville [43], except for four anterior caudal vertebrae (NHMUK R2544–2547) and three chevrons (NHMUK R2548–2550) from Tilgate Forest, West Sussex, which he proposed as the type material of a new species of *Cetiosaurus*, *C*. *conybeari* ([43]: p.297). A large right humerus (NHMUK 28626) was discovered in 1850 from ‘…a few yards…’ away from the Tilgate Forest site that had yielded the *C*. *conybeari* tail elements [44]. On this basis, Mantell [44] erected the new generic name *Pelorosaurus* and created the combination of *P*. *conybeari* for the humerus, caudals and chevrons. Mantell also noted the robust and straight morphology of the humerus, and the presence of a medullary cavity in its shaft, and proposed that *Pelorosaurus* therefore represented a large terrestrial dinosaur rather than a marine reptile. Owen [42] disagreed with both Melville and Mantell and therefore retained the name *Cetiosaurus brevis* for NHMUK R2544–2550 and used *Pelorosaurus conybeari* (N.B. misspelt by Owen as ‘*conybearii*’) for the right humerus alone.

In 1852, Samuel H. Beckles recovered a block of ‘Wealden Sandstone’ exposed at low tide on the Sussex Coast ([22]: p.143). This block contained the associated left humerus, ulna and radius (all three numbered NHMUK R1870), as well as a skin impression (NHMUK R1868), from what we now recognise to be a sauropod. The report of this discovery has always previously been referenced as ‘Mantell 1852’ [22], although in fact it is clearly an anonymous account of a talk given by Mantell. There are few descriptive details (and no plates or illustrations), except for some measurements of the limb bones and a mention that the scales of the skin impression are hexagonal. Mantell noted the general similarity of the NHMUK R1870 humerus to that of *Pelorosaurus conybeari*, but also recognised that they represented distinct species because the new humerus was somewhat shorter and more robust than the latter. On this basis, Mantell [22] erected a new species of *Pelorosaurus*, which he named *P*. *becklesii*. Little more was written about the latter taxon during much of the next three decades (i.e., from 1853 to 1888). For example, Owen [42] completely ignored “*P*.” *becklesii*, despite the fact that he paid considerable attention to the *Cetiosaurus brevis* and *Pelorosaurus conybeari* specimens from Tilgate Forest. This long neglect might reflect the fact that the “*P*.” *becklesii* material remained in the private Beckles collection until it was purchased by The Natural History Museum, London, in 1891 [45].

Significant advances in the understanding of sauropod dinosaurs were made as a result of discoveries in the 1870s and 1880s. In particular, more complete material of *Cetiosaurus* from Oxfordshire was described by Phillips in 1871 [46], enabling him to recognise that this animal was a gigantic terrestrial herbivorous dinosaur. This view was reinforced by the numerous new discoveries made in the Western USA by O. C. Marsh, E. D. Cope and associated colleagues, resulting in key advances such as: the recognition of the group Sauropoda [47]; the naming of iconic taxa, including *Camarasaurus* [48] and *Apatosaurus* [49]; description of the first well-preserved skulls and cervical series (e.g., [50]); and the first publication of a skeletal reconstruction of a sauropod [51]. A series of papers by R. Lydekker and others during the late 1880s and 1890s aimed to revise and clarify the taxonomy, nomenclature and relationships of British sauropods in the light of these new discoveries. Unfortunately, these studies tended to have the opposite effect, at least when viewed with the benefits of hindsight and modern taxonomic and phylogenetic practices. Lydekker ([52]: p.55) mentioned the humerus of *Pelorosaurus conybeari* (N.B. he cited the specimen number ‘28266’, but this should be ‘28626’) and suggested that it was potentially synonymous with *Ornithopsis*. In the same paper (p.58) Lydekker discussed the material of “*P*.” *becklesii* as follows:

‘I have already mentioned *Cetiosaurus brevis* under the head of *Pelorosaurus*, but I may here bring to notice an associated humerus, radius, and ulna, from the Wealden of the Isle of Wight, in the collection of Mr. S. H. Beckles, of which the British Museum possesses casts (no. 28701). The length of the humerus is 0.620 (24.5 inches); its shaft is much shorter than that of the corresponding bone of *C*. [*Cetiosaurus*] *oxoniensis*; but it approximates to that piece in its widely expanded head, and there appears a probability that these bones may belong *to C*. [*Cetiosaurus*] *brevis*, in which case that form would differ widely from the type species, and would likewise be certainly distinct from *Pelorosaurus*. On the other hand, these limb-bones may perhaps be referable to *Titanosaurus*, or possibly even to a new genus.’

Lydekker was clearly referring to “*P*.” *becklesii*, as demonstrated by the facts that, at the time, this was the only British sauropod from the Wealden that had produced an associated humerus, ulna and radius, that the original material was in the Beckles collection, and that the stated length of the humerus agrees closely with that of NHMUK R1870 (Table 1). The claim that these specimens came from the Isle of Wight, rather than East Sussex, is therefore an error.

**Table 1:** Measurements of the fore limb elements of *Haestasaurus*.

Element	Dimension	Measurement (in mm)
Humerus	Length	599
	Maximum transverse width of proximal end	268
	Maximum anteroposterior width of proximal end	133
	Distance from proximal end to most prominent point of deltopectoral crest	212
	Distance from proximal end to point where deltopectoral crest disappears	260
	Transverse width of midshaft	113
	Anteroposterior width of midshaft	78
	Circumference of midshaft	307
	Maximum transverse width of distal end	211
	Maximum anteroposterior width of distal end	130
Ulna	Length	421
	Length of anteromedial process of the proximal end	146
	Length of the anterolateral process of the proximal end	113
	Transverse width at midshaft	63
	Anteroposterior width at midshaft	50
	Circumference at midshaft	178
	Maximum transverse width of distal end	68
	Maximum anteroposterior width of distal end	106
Radius	Length	404
	Maximum transverse width of proximal end	100+
	Maximum anteroposterior width of proximal end	88
	Transverse width at midshaft	61
	Anteroposterior width at midshaft	42
	Circumference at midshaft	167
	Maximum transverse width of distal end	113
	Maximum anteroposterior width of distal end	74

Measurements of the fore limb elements of *Haestasaurus becklesii* (NHMUK R1870). N.B. ‘midshaft’ refers to the point on each element, at approximately midlength, where the shaft is most slender.

Marsh [53] erected the genus “*Morosaurus*” for sauropod remains from the Late Jurassic Morrison Formation of North America, but this has subsequently been shown to be a junior synonym of *Camarasaurus* [54]. When Marsh visited England in 1888, he compared British dinosaur material with that from North America and published some of his conclusions in 1889 [55]. Marsh ([55]: p.325) suggested that “*P*.” *becklesii* was referable to “*Morosaurus*” based on overall similarity and limb proportions, and created the new combination ‘*Morosaurus*” *becklesii*. Nicholson and Lydekker [56] and Lydekker [57,58] synthesized the original views of Melville and Mantell with the more recent proposal of Marsh, recognising that “*Cetiosaurus brevis*”, the caudals of *Pelorosaurus conybeari*, and ‘*M*. *becklesii*’ were congeneric and so created the new combination “*M*.” *brevis* (see also [59,60]). “*M*.” *brevis* was assigned to the Cetiosauridae, and its diagnosis was expanded to include dental and vertebral characters based on the referred specimens (e.g. [57]). As noted by several previous authors (e.g. [36,37]), the referral of “*Pelorosaurus*” *becklesii* to ‘*Morosaurus*’ cannot be supported because the latter genus is not a valid taxon and the diagnostic characters cited by Marsh, Lydekker and others are vague and/or known to be widespread among many sauropod species.

Huene ([61]: p122–123) regarded *Pelorosaurus* as synonymous with *Ornithopsis* Seeley 1870 [62], with the former name having priority because it was erected earlier. He did not discuss the different species of *Pelorosaurus*, but listed it as including material from the Kimmeridgian of England and France, and Wealden of England: presumably this distribution reflects the inclusion of Jurassic *Ornithopsis* specimens. Huene placed *Pelorosaurus* in the ‘Subfamily Brachiosauridae’ (sic). A more detailed indication of Huene’s opinion on these issues was presented in 1932 [63]. Huene ([63]: pp.286–288, figs 34, 35) described and illustrated the limb material of “*P*.” *becklesii*. He argued that the caudal vertebrae assigned to *Cetiosaurus brevis* (NHMUK R2544–2547) belonged to a member of the theropod family Megalosauridae: thus he rejected previous claims by Lydekker that these specimens and “*P*.” *becklesii* should be considered to be conspecific. Huene also argued that the humerus of “*P*.” *becklesii* differed from that of *Pelorosaurus conybeari* in both its proportions and the morphology of key features such as the ‘processus lateralis’ (= the deltopectoral crest). He therefore considered “*P*.” *becklesii* to be distinct from *Pelorosaurus conybeari*, and referred to the former as ‘Gen. (?) *becklesii*’ (N.B. not ‘*Camarasaurus becklesii*’ as claimed by McIntosh [64]). Although Huene did not follow Marsh or Lydekker in regarding “*P*.” *becklesii* as referable to “*Morosaurus*”, he does seem to have believed that the two taxa were closely related because he placed ‘Gen. (?) *becklesii*’ and *Camarasaurus* in the Camarasaurinae, within the family Brachiosauridae ([63]: p.251).

“*Pelorosaurus*” *becklesii* then received very little attention during the rest of the 20^th^ Century, apart from occasional passing references or inclusion in lists of sauropod taxa. Swinton ([60]: p.211) mentioned “*P*.” *becklesii* in passing, misspelling the species name as ‘*becklesi*’. He noted the presence of “*Morosaurus brevis*” on the Isle of Wight, but did not suggest that “*P*.” *becklesii* should be considered congeneric or conspecific with this taxon. Romer [65] and Steel [66] accepted the validity of *Pelorosaurus* and regarded it as synonymous with several other poorly known European taxa, including: "*Chondrosteosaurus*" Owen 1876 [67], "*Dinodocus"* Owen 1884 [68], *Eucamerotus* Hulke 1872 [69], “*Gigantosaurus*” Seeley 1869 [70], “*Hoplosaurus*” Lydekker 1890 [57], "*Ischyrosaurus*" Hulke 1874 [71], ‘*Morinosaurus*” Sauvage 1874 [72], “*Neosodon*” Moussaye 1885 [73], *Oplosaurus* Gervais 1852 [74], and *Ornithopsis* Seeley 1870 [62]. All of these referrals made by Lydekker, Romer and Steel have been rejected recently [1,32,36] on the basis that either there is no anatomical overlap between the *Pelorosaurus conybeari* holotype and the referred taxon (e.g., *Oplosaurus armatus* is based on an isolated tooth, NHMUK R964), or because there are no autapomorphies uniting specimens where comparisons can be made. Romer [65] only listed genera, so there is no information on his views concerning *Pelorosaurus conybeari* and “*P*.” *becklesii*. However Steel ([66]: p.68) proposed a detailed revision of sauropod taxonomy and nomenclature at the species level. Steel regarded *Pelorosaurus* as a member of the subfamily Brachiosaurinae, and *P*. *conybeari* and "*P*." *becklesii* as separate valid species of *Pelorosaurus*.

McIntosh [64] regarded *Pelorosaurus* as a valid brachiosaurid that included several species (e.g., *P*. *conybeari* and “*P*. *mackesoni*”). However, he excluded “*P*.” *becklesii* from *Pelorosaurus*, instead considering it to be Sauropoda incertae sedis. This opinion was based largely on limb proportions: ‘The ulna: humerus ratio is 0.71. The latter character and its robustness immediately excludes the animal from the genus *Pelorosaurus* and any other brachiosaurid such as *Pleurocoelus*.’ ([64]: p.398). More recently, most workers have regarded “*P*.” *becklesii* as a titanosaur [1,6,18,36, 75–77]. For example, Upchurch [6] noted the robust nature of the forearm elements and the concave profile of the articular surface of the proximal anteromedial process of the ulna, features which were then believed to be synapomorphies of Titanosauria (see also [1]). Tidwell and Carpenter [75] also regarded “*P*.” *becklesii* as a probable titanosaur and suggested that it shared similarities with an unnamed ‘titanosaur’ from the Cloverly Formation of Wyoming. However, because Tidwell and Carpenter [75] is only a published abstract, there are no further details concerning the character states used to support this claim. The first phylogenetic analysis to include “*P*.” *becklesii* was that of Mannion et al. [18]. This indicated that “*P*.” *becklesii* is a somphospondylan, but only a subset of the analyses supported its inclusion within the Titanosauria. If “*P*.” *becklesii* does indeed represent a titanosaur, then it would be the earliest body fossil material pertaining to a member of that clade from Europe [1,6,36], although trackways from the Middle Jurassic of England are the earliest putative record globally [78,79] (but see [17] for a contrary opinion). In contrast, D’Emic [17] rejected the identification of “*P*.” *becklesii* as a titanosaur, although he did accept it as a titanosauriform. More recently, Poropat et al. [80,81] updated the character scores for the Australian sauropods *Diamantinasaurus* and *Wintonotitan* for the ‘*Lusotitan* Standard Discrete Matrix’ (LSDM) data set presented by Mannion et al. [18]. However, “*Pelorosaurus*” *becklesii* was one of eight taxa that were pruned, *a posteriori*, from the resulting 5334 most parsimonious trees in order to generate an agreement subtree. Thus, Poropat et al. [80,81] did not evaluate the phylogenetic relationships of “*P*.” *becklesii*, and no analysis has examined the impact of the new Australian data on titanosauriform relationships based on the ‘*Lusotitan* continuous and discrete matrix’ (LCDM) data set of Mannion et al. [18].

Most previous workers have acknowledged that “*P*.” *becklesii* represents a distinct taxon (e.g. [1,63,64,66]) but have been reluctant to erect a new generic name because the holotype is somewhat incomplete, and clear autapomorphies have proved to be elusive. Thus, Naish and Martill ([29]: p.499) wrote: '…whether the material is diagnostic is arguable.’ Upchurch et al. [36], however, presented a preliminary reassessment of “*P*.” *becklesii* and concluded that there were at least two potential autapomorphies of the humerus (see below).

In summary, the recent consensus among sauropod workers is that “*P*.” *becklesii* is a distinct taxon that merits a new generic name provided that sufficiently strong autapomorphies can be identified. None of the other sauropod taxa from the Late Jurassic or Cretaceous of Britain can be justifiably referred to, or combined with, “*P*.” *becklesii*. The latter taxon is generally regarded as a member of the Titanosauriformes and is potentially a somphospondylan or even a titanosaur.

## Methods

No permits were required for the described study, which complied with all relevant regulations. The specimens studied for this work are housed at The Natural History Museum, London, UK (institutional abbreviation NHMUK). The specimen catalogue numbers are NHMUK R1868, R1869 and R1870. Other specimens examined in order to make comparisons with *Haestasaurus* are cited in the text as required, with full institutional catalogue numbers and references where appropriate. Cladistic data sets and analytical techniques are outlined in ‘Phylogenetic Analyses’ below.

### Nomenclatural Acts

The electronic edition of this article conforms to the requirements of the amended International Code of Zoological Nomenclature, and hence the new names contained herein are available under that Code from the electronic edition of this article. This published work and the nomenclatural acts it contains have been registered in ZooBank, the online registration system for the ICZN. The ZooBank LSIDs (Life Science Identifiers) can be resolved and the associated information viewed through any standard web browser by appending the LSID to the prefix "http://zoobank.org/". The LSID for this publication is: urn:lsid:zoobank.org:pub: 9D2E9827-D6D5-444A-A01C-69CAE4FFCA22. The electronic edition of this work was published in a journal with an ISSN, and has been archived and is available from the following digital repositories: PubMed Central, LOCKSS, https://iris.ucl.ac.uk/iris/browse/profile?upi=PUPCH49

## Systematic Palaeontology

Sauropoda Marsh, 1878 [47]

Neosauropoda Bonaparte, 1986 [82]

Macronaria Wilson and Sereno, 1998 [9]

*Haestasaurus becklesii* (Mantell) gen. nov. *urn*:*lsid*:*zoobank*.*org*:*act*:*4895F0CD-F39B-4ED0-B1FC-C8D0C3BC45B1*

1888 *Cetiosaurus brevis* Lydekker ([52]: p.58)

1888 *Titanosaurus* Lydekker ([52]: p.58)

1889 *Morosaurus becklesii* Marsh ([55]: p.325)

1889 *Morosaurus brevis* (in part) Nicholson and Lydekker ([56]: p.1179)

1889 *Morosaurus becklesi* Nicholson and Lydekker ([56]: p.1179)

1890 *Morosaurus brevis* (in part) Lydekker ([57]: p.237)

1893 *Morosaurus brevis* (in part) Lydekker ([58]: p.276)

1932 Gen (?) *becklesii* Huene ([63]: p.251, pp.286–288, figs 34, 35)

1936 *Pelorosaurus becklesi* Swinton ([60]: p.211)

1970 *Pelorosaurus becklesii* Steel ([66]: p.68)

1990 “*Pelorosaurus*” *becklesii* McIntosh ([64]: p.398)

1995 “*Pelorosaurus becklesii*” Upchurch ([6]: p.380)

2002 *Pelorosaurus* Tidwell and Carpenter ([75]: p.114A)

2004 “*Pelorosaurus*” *becklesii* Upchurch et al. ([1]: p.398)

2007 “*Pelorosaurus*” *becklesii* Naish and Martill ([29]: p.499)

2011 ‘*Pelorosaurus becklesii*’ Upchurch et al. ([36]: pp.498–501, text-figs 28.8 and 28.9)

2012 “*Pelorosaurus*” *becklesii* D’Emic ([17]: numerous mentions)

2013 “*Pelorosaurus*” *becklesii* Mannion et al. ([18]: numerous mentions])

Holotype— NHMUK R1870, an associated left humerus, ulna and radius, and NHMUK R1868, a portion of skin impression from near the elbow region [22]. N.B. the 1891 catalogue of the Beckles collection [45] mentions the proximal end of a metacarpal that was apparently regarded as part of “*P*.” *becklesii*. It is probable that this catalogue entry refers to the proximal portion of NHMUK R1869. This is actually a large, robust and nearly complete sauropod metacarpal, which is broken into three pieces that fit together. Its relative size (e.g. maximum width of proximal end = 152 mm, total length = 330 mm) means that it cannot belong to the same individual as NHMUK R1870 (see Table 1). None of the literature dealing with “*P*.” *becklesii* prior to, or since, 1891 mentions this metacarpal. We suspect that the metacarpal was not found with the humerus, ulna, radius and skin impression, but became ‘associated’ with them when the Beckles collection was purchased and catalogued by the NHMUK. This would explain why several early papers (e.g. [22]) specifically mention the association of the humerus, ulna, radius and skin impression, but do not note the presence of the metacarpal. Thus, NHMUK R1869 cannot be considered part of the holotype of *Haestasaurus*, or be referred to that taxon, and will not be discussed further here.

Etymology of new generic name—From ‘Haesta’, the name of the putative pre-Roman chieftain whose people apparently settled the area of Hastings and gave the town its name [83], and ‘sauros’, Greek for ‘reptile’.

Locality and horizon—An undetermined horizon within the Hastings Beds Group (late Berriasian—Valanginian [84]), from the coast near Hastings (exact locality unknown), East Sussex, southeast England, United Kingdom (Fig 1).

Diagnosis—*Haestasaurus becklesii* is diagnosed on the basis of the following autapomorphies: (1) the anteromedial corner of the distal end of the humerus projects to form an ‘anterior entepicondylar process’; (2) there are two small vertical ridges situated between the lateral and medial anterodistal processes of the humerus; (3) the proximal articular surface of the radius is widest anteroposteriorly along its lateral margin, and this margin is nearly straight rather than strongly convex; (4) at the distal end of the shaft, the anterior surface of the radius is shallowly concave, between anterolateral and anteromedial ridges; and (5) the combination of a robust ulna (maximum proximal width:proximodistal length ratio > 0.4) and a slender radius (transverse proximal width:proximodistal length ratio < 0.3).

### Description

#### Humerus (Figs 2 and 3, Table 1).

**Fig 2:**
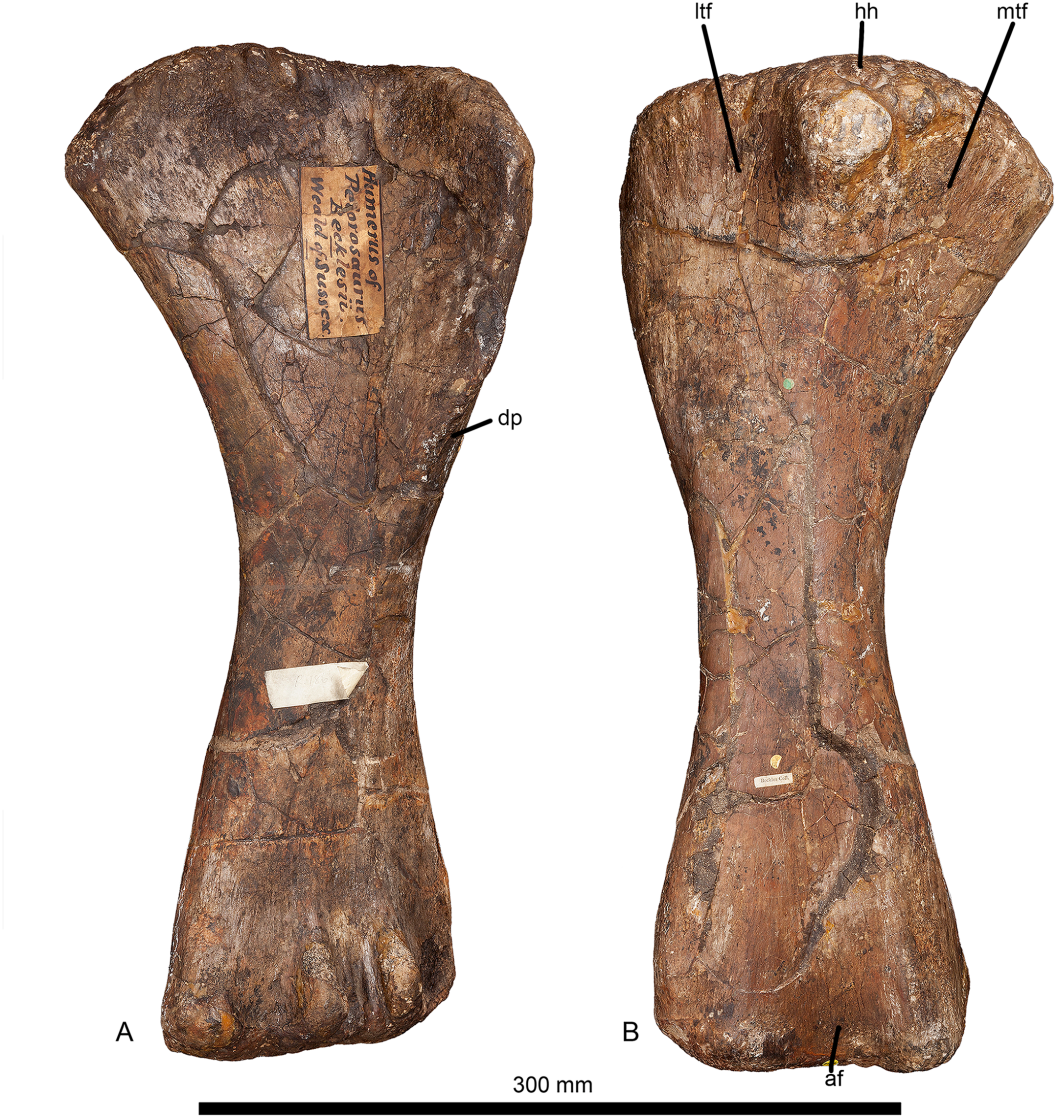
Left humerus of *Haestasaurus becklesii* (NHMUK R1870). A, anterior view; B, posterior view; Abbreviations: af, anconeal fossa; dp, deltopectoral crest; hh, humeral head; ltf, lateral triceps fossa; mtf, medial triceps fossa.

**Fig 3:**
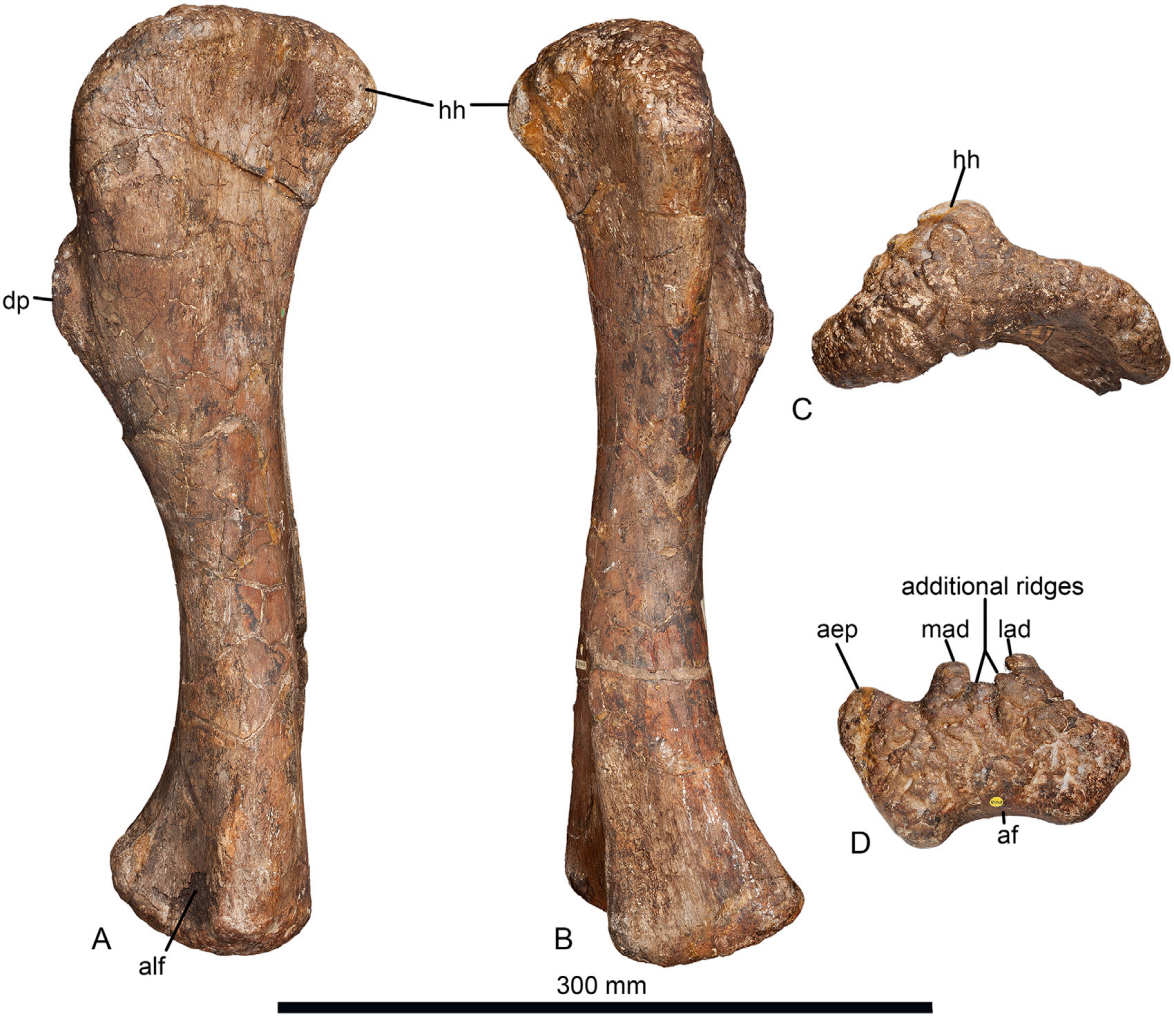
Left humerus of *Haestasaurus becklesii* (NHMUK R1870). A, lateral view; B, medial view; C, proximal end view (posterior surface towards top); D, distal end view (anterior surface towards top). Abbreviations: aep, anterior entepicondylar process; af, anconeal fossa; alf, anterolateral fossa; dp, deltopectoral crest; hh, humeral head; lad, lateral anterodistal process; mad, medial anterodistal process. All parts are at the same scale.

The humerus is virtually complete and unbroken, but it has been slightly crushed anteroposteriorly. This is a relatively robust element: its Robustness Index (RI = the average of the transverse widths at the proximal end, at midshaft and at the distal end, divided by humerus length [see 31]) is 0.33. RI values higher than 0.32 are scored as state 2 in character no. 256 in the data set of Carballido and Sander [19]. Similarly robust humeri mainly occur in titanosaurs (such as *Diamantinasaurus*, *Opisthocoelicaudia*, *Saltasaurus*) and a few diplodocoids (e.g. *Suuwassea*), whereas more gracile humeri (RI = 0.32 or less) are present in most sauropods and basal sauropodomorphs (Table 2).

**Table 2:** Selected ratios for fore limb elements of sauropods.

Taxon	Hafd	Hdpl	Hpdw	HRI	Uppl	Rdmw	Specimen and/or source
*Tazoudasaurus* BS	-	0.50	0.79–0.84	0.20–0.24	~1.5	1.88	[126]
*Vulcanodon* BS	-	-	-	-	1.79	1.17e	[147]
*Barapasaurus* EU	-	0.33	0.75	0.28	1.52	1.71	[148]
*Cetiosauriscus* EU	-	0.47	0.72	0.318	1.77	1.23	NHMUK R3078, PU and PDM pers. obs. (2011)
Chebsaurus EU	-	-	-	-	~1.0	1.75	[149]
*Ferganasaurus* EU	~0.07	0.43	0.74	0.29	1.69e	2.0e	PIN N 3042/1, [101]
*Hudiesaurus* EU	-	-	0.84	-	1.25	-	IVPP V.11121-1 [106], PU and PDM pers. obs. (2007)
*Jobaria* EU	0.1	0.47	0.83	0.26	1.62	1.55	MNN TIG unnumbered, PDM pers. obs. (2010)
*Mamenchisaurus youngi* EU	0.08	0.41	0.71	0.316	1.10 (1.04)	1.81	ZDM 0083 [107]
*Omeisaurus tianfuensis* EU	0.33	0.35	0.71–0.77	0.29	1.0 (1.28)	1.95	ZDM T5701-T5705 [108], PU pers. obs. (1995)
*Shunosaurus* EU	-	0.49	0.78–0.88+	0.29	-	1.69	ZDM T5401, T5402 [115]
*Spinophorosaurus* EU	~0.15	~0.40	~0.9	~0.26	-	-	GCP-CV-4229NMB-1699-R, [98]
*Turiasaurus* EU	-	0.41	0.68	0.25	-	1.97	CPT-1195, PU and PDM pers. obs. (2009)
*Amargasaurus* D	-	0.42	0.66	0.316	1.09	1.51	MACN-N 15, PU and PDM pers. obs. (2013)
*Apatosaurus ajax* D	0.2	0.4	0.8	0.31	1.0 (1.03)	2.17	NMST-PV 20375 [99]
*Dicraeosaurus* D	-	0.41	0.79	0.316	1.25	1.53	MfN NB.R 4912 [87]
*Diplodocus* D	-	-	0.62–0.67	-	1.23 (1.03)	1.68	AMNH 380, AMNH 695, HMNS 175, USNM 10865 [150], WDC BS-001A [151]
*Limaysaurus* D	-	-	0.57	-	1.35	1.47	[5], PDM pers. obs. (2009)
Nigersaurus D	0.14	0.36	0.65	0.26	1.4 (1.18)	1.65	MNN G33-2, G33-8, PDM pers. obs. (2010)
*Suuwassea* D	0.1	0.48	0.76	0.38	-	-	ANS 21122 [102]
*Tornieria* D	-	0.42	0.72–0.75	0.3	1.27	-	MfN MB.R 2586, 2672, 2673 [150]
*Aragosaurus* MN	-	-	0.80e	0.25e	1.11 (1.11)	1.98	I.G. 483, 484, 490 [111]
*Camarasaurus grandis* MN	0.2	0.42	0.7	0.3	1.34 (1.34)	2.33	YPM 1901 [100]
*Haestasaurus* MN	0.14	0.35	0.79	0.33	1.29 (1.16)	1.85	NHMUK R1870, PU and PDM pers. obs. (2012)
*Lourinhasaurus* MN	-	0.42	0.75	0.26	1.32	1.69	MGIGM uncatalogued, PU and PDM pers. obs. (2009)
*Tehuelchesaurus* MN	-	0.48	0.61	0.31	1.5 (1.25)	2.11	MPEF-PV 1125, PU and PDM pers. obs. (2013)
“*Astrodon johnstoni*” TF	~0.18	0.25	0.95	0.27	-	~2.0	USNM 2263 [97]
*Cedarosaurus* TF	-	-	0.96	0.21	2.56 (2.88)	~1.7	DMNH 39045 [94]
*Chubutisaurus* TF	-	0.4	0.87	0.26	-	2.0	MACN 1822/32 [14]
*Giraffatitan* TF	0.5	0.35	0.8	0.21	1.43 (1.41)	2.0	MfN MB.R. 2181 [87]
*Pelorosaurus conybeari* TF	0.08	0.37e	-	0.23e	-	-	NHMUK 28626, PU and PDM pers. obs. (2012)
*Alamosaurus* TT	-	0.45e	0.85e	0.32e	1.23 (1.32)	2.63	USNM 15560 [92], PU and PDM pers. obs. (2008)
*Argyrosaurus* TT	-	0.45	0.83e	0.3e	1.03	1.3	MLP 77-V-29-1 [77]
*Diamantinasaurus* TT	-	0.44	0.76	0.34	1.43 (1.0)	2.15	AAOD 603 [80]
*Elaltitan* TT	~0.5	0.45	0.87e	0.31	1.25	~1.75	PVL 4628 [77]
*Epachthosaurus* TT	~0.5	~0.35	1.01	0.29	2.0	2.18	UNPSJB-PV 920, PU and PDM pers. obs. (2013)
*Malawisaurus* TT	~0.45	0.37	0.72–0.83	0.26–0.29	1.42 (1.05)	2.31	Mal-41, 289, 316, 317 [95]
*Opisthocoelicaudia* TT	~0.3	0.39	0.67–0.77	0.36–0.41	1.64 (0.86)	2.06	Z.Pal MgD-I/48 [27]
*Rapetosaurus* TT	~0.45	0.47	0.7	0.27	1.63 (1.14)	1.91	FMNH PR 2209 [91]
*Saltasaurus* TT	-	0.46	0.72	0.42	1.47 (?)	1.73	PVL 4017–63, 4017–74, PU and PDM pers. obs. (2013)

Ratio abbreviations: Hafd, humeral anconeal fossa depth divided by the anteroposterior width of the distal end (see Fig 12 for ratio definition); Hdpl, distance from proximal end of humerus to most prominent point of deltopectoral crest divided by humerus proximodistal length; Hpdw, transverse width of the distal end of the humerus divided by the transverse width of the proximal end; HRI, humeral Robusticity Index (sensu [31] = the average of the transverse widths of the humerus at the proximal end, midshaft and distal end, divided by humerus proximodistal length); Uppl, length of the anteromedial process of the proximal ulna divided by the length of the anterolateral process (see ‘RC3’ and Fig 13 for definition of lengths. N.B. values in parentheses are those obtained by Mannion et al. [18]); Rdmw, transverse width of the distal end of the radius divided by the transverse width at midshaft. Other abbreviations: BS, basal sauropod (i.e. non-eusauropod sauropods); D, diplodocoid; e, estimated value; EU, basal eusauropod (i.e. non-neosauropod eusauropods); MN, basal macronarian (i.e. non-titanosauriform macronarians); TF, titanosauriform (i.e. non-titanosaurian titanosauriforms); TT, titanosaurian.

The proximal articular surface is strongly rugose (Fig 3C). The proximolateral corner lies only slightly below the level of the humeral head, whereas the medial half of the proximal articular surface is convex and curves strongly medially and distally in anterior view (Fig 2). Thus, *Haestasaurus* possesses the ‘square’ proximolateral corner that occurs in *Tehuelchesaurus* and most somphospondylans, rather than the plesiomorphic rounded condition observed in other taxa [1,10,17,18,85]. The profile of the proximal end, in anterior view, lacks the strongly sigmoid curvature that occurs in several titanosaurs [7,86] such as *Diamantinasaurus* (AAOD 603 [80]: fig 9A and 9E), *Opisthocoelicaudia* (Z.PAL MgD-I/48 [27]: fig 7B and 7D) and *Saltasaurus* (PVL 4017–63, PU and PDM pers. obs. 2013), though a mild version of this condition is present in *Haestasaurus* (Fig 2A). In proximal end view the lateral half of the articular surface curves strongly anterolaterally towards the proximolateral corner and top of the deltopectoral crest, whereas the medial half is nearly straight and projects medially. There are no large fossae or major processes along the junction of the proximal and anterior faces, although some small rugosities do occur (especially on the medial half). The anteroposteriorly widest part of the proximal end is located at about mid-width (slightly nearer the medial than the lateral margin), where the humeral head expands prominently backwards. Here, the proximal humeral head forms a prominent process that overhangs the posterior surface of the shaft (Fig 3A and 3B): a similarly distinct process is also present in some titanosauriform taxa, such as *Giraffatitan* ([87]: Beilage A, fig 1b) and *Ligabuesaurus* ([88]: fig 6a). This has a rugose articular surface that curves posterodistally in lateral view. Distally, this process gives rise to a stout vertical ridge that fades out very rapidly into the posterior surface, but still extends downwards to divide this part of the proximal end into lateral and medial fossae. These fossae (Fig 2B) probably represent the origins of the lateral and medial heads of the triceps muscle [27]. On the posterior surface, approximately level with the most prominent point of the deltopectoral crest, there is a low, rounded and vertically elongated bulge situated a short distance laterodistal to the distal end of the ridge that separates the triceps fossae. This bulge is subtle but can be seen in lateral view (Fig 3A). Based on the muscle reconstructions of Borsuk-Bialynicka [27], this is probably the insertion for the M. latissimus dorsi. A second striated muscle scar is located a short distance proximolateral to the one just described, and probably marks the insertion of the M. scapulohumeralis anterior ([27]: fig 7C and 7D). These two clearly marked muscle insertions are absent on most sauropod humeri, including the titanosaur *Argyrosaurus* (MLP 77-V-29-1 [77]: fig 2B), but in many titanosaurs (e.g. *Elaltitan*, PVL 4628 [77]: fig 6F; *Epachthosaurus*, UNPSJB-PV 920 [89]; *Magyarosaurus*, NHMUK 3864 [PU pers. obs. 2011]; *Opisthocoelicaudia*, Z.PAL MgD-I/48 [27]: fig 7, *Neuquensaurus*, MLP-CS 1050 [90]: fig 3C and 3H; *Rapetosaurus*, FMNH PR 2209 [91]: fig 35C,D) there is a prominent striated projection in this region that has usually been identified as the insertion for the M. latissimus dorsi [90], or for ‘brachial musculature’ [91]. In these titanosaurs, the attachment for the M. scapulohumeralis anterior forms a prominent lateral projection that is visible in anterior view (see ‘New and Revised Characters’ below), but this projection does not occur in *Haestasaurus*.

The anterior surface of the proximal half of the humerus is mediolaterally concave. This surface does not display the low rounded bulge or rugosity that marks the site of insertion of the M. coracobrachialis, but this might be because of the relatively small size of *Haestasaurus* and/or the presence of a museum label that potentially covers this structure. This muscle scar occurs in most neosauropods, and seems to be particularly well developed in titanosaurs such as *Diamantinasaurus* (AAOD 603 [80]), *Elaltitan* (PVL 4628 [77]: fig 6), *Neuquensaurus* (MLP-CS 1050 [91]) and *Opisthocoelicaudia* (Z.PAL MgD-I/48 [27]: fig 7). In *Haestasaurus*, the deltopectoral crest is most prominent at approximately 0.35 of humerus length from the proximal end. Although the deltopectoral crest does not extend as far distally as in most sauropods, we do not regard this as a diagnostic character state because it might be related to the small size of *Haestasaurus*, and a few other taxa (e.g. *Barapasaurus*, *Epachthosaurus*, *Nigersaurus*, *Omeisaurus*) have similar values (Table 2). The crest is a transversely thin plate in its most prominent section, projecting anteriorly and slightly laterally (Fig 2A). Thus the deltopectoral crest of *Haestasaurus* is restricted to the lateral margin (i.e., the plesiomorphic state present in most sauropods and some titanosauriforms) rather than displaying the derived medial deflection of the distal part observed in certain titanosauriforms such as *Alamosaurus* (USNM 15560 [92]: fig 5), *Angolatitan* (MGUANPA-003 [93]: fig 3Bb), *Cedarosaurus* (DMNH 39045 [94]: fig 7, PDM pers. obs. 2008), *Giraffatitan* (MfN MB.R. 2181 [87]: Beilage A, fig 1a), *Magyarosaurus* (NHMUK 3864, PU pers. obs. 2011) and *Opisthocoelicaudia* ([27]: fig 7) (see [1,7,10,18]). In *Haestasaurus*, the posterolateral surface of the humerus (posterior to the deltopectoral crest) forms a broad rounded ridge that extends distally. Between this ridge and the lateral surface of the deltopectoral crest there is therefore a shallow vertical fossa.

The humerus displays little torsion along the shaft, resulting in the long-axes across the proximal and distal articular faces being approximately parallel to each other. In medial view, the posterior margin of the humerus is strongly concave. Both the lateral and medial margins of the diaphysis are concave in anterior view (Fig 2A). Possession of a straight lateral margin is a derived state that occurs in some titanosauriforms, such as *Alamosaurus* (USNM 15560 [92]: fig 5), *Cedarosaurus* (DMNH 39045 [94]: fig 7, PDM pers. obs. 2008), *Giraffatitan* (MfN MB.R. 2181 [87]: Beilage A, fig 1a) and *Malawisaurus* (MAL-221, MAL-289 [95]: fig 20B,D,E), but most sauropods possess a concave margin, and this is retained (or reacquired) in titanosaurs such as *Opisthocoelicaudia* (Z.PAL MgD-I/48 [27]: fig 7) and *Saltasaurus* (PVL 4017–67 [96]: fig 31a,c, PU and PDM pers. obs. 2013) (see [11,18]). The midshaft is wider transversely than anteroposteriorly, and has a rounded ‘D’-shaped cross-section with a flattened, but still mildly convex, anterior face.

**Fig 4:**
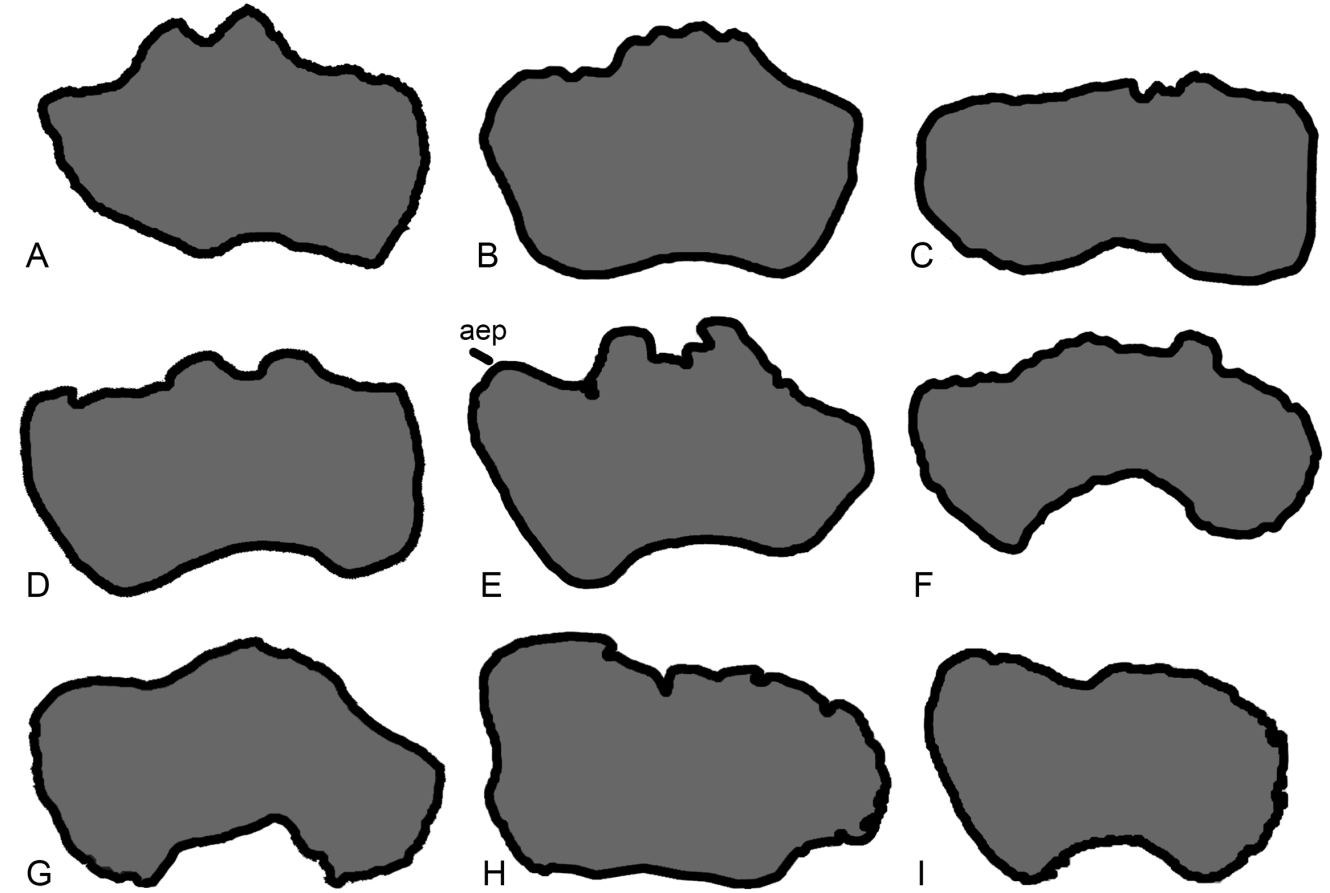
Exemplar profiles of the distal ends of sauropod left humeri (anterior surfaces towards top). A, *Mamenchisaurus youngi* (ZDM 0083 [107]); B, *Ferganasaurus* (PIN 3042/1 [101]): C, *Apatosaurus excelsus* (YPM 1980 [100]); D, *Camarasaurus grandis* (YPM 1901 [100]); E, *Haestasaurus* (NHMUK R1870); F, *Giraffatitan* (MfN MB.R 2181 [87]: a right humerus that has been reversed to facilitate comparison); G, *Epachthosaurus* (UNPSJB-PV 920, based on a photograph by PDM); H, *Diamantinasaurus* (AAOD 603 [80]); I, *Neuquensaurus* (MLP-CS 1050 [90]). Abbreviation: aep, anterior entepicondylar process. Profiles not drawn to the same scale.

**Fig 5:**
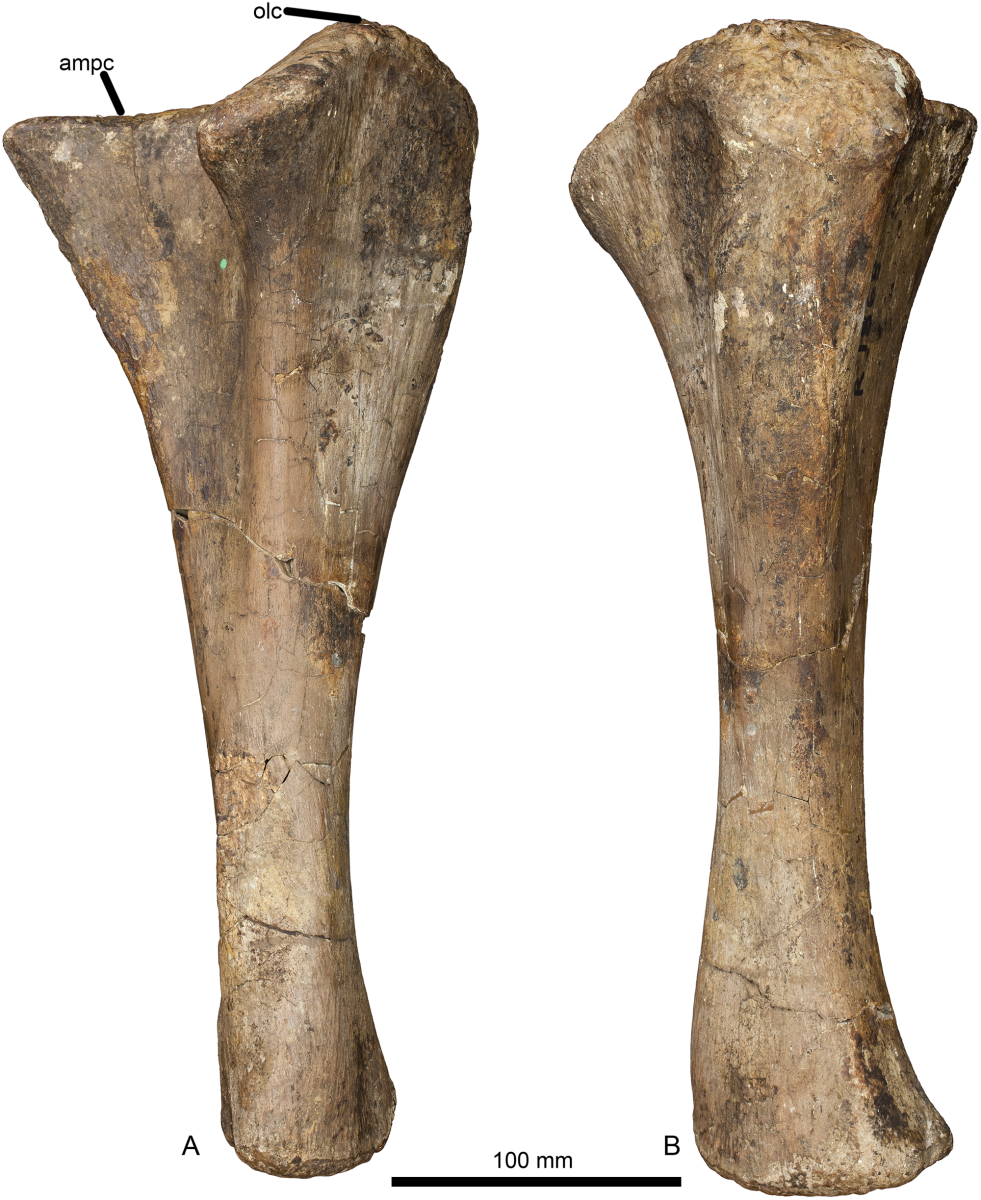
Left ulna of *Haestasaurus becklesii* (NHMUK R1870). A, anterolateral view (with anteromedial process directed mainly medially); B, posterior view (with posterior process directed towards the observer). Abbreviations: ampc, concave surface of the anteromedial process; olc, olecranon.

**Fig 6:**
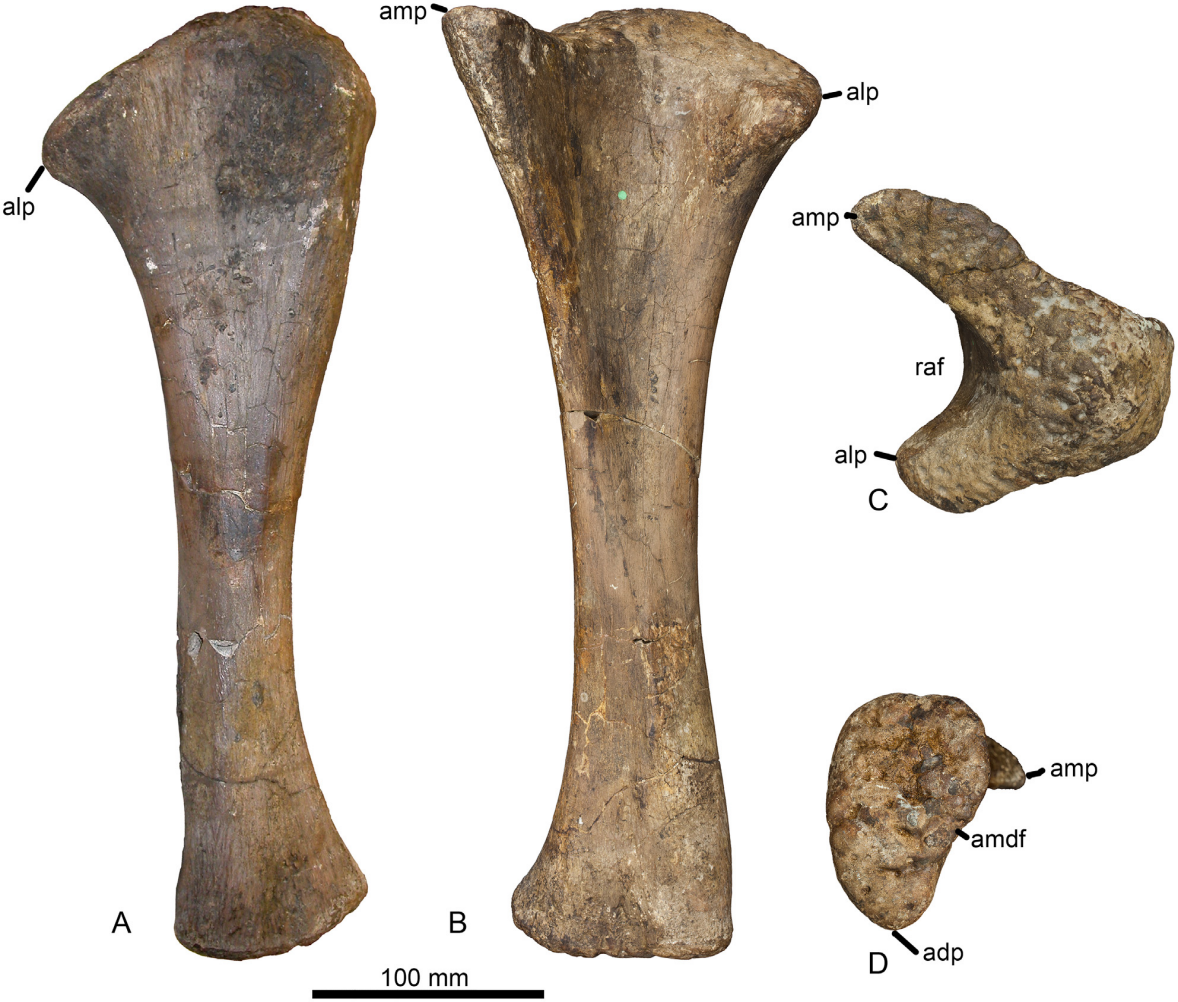
Left ulna of *Haestasaurus becklesii* (NHMUK R1870). A, lateral view; B, anteromedial view; C, proximal view; D, distal view (N.B. the anteromedial process of the proximal end is visible in this view). Abbreviations: adp, anterior distal process; alp, anterolateral process of the proximal end; amdf, anteromedially facing fossa immediately above the distal end; amp, anteromedial process of the proximal end; raf, fossa for reception of the proximal end of the radius. All parts are at the same scale.

The posterior surface of the distal shaft bears a moderately deep anconeal (= ‘cubital’ or ‘supracondylar’) fossa bounded by two vertical ridges, the medial one being more prominent and acute, and the lateral one more rounded. This asymmetry in the ridges bounding the anconeal fossa occurs in several titanosaurs, including *Argyrosaurus* (MLP 77-V-29-1 [77]: p.616) and *Neuquensaurus* (MLP-CS 1050 [90]: fig 3, PU and PDM pers. obs. 2013). An enlarged anconeal fossa occurs in many somphospondylans [1,36], but see ‘New and Revised Characters’ below, for quantification and further evaluation of this character. In anterior view, the distal end projects nearly as far medially as the medial process of the proximal end. This feature was proposed as an autapomorphy of *Haestasaurus* by Upchurch et al. [36], but many sauropods display some degree of medial flaring of the distal humerus (e.g. "*Astrodon johnstoni*”, USNM 2263 [97]: fig 3.10), and the comparative measurements in Table 2 demonstrate that the distal end of the *Haestasaurus* humerus is not noticeably wider relative to the proximal end than in other sauropods. We therefore propose abandonment of this character state as an autapomorphy of *Haestasaurus (contra* [36]). The anterior face of the distal end of the humerus bears two prominent processes located just medial and lateral to the midline. These processes (here termed the lateral and medial anterodistal processes, Fig 3D, see [98]) occur in most sauropods, including *Apatosaurus ajax* (NSMT-PV 20375 [99]: fig 5F), *Camarasaurus grandis* (YPM 1901 [100]: pl. 49, fig 5), *Hudiesaurus* (IVPP V.11121-1, PU and PDM pers. obs. 2007), *Turiasaurus* (CPT-1195, PU and PDM pers. obs. 2009), and *Lourinhasaurus* (MGIGM uncatalogued, PU and PDM pers. obs. 2009), but are apparently coalesced into a single reduced process in *Chubutisaurus* and most titanosaurs [14,17] (see Fig 4). In *Haestasaurus*, between these prominent processes, there are two smaller vertical ridges located virtually on the midline (Fig 3D). These two ridges have not been observed in any other sauropod, except possibly one such ridge in *Apatosaurus excelsus* (YPM1980 [100]: pl. 48, fig 4) and *Diamantinasaurus* (AAOD 603 [80]: fig 13B, PU and PDM pers. obs. 2012), and this feature is therefore provisionally regarded as an autapomorphy of *Haestasaurus*. The anteromedial corner of the distal end is also drawn out into an anterior process (here termed the anterior entepicondylar process: Fig 3D). The latter process projects almost as far anteriorly as the lateral and medial anterodistal processes described above. This anterior entepicondylar process is absent in other sauropods, including, for example, *Apatosaurus ajax* (NSMT-PV 20375 [99]: fig 5), “*Astrodon johnstoni*” (USNM 2263 [97]: fig 3.10), *Camarasaurus grandis* (YPM1901 [100]: pl. 49, fig 5), *Elaltitan* (PVL 4628 [77]: fig 6E), *Epachthosaurus* (UNPSJB-PV 920, PU and PDM pers. obs. 2013), *Ferganasaurus* (PIN N 3042/1 [101]: fig 6F), *Giraffatitan* (MfN MB.R. 2181 [87]: Beilage A, fig 1e), *Opisthocoelicaudia* (Z.PAL MgD-I/48 [27]: pl.8, fig 3), *Patagosaurus* (MACN 932, PU and PDM pers. obs. 2013), *Rapetosaurus* (FMNH PR 2209 [91]: fig 35F), *Suuwassea* (ANS 21122 [102]: fig 4.3) and *Tehuelchesaurus* (MPEF-PV 1125 [15]: fig 15C, PU and PDM pers. obs. 2013) (see Fig 4 for comparative distal end views of exemplar sauropod humeri). Because the anterior entepicondylar process of *Haestasaurus* has not been observed in any other taxon (except for a much broader and more rounded version in *Diamantinasaurus* AAOD 603 [80]: fig 13B, *Neuquensaurus* MLP-CS 1050 [90]: fig 3D and 3G, and *Saltasaurus* PVL 4017–63 [PU and PDM pers. obs. 2013]), it is provisionally regarded as an autapomorphy. The distalmost part of the medial surface is flat and faces medially. This surface is particularly wide anteroposteriorly because it is extended by the anterior entepicondylar process. The lateral surface of the distal shaft forms a vertical ridge that is rounded anteroposteriorly. This ridge projects laterally, defining the posterior wall of a broad and deep anterolateral fossa (Fig 3A). Such a ridge and fossa are frequently present in sauropod humeri (e.g. *Diamantinasaurus* (PU and PDM pers. obs. 2012) and *Saltasaurus* [PVL 4017–63, PU and PDM pers. obs. 2013]). The depth of this fossa is variable, reflecting the relative prominence of the lateral anterodistal process and lateral ridge (and perhaps also post-mortem crushing in some cases).

**Fig 7:**
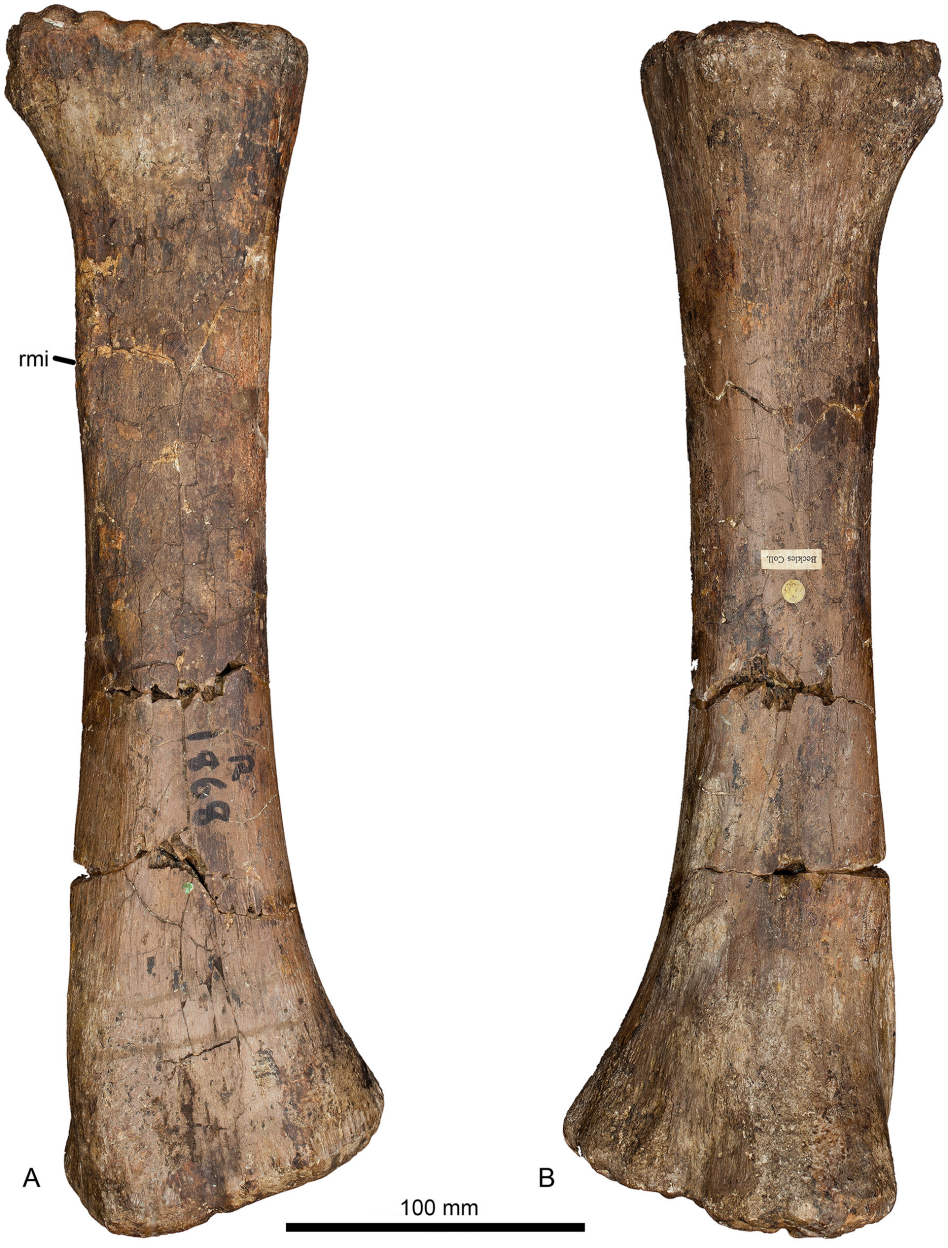
Left radius of *Haestasaurus becklesii* (NHMUK R1870). A, anterior view; B, posterior view. Abbreviation: rmi, ridge for muscle insertion (for the tendon from the combined M. biceps and M. brachialis inferior).

The distal articular surface of the humerus is rugose and mildly convex anteroposteriorly: thus, it does not curl strongly up onto the anterior and posterior surfaces of the shaft unlike the derived condition observed in many titanosaurs, especially saltasaurids such as *Saltasaurus* and *Neuquensaurus* [103]. In anterior view, the distal articular surface is nearly flat (Fig 2A): thus *Haestasaurus* lacks the division of the distal humerus into distinct rounded ulnar and radial condyles that occurs in several derived titanosaurs such as *Opisthocoelicaudia* (Z.PAL MgD-I/48 [27]: fig 7B and 7D) and *Saltasaurus* (PVL 4017–63, PU and PDM pers. obs. 2013) (see character no. 164 in [10]).

#### Ulna (Figs 5 and 6, Table 1)

The ulna is complete but was broken and repaired at approximately midlength. As noted by Upchurch [6], the *Haestasaurus* ulna is a relatively robust element. For example, the maximum width of the proximal end of the ulna is 0.44 of the proximodistal length of the element. According to Wilson ([10]: character no. 168), values of this ratio above 0.4 represent a synapomorphy of *Isisaurus*+Saltasauridae, although he also noted that this occurs independently in some non-neosauropod taxa such as *Mamenchisaurus*. Robust ulnae also occur in *Apatosaurus louisae* (CM 3018 [104]), *Bellusaurus* ([105]: fig 9) and *Hudiesaurus* (IVPP V11121-1 [106]): thus, some caution is required when interpreting the robust ulna of *Haestasaurus* as evidence for titanosaurian affinities.

The proximal articular surface is generally rugose. In proximal end view (Fig 6C), the ulna is ‘V’-shaped, with an anteromedial process that is 1.29 times as long as the anterolateral process (Table 2). D’Emic [17] suggested that *Haestasaurus* possesses a derived state that is also seen in other titanosauriforms, in which the anteromedial process is longer than the anterolateral one: however, the values for this ratio shown in Table 2 cast some doubt on the validity of this character as an indicator of titanosauriform affinities (see ‘New and Revised Characters’ below for further consideration of this issue).

**Fig 8:**
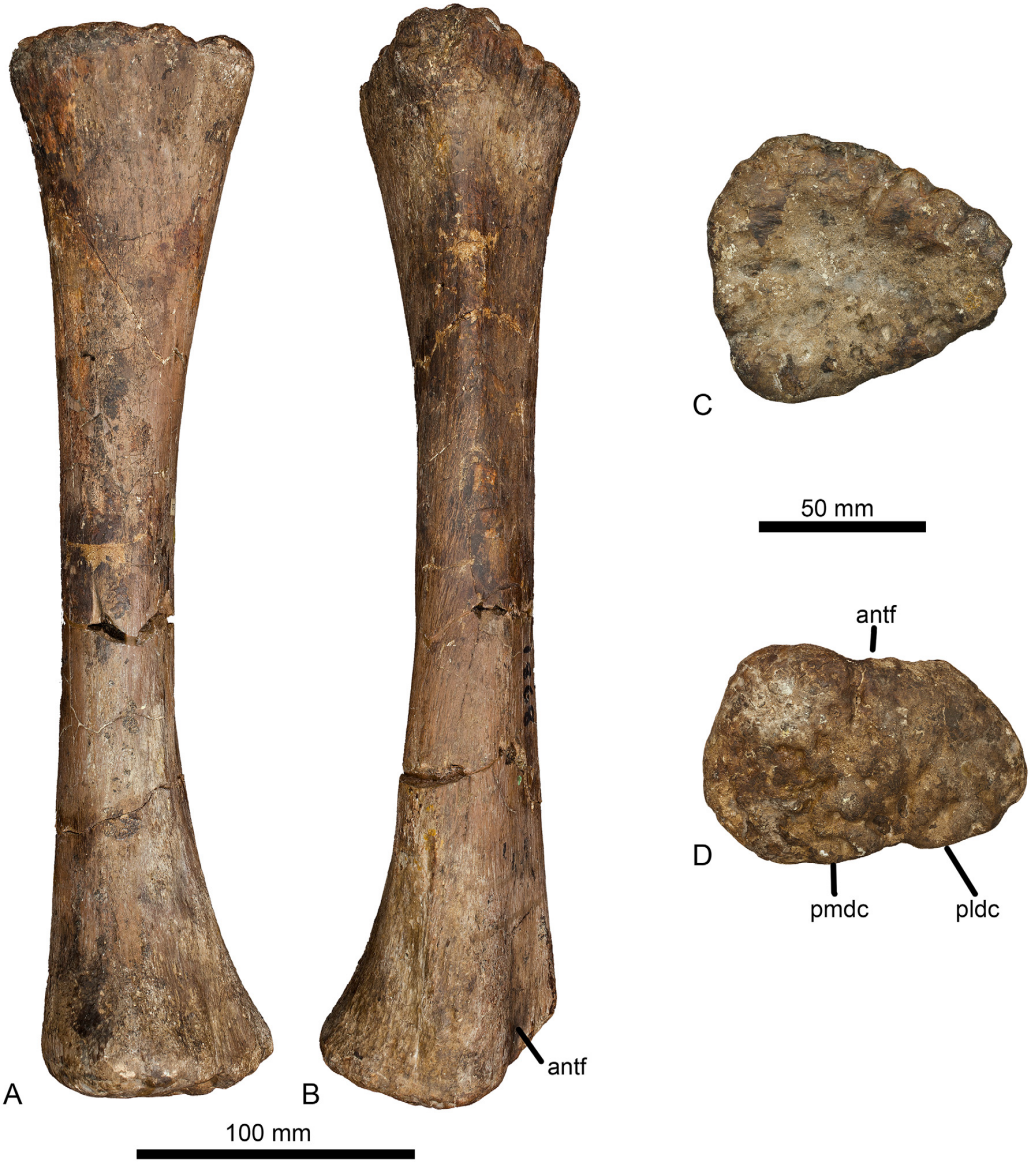
Left radius of *Haestasaurus becklesii* (NHMUK R1870). A, lateral view; B, medial view; C, proximal end view (anterior margin towards top); D, distal end view (anterior margin towards top). Abbreviations: antf, anterior fossa; pldc, posterolateral distal condyle; pmdc, posteromedial distal condyle. A and B are at the same scale; C and D are at the same scale.

**Fig 9:**
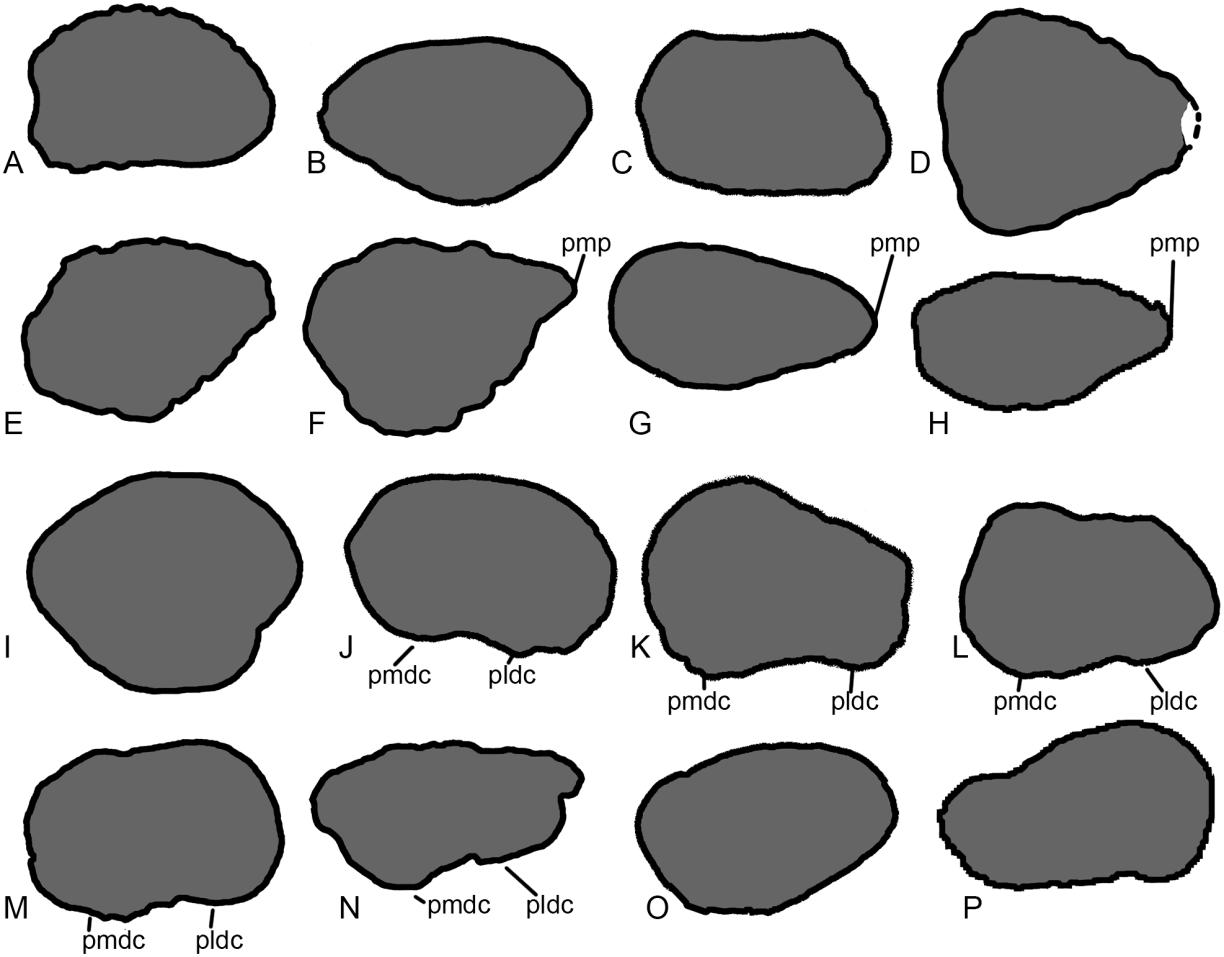
Comparisons of sauropod proximal and distal radii. Exemplar profiles of the proximal (A-H) and distal (I-P) ends of sauropod left radii (anterior surfaces towards top): A, I, *Ferganasaurus* (PIN 3042/1 [101]): B, J, *Apatosaurus excelsus* (YPM 1980 [100]); C, K, *Camarasaurus grandis* (YPM 1901 [100]); D, L, *Haestasaurus* (NHMUK R1870); E, M, *Giraffatitan* (MfN MB.R 2181 [87]); F, N, *Epachthosaurus* (UNPSJB-PV 920, based on photographs by PDM); G, O, *Diamantinasaurus* (AAOD 603 [80]); H, P, *Neuquensaurus* (MLP-CS 1169 [90]; P based on a photograph provided y S. Poropat). B, E-H, J, and M-P are based on right radii that have been reversed in order to facilitate comparison. Abbreviations: pldc, posterolateral distal condyle; pmdc, posteromedial distal condyle; pmp, proximal medial process. Profiles not drawn to the same scale.

The olecranon of the *Haestasaurus* ulna is well developed as a rounded region that is higher than the articular surfaces of the anterior processes. The articular surface of the anteromedial process is concave along its length (Fig 5A) and flat transversely. Both the prominent olecranon and concave anteromedial process are derived states that are moderately well-developed in some basal titanosauriforms, including the brachiosaurid *Giraffatitan* and the somphospondylan *Sauroposeidon* (= *Paluxysaurus*: see [17,18]), and more strongly marked in advanced titanosaurs such as *Alamosaurus* (USNM 15560 [92]: fig 9), *Diamantinasaurus* (AAOD 603 [80]: fig 15), *Opisthocoelicaudia* (Z.PAL MgD-I/48 [27]: fig 8) and *Saltasaurus* (PVL 4017–74, PU and PDM pers. obs. 2013). On this basis, D’Emic [17] argued that *Haestasaurus* is likely to be a member of the Titanosauriformes: however, it should be noted that a distinct olecranon and concave anteromedial process also occur in some probable non-titanosauriforms, such as *Hudiesaurus* (IVPP V.11121-1 [106], PU and PDM pers. obs. 2007) and *Janenschia* (MfN MB.R.2095.11 [87]: pl. 17, fig 7a; see also [6,17,18]). In *Haestasaurus*, the surface of the anteromedial process does not slope strongly distally, unlike the condition observed in some basal macronarians such as *Tehuelchesaurus* and *Lusotitan* (character no. 235 in [18]). The anterolateral process has an articular surface that is flat both transversely and longitudinally, but slopes quite strongly downwards towards its tip, as occurs in other sauropods such as *Epachthosaurus* (UNPSJB-PV 920 [89] fig 9C, PU and PDM pers. obs. 2013), *Opisthocoelicaudia* (Z.PAL MgD-I/48 [27]: fig 8A) and *Saltasaurus* (PVL 4017–74, PU and PDM pers. obs. 2013), though not *Diamantinasaurus* (AAOD 603 [80]: fig 15). In proximal end view (Fig 6C), the anterolateral and anteromedial processes of the *Haestasaurus* ulna are at approximately 80° to each other, although the tip of the anterolateral process curls slightly medially, giving the impression of a more acute angle. An angle of 80° or less occurs in most non-titanosauriforms (e.g. *Camarasaurus grandis* YPM1901 [100]: pl. 53, fig 3a; *Mamenchisaurus youngi*, ZDM0083 [107]; fig 36C), although some basal eusauropods (e.g. *Omeisaurus tianfuensis*, ZDM T5704 [108]: fig 46B) and one species of *Apatosaurus* (*A*. *parvus*, [99]) have proximal processes at right-angles to each other. An angle of greater than 80° is usually present in titanosauriforms, including *Giraffatitan* (MfN MB.R. 2181 [87]: Beilage A, fig 2c), *Opisthocoelicaudia* (Z.PAL MgD-I/48 [27]: pl. 7, fig 5) and *Rapetosaurus* (FMNH PR 2209 [91]: 37D). In *Haestasaurus*, both processes are transversely narrower near their bases and then widen slightly towards their tips, before tapering to points. The third, posterior, process of the proximal end is relatively small, unlike the large processes that occur in some titanosaurs, such as *Diamantinasaurus* (AAOD 603 [80]: fig 15F) and *Saltasaurus* (PVL 4017–74, PU and PDM pers. obs. 2013) (see ‘New and Revised Characters’ below). In *Haestasaurus*, the posterior process gives rise to a vertical ridge that is wide mediolaterally and has an almost flat surface at its proximal end, becoming less prominent and more rounded distally. The anterolateral and anteromedial processes also produce prominent vertical ridges that extend distally and define the fossa for reception of the proximal end of the radius. The anterolateral ridge is rounded throughout its length, whereas the anteromedial ridge is sharper. A weak rugosity is present in the strongly concave radial fossa. Close to the proximal end, the lateral surface of the anterolateral process is mildly concave, whereas the medial surface of the anteromedial process is more strongly concave in a region that lies further distally.

The subtriangular cross-section of the proximal end persists along the shaft up to approximately midlength. Here a sharp interosseous ridge develops on the anterior face and so transforms the cross-section into a square with rounded corners. Posteromedial to the interosseous ridge, the anteromedial surface is shallowly concave. This concavity extends distally and merges into the mildly concave area that faces anteromedially and lies immediately above the distal end itself. This anteromedial concavity articulates with the distal part of the posterolateral surface of the radius. There is no vertical ridge and associated groove on the posterolateral surface of the ulna, immediately above the distal end, unlike the ulnae of *Losillasaurus* and *Turiasaurus* [109,110]. Towards the distal end, the ulna widens markedly anteroposteriorly, mainly as a result of posterior expansion (Fig 6A and 6B): thus, *Haestasaurus* lacks the derived unexpanded distal end that occurs in some titanosaurs such as *Diamantinasaurus* (AAOD 603 [80]: fig 15) and *Epachthosaurus* (UNPSJB-PV 920 [89]: fig 9B and 9C, PU and PDM pers. obs. 2013) (see [17]). In distal view (Fig 6D), the articular surface of the *Haestasaurus* ulna has a comma-shaped outline, with a strongly rounded posterior margin and tapering anterior process that bears the anteromedial fossa for the radius (this character might be phylogenetically informative—see ‘New and Revised Characters’ below). The distal end surface is mildly convex both anteroposteriorly and transversely, and is strongly rugose, as is typical for most sauropods.

#### Radius (Figs 7 and 8, Table 1)

The radius is virtually complete, lacking only the tip of the medial process of the proximal end (Figs 7 and 8). This element was originally recovered intact, but was broken into three portions that fit together at the breaks. These breaks have been repaired recently.

The radius is a relatively slender element, with a proximal transverse width to shaft length ratio of approximately 0.25 (Table 1). Such slender radii represent the plesiomorphic state, in contrast to the robust elements (proximal width to length ratio equals 0.30 or higher) observed in titanosaurian sauropods [6] and some probable non-titanosaurs (e.g. *Aragosaurus*, I.G. 484 [111]: fig 9; *Hudiesaurus*, IVPP V.11121-1 [106]: fig 2; *Janenschia*, MfN MB.R.2095.9 [87]: pl. 17, fig 8). Typically, sauropods with robust ulnae (see above) also have robust radii. However, *Haestasaurus* appears to be unique in possessing a robust ulna but a relatively slender radius, and this combination of character states is provisionally regarded as autapomorphic. As in most sauropods, the transverse width of the proximal articular surface is greater than its maximum anteroposterior width in *Haestasaurus* (Fig 8C, Table 1). Also, *Haestasaurus* lacks the derived condition seen in *Turiasaurus* and *Zby* in which the anteroposterior width of the proximal end of the radius is less than 0.5 that of the distal end [112]. There is a small degree of twisting between the proximal and distal ends of the radius, such that the long-axes of the two surfaces are not quite in the same plane: however, *Haestasaurus* does not display the strong twisting (greater than 45°) that occurs in the radii of taxa such as *Epachthosaurus* (UNPSJB-PV 920, PU and PDM pers. obs. 2013), *Huabeisaurus* (HBV-20001 [113]: fig 19), *Malawisaurus* [95] and *Rapetosaurus* (FMNH PR 2209 [91]: fig 36C,D) (see [18]). The proximal articular surface is rugose (especially towards its margins) and mildly concave centrally. It has a subtriangular outline, created by an anteroposteriorly wide and nearly straight lateral margin and a tapering medial process (Fig 8C). In other sauropods such as *Diamantinasaurus* (AAOD 603 [80]: fig 12D), *Epachthosaurus* (UNPSJB-PV 920, PU and PDM pers. obs. 2013), *Patagosaurus* (MACN 932, PU and PDM pers. obs. 2013) and *Tehuelchesaurus* (MPEF-PV 1125, PU and PDM pers. obs. 2013), the greatest anteroposterior width of the proximal radius occurs close to the midline of the element and the proximal end has an elliptical or oval profile (Fig 9; see ‘New and Revised Characters’ below). Therefore, we regard the nearly straight and anteroposteriorly widened lateral margin as a potential autapomorphy of *Haestasaurus*. Curry Rogers ([11]: character no. 282, state 2) (see also character no. 46 in [18]) noted that the transverse width of the proximal end of the radius is less than that of the distal end in some titanosaurs, such as *Alamosaurus* and *Rapetosaurus*. The ratio of proximal end:distal end transverse width in *Haestasaurus* is > 0.89 (Table 1): the ‘>‘ reflects the fact that this is a minimum value because a small part of the proximal end is missing. When complete, it seems probable that this ratio was still less than 1.0 (estimated at 0.92), suggesting that *Haestasaurus* shares the derived state with several titanosaurs. However, this condition also occurs in some non-titanosaurs, including *Mamenchisaurus youngi* (ZDM 0083 [107) and *Tehuelchesaurus* (MPEF-PV 1125, PU and PDM pers. obs. 2013), so caution is required when interpreting the phylogenetic significance of this character.

**Fig 10:**
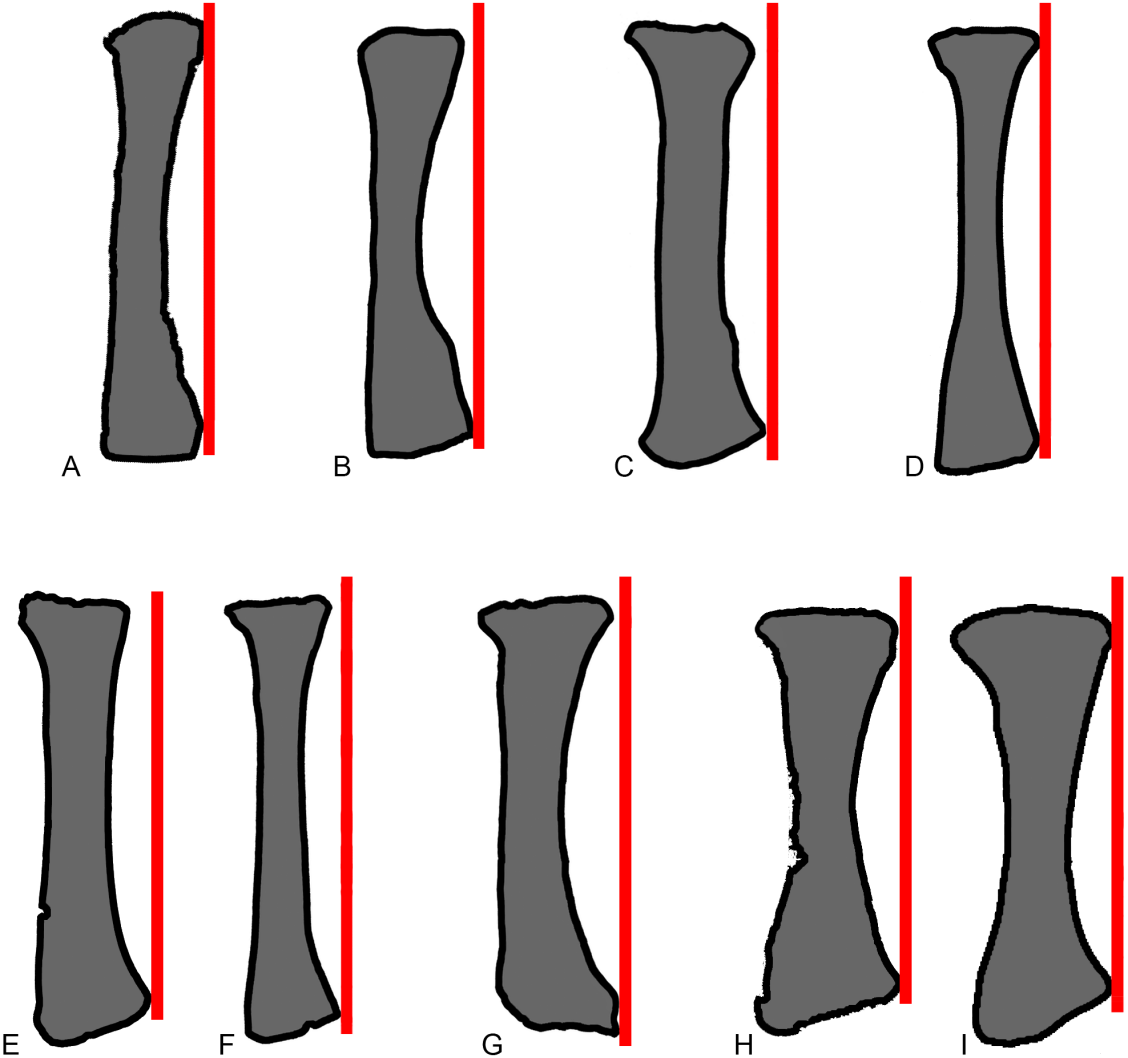
Comparisons of sauropod radii in anterior view. Exemplar profiles of sauropod left radii in anterior view: A, *Mamenchisaurus youngi* (ZDM 0083 [107]); B, *Ferganasaurus* (PIN 3042/1 [101]); C, *Apatosaurus louisae* (CM 3018 [104]); D, *Camarasaurus grandis* (YPM 1901 [100]); E, *Haestasaurus* (NHMUK R1870); F, *Giraffatitan* (MfN MB.R 2181 [87]); G, *Epachthosaurus* (UNPSJB-PV 920, based on a photograph by PDM); H, *Diamantinasaurus* (AAOD 603 [80]); I, *Neuquensaurus* (MLP-CS 1169 [90]). The red lines are drawn parallel to the vertical long-axis of each radial shaft, at a tangent to the lateral tip of the distal end. F-I are right radii that have been reversed in order to facilitate comparison. Profiles not drawn to the same scale.

The radius bows anteriorly in lateral and medial views (Fig 8A and 8B). In anterior view (Fig 7A), the shaft displays the typically sigmoid medial and gently concave lateral margins seen in other sauropod radii (Fig 10). In *Haestasaurus*, this profile is largely produced by expansion of the distal end, which projects somewhat further laterally than the proximal end. In some sauropods (e.g. *Mamenchisaurus* and *Camarasaurus*), the proximal end projects as far laterally as the distal end, but many taxa (e.g. *Ferganasaurus*, *Epachthosaurus*, *Diamantinasaurus)* possess a milder version of the condition seen in *Haestasaurus* (Fig 10). The medial process of the proximal end merges into a vertical edge that extends distally and defines the margin where the anterior and posterior faces of the shaft meet each other. At approximately 20% of radius length from the proximal end, this margin bears a vertical ridge that extends beyond the rest of the medial edge, resulting in a flange-like projection in anterior view (Fig 7A). This vertical ridge corresponds with the insertion point for the combined tendons of the M. biceps brachii and M. brachialis inferior identified on the radius of *Opisthocoelicaudia* by Borsuk-Bialynicka ([27]: fig 8B and 8C) (see ‘New and Revised Characters’ below for further consideration of this feature). In *Haestasaurus*, the posterior surface of the shaft (Fig 7B) lacks the vertically oriented ridge present in *Aragosaurus* (I.G. 484 [111]: fig 9) and several titanosaurs (such as *Rapetosaurus*, FMNH PR 2209 [91]: fig 36C).

At its midlength, the radial shaft has a rounded trapezoidal cross-sectional profile, with strongly convex lateral and medial margins and flattened anterior and posterior faces (the anterior one being narrower transversely than the posterior one). The flat anterior face extends to the distal end, widening transversely and becoming concave between two low vertical ridges. This means that the anterior margin of the distal articular surface is shallowly concave (Fig 8D). This distally located fossa on the anterior surface is absent in other sauropods, including *Apatosaurus ajax* (NSMT-PV 20375 [99]: fig 6), *Camarasaurus grandis* (YPM 1901 [100]: pl. 51, fig 3a), *Diamantinasaurus* (AAOD 603 [80]: fig 12, PU and PDM pers. obs. 2012), *Epachthosaurus* (UNPSJB-PV 920, PU and PDM pers. obs. 2013), *Giraffatitan* (MfN MB.R. 2181 [87]: Beilage A, fig 3), *Patagosaurus* (MACN 932, PU and PDM pers. obs. 2013) and *Tehuelchesaurus* (MPEF-PV 1125 [15]: fig 17, PU and PDM pers. obs. 2013), and is therefore regarded as a potential autapomorphy of *Haestasaurus* (Fig 9I–9P). The flattened posterior surface of the shaft at midlength extends distally and bears a faint but distinct vertical interosseous ridge for attachment to the ulna. Distally, the posteromedial part of the radius produces a prominent bulge, and a smaller rounded projection also occurs posterolaterally (Fig 8D): thus, the radius terminates distally in two posteriorly placed ‘condyles’. Such distal radial condyles occur in several other sauropods and their distribution is evaluated in more detail in ‘New and Revised Characters’ below. In *Haestasaurus*, these condyles create a shallowly concave area on the posterior face, immediately above the distal end, and below the distal termination of the interosseous ridge described above. Another shallow concavity is situated posteromedial to the ridge that defines the medial margin of the anterior fossa, and anterior to the posteromedial condyle just described.

The transverse width of the distal end of the radius is 1.85 times the width of the midshaft (Table 2). Thus, *Haestasaurus* possesses the plesiomorphic state (i.e. values of this ratio less than 2.0) seen in taxa such as *Shunosaurus*, *Omeisaurus*, and *Diplodocus*, rather than the derived condition that occurs in a clade of titanosaurs comprising Nemegtosauridae, *Isisaurus* and Saltasauridae according to Wilson ([10]: character no. 170) (but note that Mannion et al. ([18]: character no. 47) found that values of 2.0 or higher are more widespread in Neosauropoda, occurring in *Apatosaurus*, *Camarasaurus*, *Giraffatitan* and *Tehuelchesaurus*, and the derived state is also seen in *Zby*, ML 368 [112], Table 2).

The distal articular surface of the radius is mildly convex and strongly rugose (Figs 7 and 8D). If the long-axis of the shaft is oriented vertically, the lateral portion of this articular surface slants strongly proximolaterally in anterior view (Fig 7A), at approximately 21° to the horizontal. This proximolateral bevelling of the distal radius was regarded as a synapomorphy of Saltasauridae by Wilson ([10]: character no. 171) (see also [114]): however, the definition and distribution of the derived state requires some clarification. Mannion et al. ([18]: character no. 49) noted that many sauropods have radii in which the distal articular surface has a medial portion that is approximately perpendicular to the shaft long-axis, and a lateral portion that is bevelled. Therefore, for the purposes of measurement and comparison, the bevelling angle is estimated using only the lateral half of the distal articular surface. This angle varies from 0° in basal forms (e.g. *Shunosaurus*, ZDM T5402 [115]; *Mamenchisaurus youngi*, ZDM 0083 [107]: pl. xvii, fig 7), through values of around 20° in *Apatosaurus louisae* (CM 3018 [104]: fig 12A) and *Tehuelchesaurus* (MPEF-PV 1125 [15]: fig 17A, PU and PDM pers. obs. 2013), to more than 25° in many derived titanosaurs such as *Alamosaurus* (USNM 15560 [92]: fig 9A) and *Opisthocoelicaudia* (Z.PAL. MgD-I/48 [27]: fig 8B). Thus, the angle of 21° in *Haestasaurus* is consistent with its placement within basal Neosauropoda or Somphospondyli, rather than Titanosauria (see also [112] for discussion of strong distal radial bevelling in *Turiasaurus* and *Zby*).

#### Skin impression (Fig 11)

**Fig 11:**
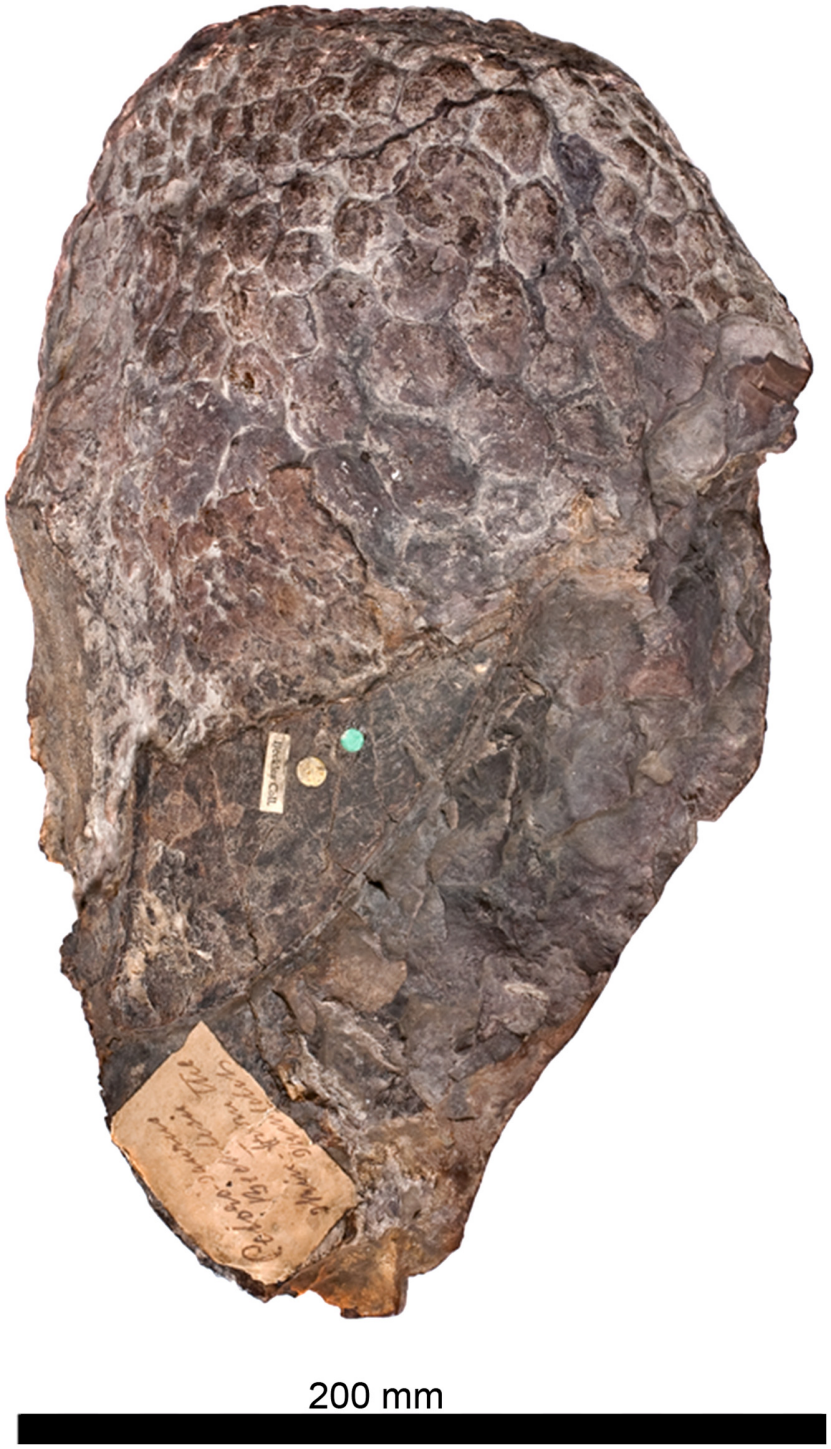
The skin impression of *Haestasaurus becklesii* (NHMUK R1868).

The skin impression (NHMUK R1868) associated with the *Haestasaurus* fore limb was the first specimen of dinosaurian integument to be discovered [22,26]. Upchurch et al. [1] described the *Haestasaurus* skin impression as being composed of ‘ossicles’. If correct, this would represent the stratigraphically oldest evidence for osteoderms in the sauropod fossil record and would also strengthen the suggestion (e.g. [6]) that this taxon belongs to the Titanosauria or even the Lithostrotia. However, as noted by D’Emic et al. [116], the specimen actually represents an impression of the integument, not ossifications.

The *Haestasaurus* skin impression comprises numerous small hexagonal scales that range in size from approximately 10–25 mm in diameter (Fig 11) (see also [117] and [118]: fig 2). These scales tessellate and cover an area of approximately 195 mm x 215 mm. They might have become smaller towards the elbow joint as also occurs in iguanodonts and hadrosaurs according to Steel [66]. If correct, this pattern presumably reflects greater flexibility in the region of the joint [36]. Steel [66] argued that the convex surfaces of these scales (the side that is exposed) actually faced inwards towards the limb bones, based on the observation that their flat surfaces are covered by matrix. However, the dermal remains found with *Saltasaurus* [119], suggest that the exposed convex surfaces might represent the exterior of the skin [36].

The *Haestasaurus* skin impression generally resembles several others assigned to sauropod taxa. For example, a small patch of skin (MWC 6718, a thin carbonaceous film), from the Late Jurassic Mygatt-Moore Quarry, comprises seven hexagonal tubercles and was found in close association with remains of *Apatosaurus excelsus* [26]: fig 2A]. There are several other reports of similar integumentary structures found in association with Morrison Formation sauropods (see review in [26]), and also the basal macronarian *Tehuelchesaurus* (MPEF-PV 1125 [120]: figs 2 and 3). The tubercles of these impressions or films resemble those of *Haestasaurus* in both size and shape. Thus current evidence suggests that asymmetrical hexagonal scales or tubercles that tessellate rather than overlap were probably widespread among at least Neosauropoda.

Czerkas [121] and Foster and Hunt-Foster [26] noted that some sauropod skin impressions or carbonaceous films demonstrate the presence of small (1–2 mm diameter) tubercles on top of the larger hexagonal scales. However, these smaller tubercles cannot be observed in *Haestasaurus*, perhaps because the latter is represented by an impression in relatively coarse grained sediment, or because they were destroyed during preparation of the specimen.

## Phylogenetic Analyses

### Data Sets

In order to determine the phylogenetic relationships of *Haestasaurus*, we have scored this taxon for the data matrix of Carballido and Sander [19] (the ‘CSM’ hereafter), and have revised the scores for “*Pelorosaurus*” *becklesii* in the ‘*Lusotitan* Standard Discrete Matrix’ (LSDM) and ‘*Lusotitan* Continuous and Discrete Matrix’ (LCDM) of Mannion et al. [18]. The CSM has the advantage of a larger and broader taxon sample (71 versus 63 taxa) and more characters (341 versus 279) than the Mannion et al. [18] matrices. However, the latter study might be better placed to assess the relationships of a putative basal titanosauriform, such as *Haestasaurus*, because its taxon and character sampling strategy focussed on this region of the sauropod tree. In addition, whereas the CSM and LSDM treat all character states as discrete, the LCDM allows exploration of the impact of treating quantitative characters as continuous data [18].

“*Pelorosaurus*” *becklesii* was not included in the original CSM: we have therefore scored *Haestasaurus* for this matrix based on the anatomical information presented above. The scores for *Haestasaurus* and the modified CSM (with six new characters, see below) are presented in S1 and S2 Files.

The LSDM and LCDM have been modified in several ways since publication. First, we have incorporated the revised scores for *Diamantinasaurus*, *Wintonotitan*, *Malawisaurus* and *Rapetosaurus* proposed by Poropat et al. [80,81]. Second, most of the scores for “*Pelorosaurus*” *becklesii* employed by Mannion et al. [18] are accepted here. However, we have made the following five changes based on our more detailed examination of *Haestasaurus*:

1. C46. Radius, mediolateral width of proximal to distal end ratio: 1.0 or greater (0); less than 1.0 (1) ([11], modified, quantified, and polarity reversed in [18]. Note that in taxa with a twisted radius, the dimension of the long axis of the distal end is used). In the LCDM this was scored with a value of 0.92 in Mannion et al. [18]. However, after new measurements were taken, the actual value of this ratio is 0.89 (probably slightly higher because of the small portion missing from the medial process of the proximal end). Here, therefore, we have adjusted this score for *Haestasaurus* to be 0.89–0.92 in the LCDM, in order to reflect this slight uncertainty.
2. C47. Radius, distal end mediolateral width to midshaft mediolateral width ratio: less than 2.0 (0); 2.0 or greater (1) ([10,122], modified by [18]. Note that in taxa with a twisted radius, the dimension of the long axis of the distal end is used). The new measurements for the *Haestasaurus* radius mean that its score in the LCDM has been adjusted from 1.71 in [18] to 1.852 here.
3. C48. Radius, distal end mediolateral to anteroposterior width ratio: 1.5 or greater (0); less than 1.5 (1) ([9], quantified and polarity reversed by [18]). The new measurements for the *Haestasaurus* radius mean that its score in the LCDM has been adjusted from 1.405 in [18] to 1.527 here. This also means that the score for *Haestasaurus* in the LSDM has been changed from 1 to 0.
4. C228. Humerus, distal-most part of the posterior surface (supracondylar fossa) is: flat or shallowly concave (0); deeply concave between prominent lateral and medial vertical condylar ridges [1,18]. *Haestasaurus* was scored with state 1 by Mannion et al. [18]. However, although the ridges that define the anconeal (= supracondylar) fossa are fairly prominent in this taxon, the actual depth of the fossa is shallow (see Table 2 and ‘New and Revised Characters’ below’). We have therefore changed the score for this character to state 0 in both the LSDM and LCDM.
5. C233. Ulnar olecranon process, development: absent or only rudimentary, i.e. projecting just above the proximal articulation (0); prominent, projecting well above proximal articulation (1) ([9,64], polarity reversed by [18]). *Haestasaurus* was scored with state 0 by Mannion et al. [18]: however, this was an error and has been corrected to state 1 in the LSDM and LCDM here.

The revised character scores for the LSDM and LCDM and the complete data matrices (with six new characters, see below) are presented in S1, S3 and S4 Files.

### New and Revised Characters

The detailed description and comparison of *Haestasaurus* has highlighted some problems with existing fore limb characters, and has also identified several new and potentially phylogenetically informative features. Below, we briefly discuss three revised characters (“RC”) and six new characters (‘NC’). The latter have been added to the CSM, LSDM and LCDM. Complete scores for all taxa for NC1-6 are presented in Tables B and C in S1 File. These new characters have been added at the end of each character set, so that NC1-6 form characters 342–347 in the CSM and characters C280–285 in the LSDM and LCDM, respectively (this has been done so that the original character numbers used by Carballido and Sander [19] and Mannion et al. [18] remain unaltered, and therefore correspond with those cited in the text here).

#### Character revisions and comments

RC1. 256. Humerus, RI (sensu [31]): gracile (less than 0.27) (0); medium (0.28–0.32) (1); robust (more than 0.33) (2) [123]. Table 2 indicates that the RI can reach particularly low values (0.21) in brachiosaurids such as *Cedarosaurus* and *Giraffatitan*, reflecting the relative elongation of the humerus in such taxa. At present, this potential synapomorphy of Brachiosauridae, or a clade within this family, is not captured by the state definitions. In future analyses, it might be appropriate to introduce an additional state, or simply treat the RI values as continuous data.

**Fig 12:**
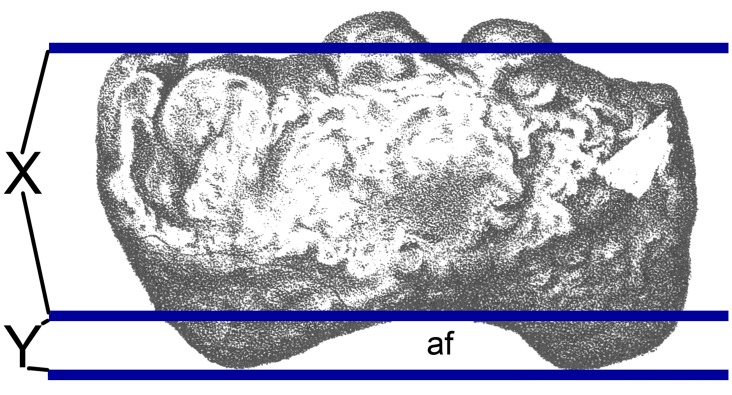
Definition of anconeal fossa depth ratio. The distal end of the humerus of *Camarasaurus grandis* (YPM 1901, modified from Ostrom and McIntosh [100]), showing the definition of the ratio used to estimate anconeal (= supracondylar) fossa depth. Ratio = Y/X (see Table 2). Note that X excludes the anterodistal processes if present: this is because these processes are often absent or highly reduced in titanosaurs, and their inclusion in X would mean that the fossa depth ratio would be estimated in an inconsistent manner across Sauropoda. Abbreviation: af, anconeal fossa.

RC2. C228. Humerus, distalmost part of the posterior surface (supracondylar fossa) is: flat or shallowly concave (0); deeply concave between prominent lateral and medial vertical condylar ridges (1) [1,18]. This fossa varies in depth among sauropod taxa (see Fig 4), and a deep fossa, bounded by acute lateral and medial ridges, has generally been regarded as a derived state that characterises most somphospondylans (e.g. [1,18]): however, the depth of this fossa has not been quantified. Here, we have devised a simple ratio to capture fossa depth (Fig 12) and use this to redefine this character in quantitative terms as follows: ‘Humerus—anconeal fossa /depth: shallow (depth of fossa divided by anteroposterior width of humeral distal end) is less than 0.4 (0); deep, fossa depth ratio is 0.4 or higher (1) (N.B. the anteroposterior width of the distal humerus [‘X’ in Fig 12] excludes the depth of the anconeal fossa itself and the contribution made by the lateral and medial anterodistal processes).’ The boundary between states is based on the data in Table 2, and it appears that most titanosauriforms have a fossa depth ratio of at least 0.4 (e.g. *Elaltitan*, *Epachthosaurus*, *Giraffatitan*, *Malawisaurus* and *Rapetosaurus*), whereas non-neosauropod eusauropods, diplodocoids and basal macronarians typically have values of 0.2 or lower. Based on this criterion, *Haestasaurus* has state 0, and its score has therefore been adjusted here (from its previous score of state 1) in the LSDM and LCDM. One other aspect of this feature requires further investigation. *Haestasaurus* and many titanosauriforms appear to have more prominent lateral and medial ridges that define the margins of the anconeal fossa. The medial ridge is more prominent than the lateral one, and rather than being transversely rounded (as in more basal taxa) those of titanosauriforms have more acute apices. It seems that ridge shape and fossa depth are not completely correlated: future phylogenetic analyses might need to treat these two features as independent characters. However, some caution is required because the apparent independence of ridge shape/prominence and anconeal fossa depth might be an artefact produced by post-mortem crushing of some humeri. Furthermore, it is difficult to quantify the difference between a low rounded ridge and a more prominent and acute one.

**Fig 13:**
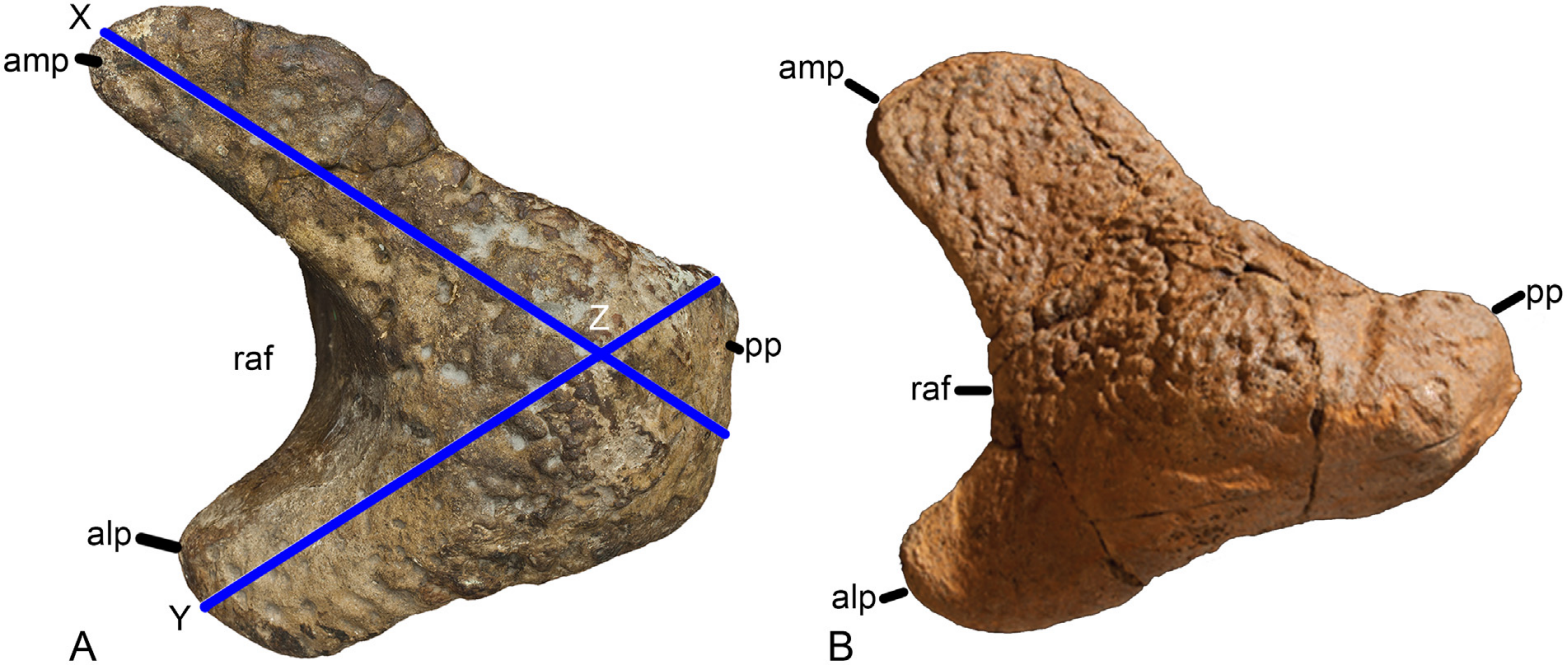
Sauropod ulnae in proximal end view. A, left ulna of *Haestasaurus becklesii* (NHMUK R1870); B, right ulna (reversed so that it looks like a left) of *Diamantinasaurus matildae* (AAOD 603 [from 80]). Abbreviations: alp, anterolateral process; amp, anteromedial process; pp, posterior process; raf, fossa for reception of the proximal end of the radius. The blue lines in A mark the long-axes of the anteromedial and anterolateral processes; X, Y and Z mark the tip of the anteromedial process, the tip of the anterolateral process and the intersection of the process long-axes respectively. The anteromedial:anterolateral process length ratio (‘Uppl’ in Table 2) can thus be defined as X-Z/Y-Z.

RC3. C51. Ulna, ratio of maximum mediolateral width of proximal end (equivalent to anteromedial arm) to maximum anteroposterior width of proximal end (equivalent to anterolateral arm): less than 2.0 (0); 2.0 or greater (1) ([10], modified and quantified by [18]; see also [17]). There are two problems associated with the character state definitions used by Mannion et al. [18]. First, the use of maximum anteroposterior width of the proximal ulna to represent the length of the anterolateral process means that the posterior process usually contributes to this measurement. However, as noted in NC2 below, the posterior process itself varies in relative prominence among sauropods. Second, the process length ratio can alter markedly because of changes in the orientations of the anterolateral and anteromedial processes, rather than because of changes in their relative lengths. In particular, anteromedial processes can project more medially or more anteriorly; such shifts in orientation affect the mediolateral width of the proximal ulna, even if the relative length of the process has not changed. In order to avoid possible inconsistencies in measurement and comparison, we propose a standardised method for defining the lengths of these processes (as illustrated in Fig 13) that excludes the size of the posterior process. Essentially, the long-axes of the anteromedial and anterolateral processes are extrapolated posteriorly so that they intersect close to the position of the olecranon. Process length is then measured from this intersection to the tip of each process. The resulting process length ratios are shown in Table 2, where they can be compared with those obtained by Mannion et al. [18]. We have utilised these revised values here. As a result, the “Cloverly titanosauriform’ has been scored with state 0 (rather than 1) for C51 in the LSDM. Moreover, many of the scores for C51 in the LCDM have been modified (see Table A in S1 File for details).

We noted above (see ‘Description’) that D’Emic [17] supported inclusion of *Haestasaurus* within Titanosauriformes on the basis that the anteromedial process of the ulna is longer than the anterolateral one. Here, the data in Table 2 do not support this view, irrespective of whether the process length ratio is based on the state definition used by Mannion et al. [18] or the revised version proposed here. Most sauropods have an anteromedial process that is longer than the anterolateral one, and especially long anteromedial processes (i.e. process length ratio of 2.0 or higher) are restricted to a minority of titanosauriforms (e.g. *Cedarosaurus*, *Epachthosaurus* and *Venenosaurus*).

#### New characters

NC1. Humerus—attachment for M. scapulohumeralis anterior: weakly developed and not visible in anterior view (0); forms a distinct lateral bulge (that interrupts the line of the lateral humeral margin in anterior view) located posterolateral to the deltopectoral crest (1) ([27]; fig 7C and 7D). State 0 is present in most sauropods. State 1 occurs in several titanosaurs (e.g. *Malawisaurus*, MAL 221 [95]: fig 20) and is most prominent in *Epachthosaurus* (UNPSJB-PV 920 [89]: fig 9, PU and PDM pers. obs. 2013) and derived lithostrotians (e.g. *Alamosaurus*, USNM 15560 [92]: fig 5; *Bonatitan*, MACN-PV RN 821 [124]: fig 8; *Neuquensaurus*, MLP-CS 1050 [90]: fig 3; *Saltasaurus*, PVL4017-67 [96]: fig 31).

NC2. Ulna—posterior process of proximal end: is weakly developed, so that the proximal profile of the ulna is ‘V’-shaped (formed by the anteromedial and anterolateral processes) (0); is strongly developed, so that the proximal profile of the ulna is ‘T’- or ‘Y’-shaped, and there is a deep fossa between the anteromedial and posterior processes rivalling the radial fossa in depth (1) (Fig 13). The plesiomorphic state (Fig 13A) occurs in most sauropods, including *Apatosaurus ajax* (NSMT-PV 20375 [99]: fig 6C), *Camarasaurus grandis* (YPM 1901 [100]: pl. 53, fig 3a), *Epachthosaurus* (UNPSJB-PV 920, PU and PDM pers. obs. 2013), *Ferganasaurus* (PIN N 3042/1 [101]: fig 7D), *Giraffatitan* (MfN MB.R 2181 [87]: Beilage A, fig 3c), *Mamenchisaurus youngi* (ZDM 0083 [107]: fig 36C), *Omeisaurus tianfuensis* (ZDM T5704 [108]: fig 46B) and *Tehuelchesaurus* (MPEF-PV 1125 [15]: fig 16B). In contrast, the derived state is seen in several somphospondylans, such as *Diamantinasaurus* (Fig 13B), *Magyarosaurus* (NHMUK 3859, PU and PDM pers. obs. 2014), *Rapetosaurus* (FMNH PR 2209 [91]: fig 37D), *Saltasaurus* (PVL 4017–74, PU and PDM pers. obs. 2013) and *Wintonotitan* (QM 7292 [81]: fig 10E). In these somphospondylans, it can sometimes be difficult to correctly determine whether an ulna is from the right or left side because the deep fossa created between the anteromedial and posterior processes can be mistaken for the radial fossa, and the relatively short anterolateral process can be confused with the enlarged posterior process (especially when the proximal profile is more ‘T’-shaped, as in *Rapetosaurus*). However, the anterior surface of the ulna can be identified by the well-developed interosseous ridge for attachment of the radius, which lies distal to the true radial fossa.

**Fig 14:**
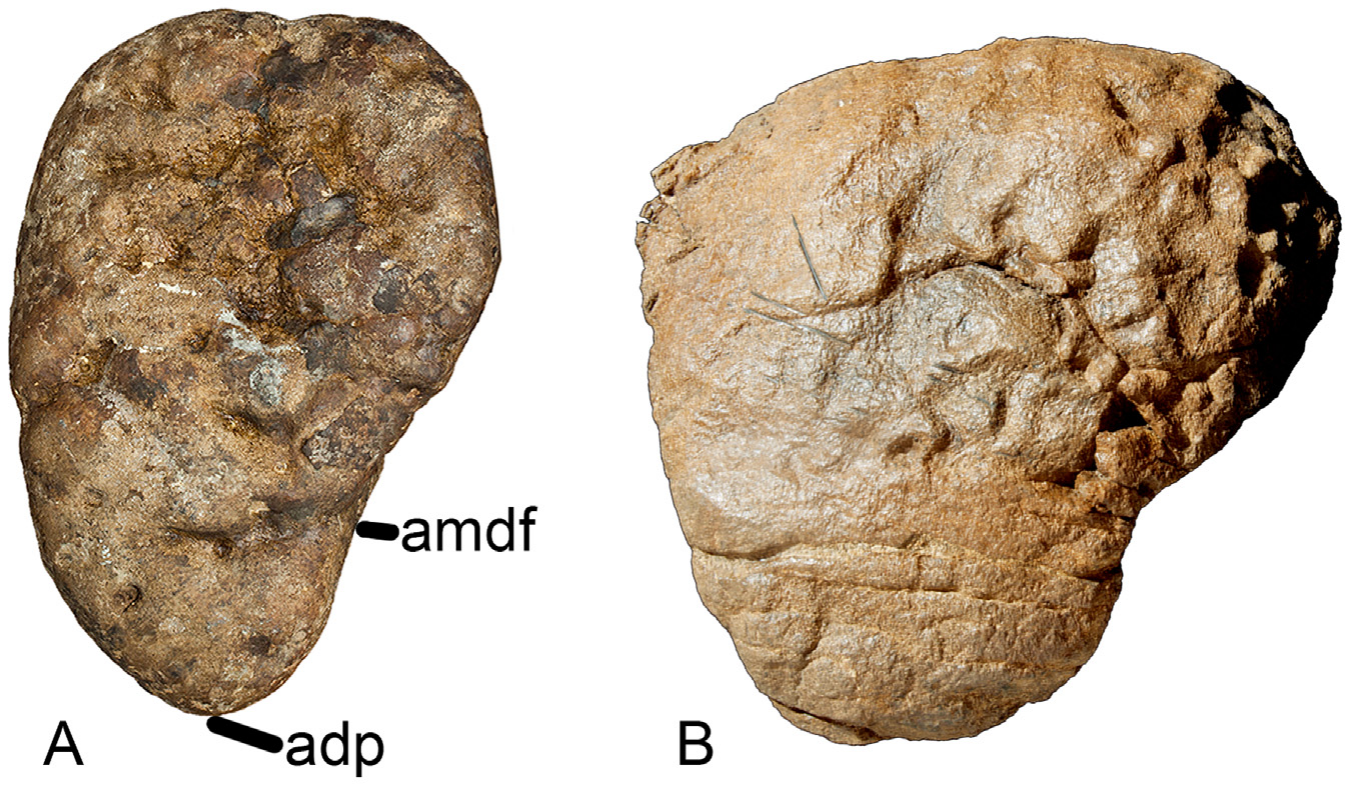
Sauropod ulnae in distal end view (anterior towards bottom). A, left ulna of *Haestasaurus becklesii* (NHMUK R1870); B, right ulna (reversed so that it looks like a left) of *Diamantinasaurus matildae* (AAOD 603 [photograph courtesy of S. Poropat). Abbreviations: adp, anterior distal process; amdf, anteromedially facing fossa immediately above the distal end.

**Fig 15:**
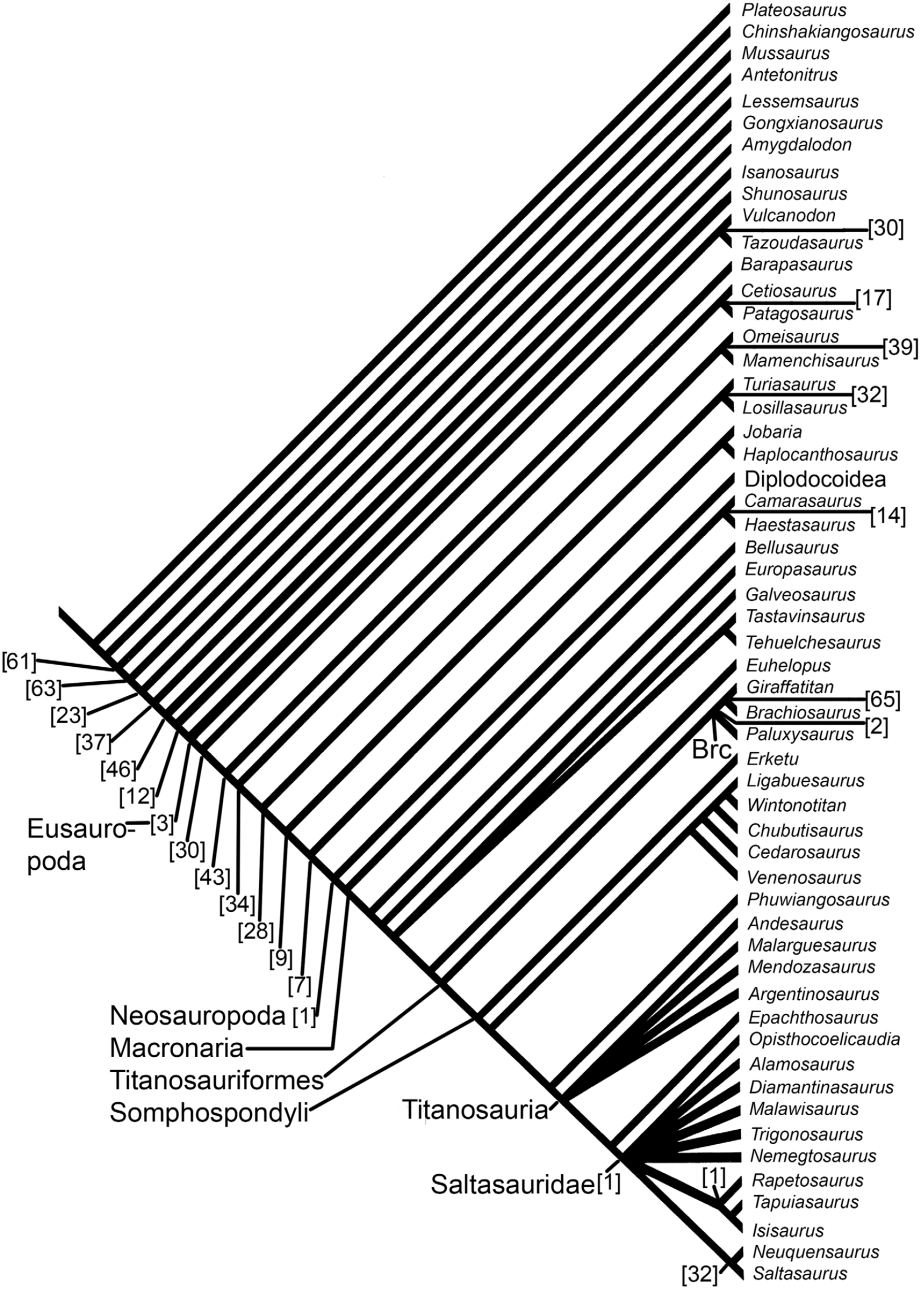
Strict consensus tree (CSM). A strict consensus tree based on the 28 most parsimonious trees generated by analysis of the Carballido and Sander [19] data matrix with the addition of *Haestasaurus* and six new characters (*Tendaguria* excluded a priori). GC values (multiplied by 100) are shown in square brackets for all nodes where these values are greater than 0. The monophyletic Diplodocoidea has been collapsed to a single branch in order to reduce figure size. Abbreviation: Brc, Brachiosauridae. See main text for details.

NC3. Ulna—shape of the distal end: comma-shaped, with tapering curved anterior process associated with an anteromedial fossa for reception of the radius (0); elliptical or subtriangular in outline, with the anteromedial fossa reduced or absent (1) (Fig 14). The comma-shaped distal end, and associated anteromedial fossa for reception of the distal radius, is widespread among neosauropods, occurring in taxa such as *Apatosaurus excelsus* (YPM4633 [100]: pl. 52, fig 4), *Camarasaurus grandis* (YPM1901 [100]: pl. 53, fig 1a), *Giraffatitan* (MfN MB.R. 2181 [87]: Beilage A, fig 2d, PDM pers. obs. 2014) and *Tehuelchesaurus* (MPEF-PV 1125, PU and PDM pers. obs. 2013), as well as some basal eusauropods (e.g. *Omeisaurus tianfuensis*, ZDM T5704 [108]: fig 46C), but is absent in many other basal eusauropods (e.g. *Patagosaurus*, MACN 932, PU and PDM pers. obs. 2013). Derived titanosaurs such as *Diamantinasaurus* (AAOD 603 [80]: fig 15H), *Epachthosaurus* (UNPSJB-PV 920, PU and PDM pers. obs. 2013) and *Saltasaurus* (PVL 4017–74, PU and PDM pers. obs. 2013) also lack the ‘comma’-shaped distal ulna, and the reduced fossa for the reception of the distal radius might have faced more anteriorly rather than anteromedially. In at least some titanosaurs, such as *Diamantinasaurus* (Fig 14B), the distal ulna is heart-shaped/subtriangular rather than elliptical in outline. At present, this condition is scored as state 1 because it is different from the comma-shape scored as state 0. In future analyses, it might be preferable to score the heart-shaped distal end as a separate state from the elliptical one: however, we have not done this here because the distribution of these morphologies requires further investigation via firsthand observation of additional titanosaurian taxa.

NC4. Radius—profile of proximal end: ‘D’-shaped or elliptical (0); oval or subtriangular, with marked tapering towards the medial process (1) (Fig 9). The derived state occurs in many titanosauriforms, including *Diamantinasaurus*, *Epachthosaurus*, *Giraffatitan* and *Neuquensaurus* (Fig 9E–9H).

NC5. Radius—ridge or flange on medial margin, near proximal end, for attachment of the M. biceps brachii and M. brachialis inferior: absent or very weakly developed (0); present, projecting beyond the medial margin of the main radial shaft (1) (Fig 7). The derived state is a low, vertically elongated bulge or ridge on the medial margin, located at approximately 20% of the element length from the proximal end. This structure was first noted by Borsuk-Bialynicka [27], who proposed that it represents the attachment of the M. biceps brachii and M. brachialis inferior in *Opisthocoelicaudia*. Subsequently, the derived state has also been observed in *Diamantinasaurus* (AAOD 603, PU and PDM pers. obs. 2012), *Epachthosaurus* (UNPSJB-PV 920, PU and PDM pers. obs. 2013), *Giraffatitan* (MfN MB.R. 2181, PDM pers. obs. 2014), *Haestasaurus* (Fig 7) and *Huabeisaurus* (HBV-20001 [113], PU and PDM pers. obs. 2012). Given that a projection in this region is absent in more basal sauropods (e.g. *Patagosaurus* [MACN 932, PU and PDM [pers. obs. 2013], *Tehuelchesaurus* [MPEF-PV 1125, PU and PDM pers. obs. 2013]), its presence potentially represents a derived state that characterises Titanosauriformes.

NC6. Radius—posterior margin of distal end: lacks condyles and fossa (0); forms two low rounded condyles (posteromedial and posterolateral) with a shallow fossa between them (1) (based on D’Emic [125]) (Fig 9). The presence of distal radial projections or condyles was noted in “*Astrodon johnstoni*” by Carpenter and Tidwell [97]: p.100) and proposed as an autapomorphy of that taxon. However, D’Emic [125] argued that similar ‘condyles’ occur in other sauropods, such as *Camarasaurus grandis* (YPM 1901 [100]: pl. 51, fig 3a). We have also noted the presence of such condyles in *Epachthosaurus* (UNPSJB-PV 920, PU and PDM pers. obs. 2013), *Giraffatitan* (MfN MB.R. 2181 [87]: Beilage A, fig 3d) and *Tehuelchesaurus* (MPEF-PV 1125 [15]: fig 17, PU and PDM pers. obs. 2013). These condyles are absent in basal sauropods such as *Omeisaurus tianfuensis* (ZDM T5701 [108]: pl. xiv, fig 2c) and *Tazoudasaurus* (CPSGM To2-112 [126]: fig 23A,B), and several titanosauriforms (e.g. *Cedarosaurus*, DMNH 39045 [94]: fig 8B; *Diamantinasaurus*, AAOD 603, PU and PDM pers. obs. 2012; *Malawisaurus* [95]; *Opisthocoelicaudia*, Z.PAL MgD-I/48 [27]: pl. 11, fig 3; *Saltasaurus*, PVL 4017–78, PU and PDM pers. obs. 2013).

### Analytical Methods

The CSM, LSDM and LCDM were analysed using TNT vs. 1.1 [127]. The multistate characters treated as ordered by Carballido and Sander [19] and Mannion et al. [18] were also treated as such in all analyses here. For all data sets, the New Technology search was first applied (including Drift, Sectorial Searches and Tree Fusing) with the consensus being stabilised 10 times. The most parsimonious trees (MPTs) produced by these searches were then used as the starting trees for two consecutive rounds of Traditional (heuristic) search using TBR. The LCDM was analysed in the same way, but with application of Implied Weights (with a k value of 3) [18,128].

The robustness of the resulting relationships was evaluated by generating relative bootstrap frequencies (known as GC values) using symmetric resampling in TNT [127,129]. In each case, 5000 bootstrap replicates were generated using the Traditional Search with TBR.

The character states that support the relationships of *Haestasaurus* in the various MPTs have been examined using character mapping in Mesquite vs. 2.75 [130].

### Results

#### Carballido and Sander [19]—CSM

Analysis of the CSM in TNT yielded 99,999 MPTs of length 1126 steps, before the available memory became full. The ‘pruned trees’ option was then applied to these MPTs. This indicated that *Tendaguria* was the most unstable taxon. *Tendaguria* was therefore excluded a priori, and the analyses were run again. This produced 28 MPTs of length 1123 steps. The strict consensus tree of these 28 MPTs is shown in Fig 15, along with GC values. *Haestasaurus* forms a clade with *Camarasaurus*, and this pair is the sister-taxon to all other macronarians.

#### Mannion et al. [18]—LSDM

**Fig 16:**
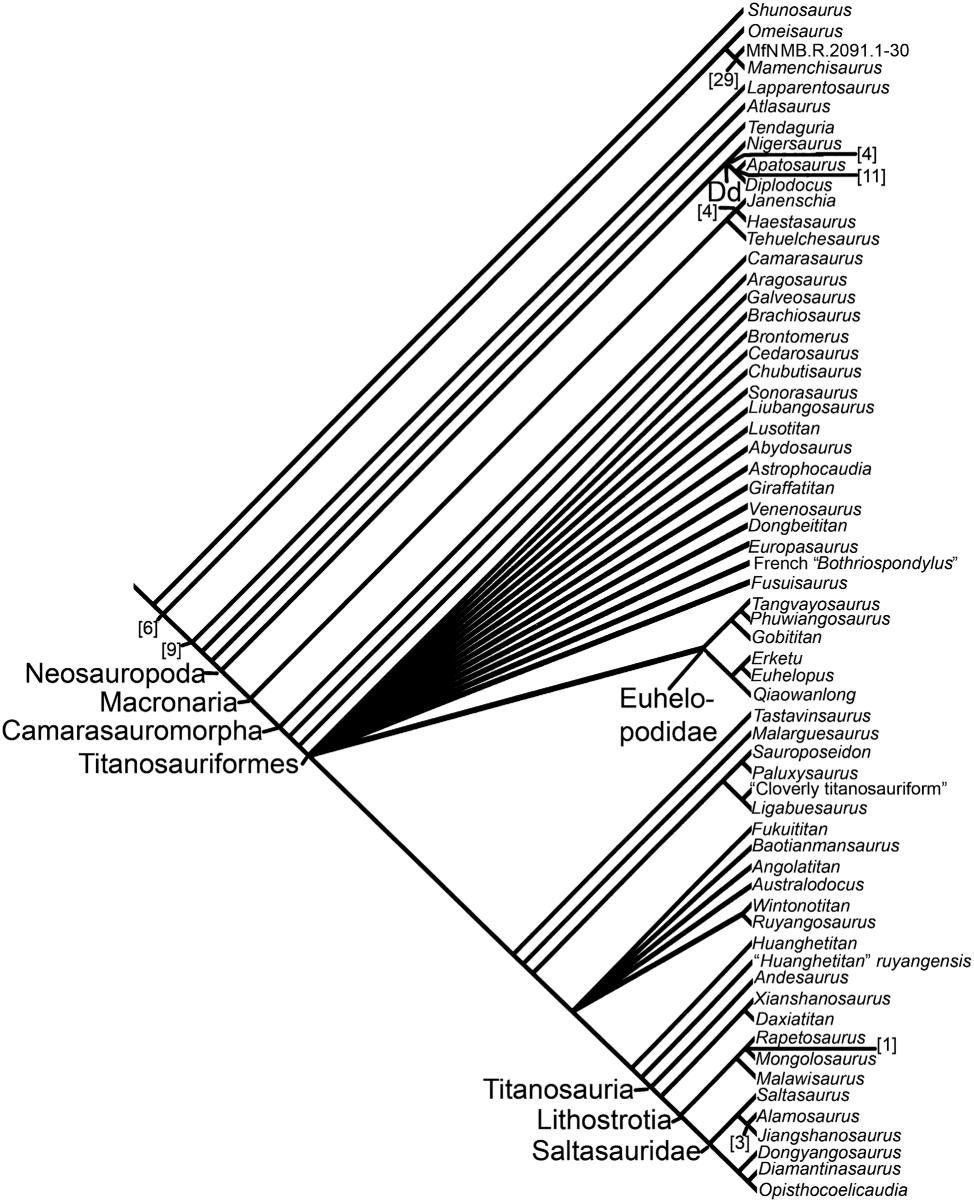
Strict consensus tree (LSDM). A strict consensus tree based on the 1778 most parsimonious trees generated by analysis of the Mannion et al. [18] LSDM with the revised scores for *Haestasaurus* and the addition of six new characters. GC values (multiplied by 100) are shown in square brackets for all nodes where these values are greater than 0. Abbreviation: Dd, Diplodocoidea. See main text for details.

Analysis of the LSDM in TNT yielded 1778 MPTs of length 1089 steps. The strict consensus of these MPTs is shown in Fig 16, along with GC values. This strict consensus indicates that *Haestasaurus* forms a clade with *Tehuelchesaurus* and *Janenschia*, with this clade being the sister-taxon to Camarasauromorpha.

#### Mannion et al. [18]—LCDM

**Fig 17:**
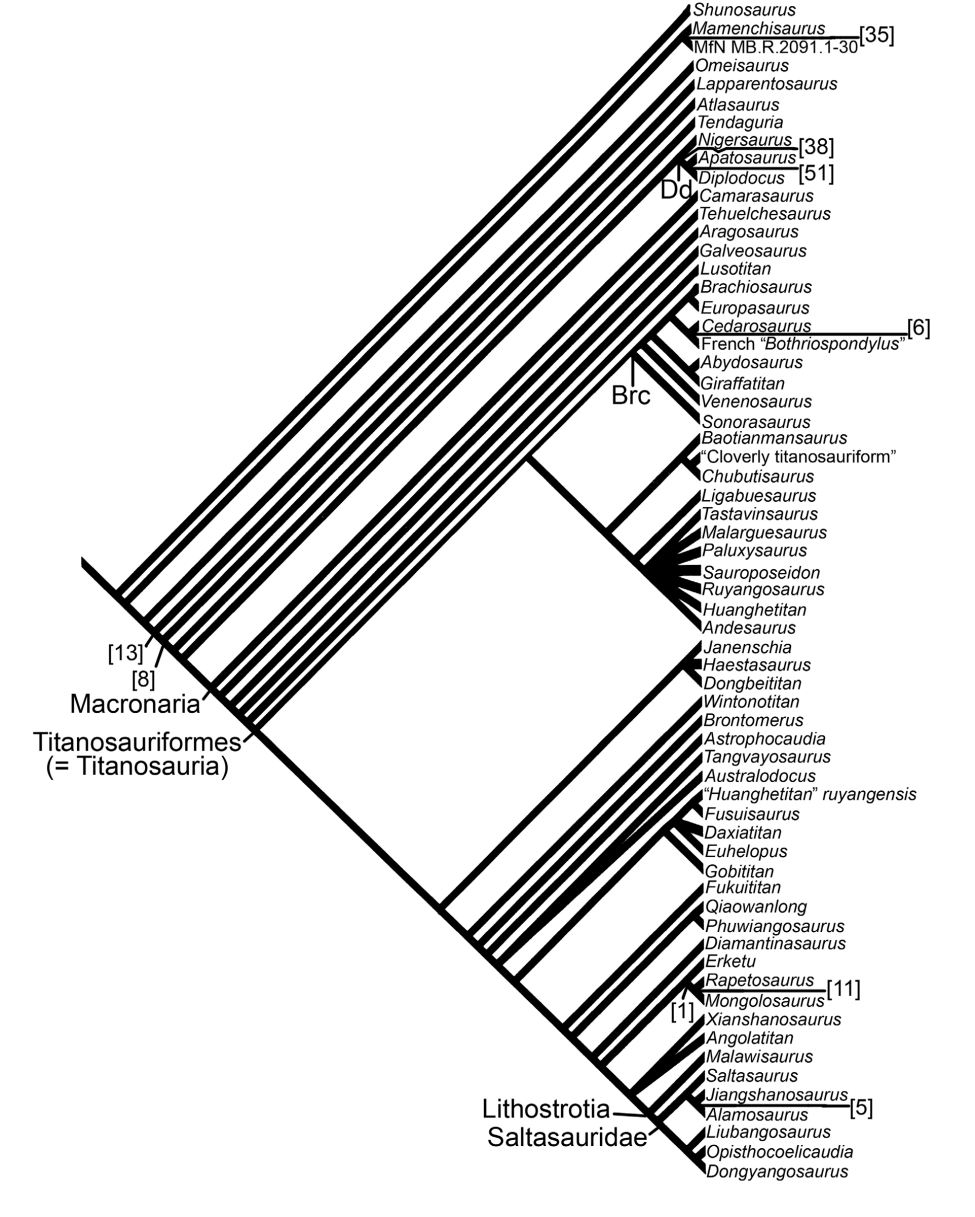
Strict consensus tree (LCDM). A strict consensus tree based on the 17 most parsimonious trees generated by analysis of the Mannion et al. [18] LCDM with the revised scores for *Haestasaurus* and the addition of six new characters. GC values (multiplied by 100) are shown in square brackets for all nodes where these values are greater than 0. Abbreviations: Brc, Brachiosauridae; Dd, Diplodocoidea. N.B. the tree topology shown here means that the clades defined by *Brachiosaurus*+*Saltasaurus* (Titanosauriformes) and *Andesaurus*+*Saltasaurus* (Titanosauria) are identical. See main text for details.

Analysis of the LCDM in TNT yielded 17 MPTs of length 105.53919 steps (N.B. the non-integer tree length, and a tree length that is shorter than the total number of characters, result from the use of continuous data and the application of implied weights [18]). The strict consensus tree of the 17 MPTs is shown in Fig 17, along with GC values. In this strict consensus tree, *Haestasaurus* is placed in a clade with *Janenschia* and *Dongbeititan*. This clade is more closely related to *Saltasaurus* than is *Andesaurus*, so technically *Haestasaurus* is placed within Titanosauria. However, this analysis has produced an unusual result in which *Andesaurus*, and several other taxa that are normally considered to be somphospondylans (e.g. *Chubutisaurus*), form a clade that is the sister-taxon to a clade that comprises traditional brachiosaurids.

## Discussion

### *Haestasaurus* and *Pelorosaurus*

As explained in the ‘Historical Background’ section above, *Haestasaurus* has been known as “*Pelorosaurus*” *becklesii* since its discovery and announcement [22]. In order to justify the new generic name, it is not only necessary to identify autapomorphies of *H*. *becklesii*, but also to demonstrate that this taxon is distinct from *Pelorosaurus conybeari*. The latter taxon is known from the syntype specimens comprising a right humerus (NHMUK 28626), four anterior caudal vertebrae (NHMUK R2544–2547) and three chevrons (NHMUK R2548–2550), as well as some referred middle and distal caudals [36]. Thus, at present, only the humerus allows comparisons between *Pelorosaurus conybeari* and *Haestasaurus becklesii*.

**Fig 18:**
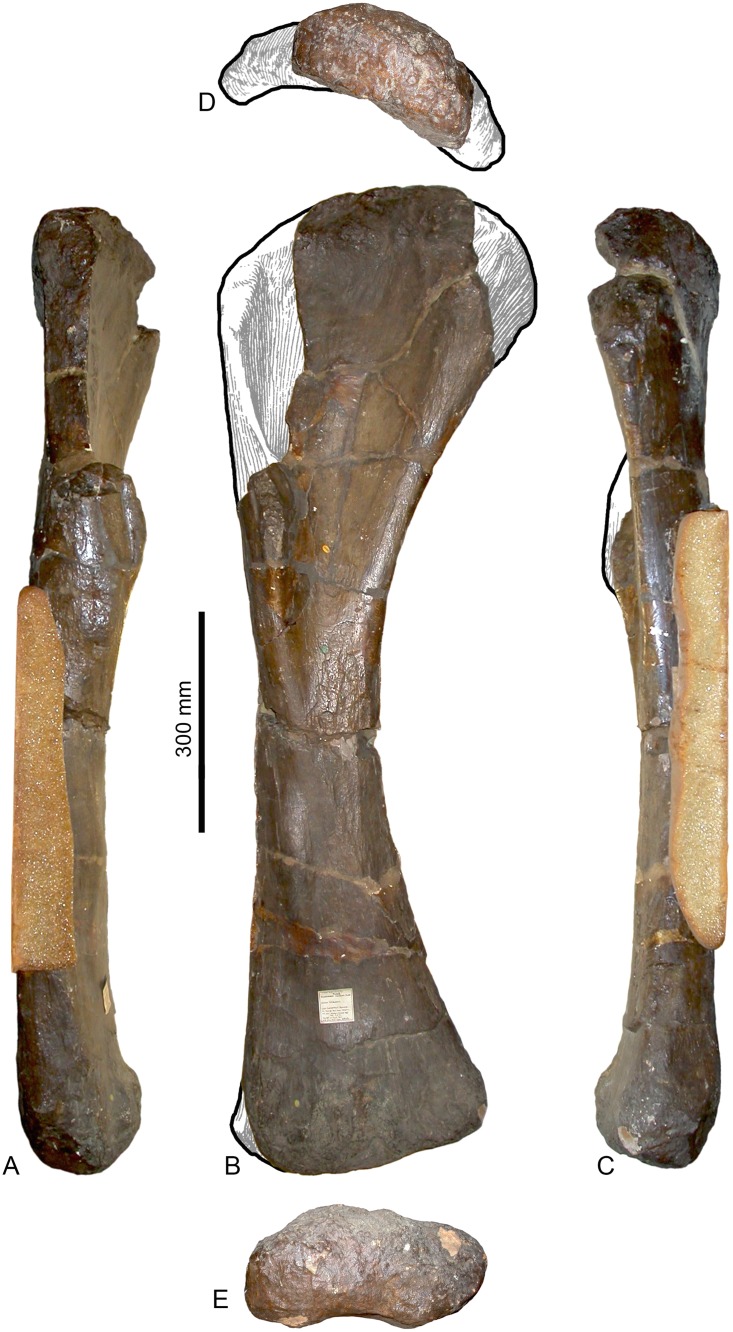
Right humerus of *Pelorosaurus conybeari* (NHMUK 28626). A, lateral; B, anterior; C, medial; D, proximal; E, distal views. Missing portions have been reconstructed using the humerus of *Giraffatitan* as a guide.

Descriptions, figures and photographs of *Pelorosaurus conybeari* have been presented in several recent studies ([28,34] and [36]: fig 7), and these will not be repeated here. However, we have provided a new figure of the right humerus of *Pelorosaurus conybeari* (Fig 18) in order to facilitate comparisons with *Haestasaurus*. The 1320 mm long *Pelorosaurus conybeari* humerus is incomplete, lacking both the lateral and medial margins of the proximal end (Fig 18). Nevertheless, it is clear that the humeri of *Pelorosaurus* and *Haestasaurus* are very different in terms of their robustness. The RI [sensu 31] for *Haestasaurus* is 0.33, whereas that for *P*. *conybeari* is estimated to be approximately 0.23 (Table 2). Thus, whereas *Haestasaurus* would be scored with state 2 for Carballido and Sander’s [19] character no. 256 dealing with humeral RI values, *Pelorosaurus* would be scored with state 0. Although the proximal end of the *Pelorosaurus* humerus is badly damaged, enough remains to indicate that the proximal articular surface was convex transversely and curved distally towards the proximolateral corner (Fig 18B): this suggests that the proximolateral corner was rounded rather than ‘square’, and that the lateral portion of the proximal articular surface lay below the level of the humeral head. If correct, then *Pelorosaurus* retains the plesiomorphic state, whereas *Haestasaurus* possesses the derived state that occurs in most somphospondylans (see ‘Description’; above). The lateral margin of the *Pelorosaurus* humerus is straight proximodistally, whereas *Haestasaurus* retains the plesiomorphic concave profile. Finally, although both genera would be scored with state 0 for the character pertaining to anconeal fossa depth (see ‘RC2’ above), this fossa is more prominent and bounded by well-developed lateral and medial ridges in *Haestasaurus*, whereas in *Pelorosaurus* it is very shallow (Table 2; compare Figs 3D and 18E). Thus, *Pelorosaurus* and *Haestasaurus* differ in at least four humeral characters that have been employed in recent phylogenetic analyses (e.g. [17–19]).

The two humeral autapomorphies of *Haestasaurus* both pertain to the distal end. Unfortunately, this region is damaged and worn in *Pelorosaurus*. Thus, it is not clear whether the latter taxon possessed one or two small ridges between the lateral and medial anterodistal processes (indeed, these processes are themselves not well preserved). However, enough of the distal end is preserved to suggest that *Pelorosaurus* lacked the prominent anterior entepicondylar process that characterises *Haestasaurus*.

In short, the limited data available at present indicate that *Haestasaurus becklesii* and *Pelorosaurus conybeari* differ in at least five character states pertaining to the humerus alone. This indicates that they are distinct taxa and should be treated as separate genera.

### Phylogenetic Affinities of *Haestasaurus*

As noted in the ‘Historical Background’ section above, most recent studies have concluded that *Haestasaurus becklesii* is a titanosauriform, but there has been disagreement over whether it is a basal member of this clade or a more derived titanosaur (e.g. [1,6,17,18,36]). The phylogenetic analyses presented here all confirm that *Haestasaurus* is at least a macronarian. This is supported by the presence of derived states such as the square proximolateral corner of the humerus and the associated straight lateral portion of the proximal end that lies level with the humeral head (character nos. 260 and C223 in the CSM and LSDM/LCDM respectively). Moreover, all analyses, both here and by other workers, agree that *Haestasaurus* is not a brachiosaurid. None of the putative brachiosaurid synapomorphies discussed by D’Emic [17] and Mannion et al. [18] can be observed in *Haestasaurus*, and the humerus of the latter taxon clearly lacks the rather slender and elongated morphology that characterises most brachiosaurids (e.g. see [131]).

The current phylogenetic analyses tend to support one of two placements of *Haestasaurus*: (1) closely related to taxa such as *Camarasaurus*, *Janenschia* and *Tehuelchesaurus* at the base of Macronaria (CSM and LSDM); or (2) positioned as a basal member of Titanosauria (LCDM). It seems likely, therefore, that some of the fore limb characters tend to exclude *Haestasaurus* from Titanosauriformes, whereas others represent synapomorphies that unite this English taxon with titanosaurs. However, given the large amounts of missing data and homoplasy, most of these characters form weak synapomorphies with low individual consistency and retention indices. In particular, *Haestasaurus* is very incomplete: it can be scored for just 21 (6.1%) and 31 (10.9%) of the characters for the modified CSM and LSDM/LCDM respectively. Moreover, homoplasy seems to be rife in fore limb characters for sauropods. For example, consider character nos. 264 and C50 in the CSM and LSDM/LCDM respectively, in which the derived state is a robust ulna. Based on the results of Mannion et al. [18], it appears that robust ulnae have evolved at least four times independently in the following taxa: (1) a potentially monophyletic assemblage of Jurassic Chinese sauropods that includes *Bellusaurus*, *Hudiesaurus* and some species of *Mamenchisaurus*; (2) the diplodocoid *Apatosaurus*; (3) basal macronarians such as *Janenschia* and *Tehuelchesaurus*; and (4) derived titanosaurs such as *Opisthocoelicaudia* and *Saltasaurus*. Finally, many of the available fore limb characters relate to the proportions of elements. The phylogenetic significance of such quantitative characters is much more strongly influenced by the methodological strategy adopted by a systematist than would be the case for a simple qualitative character (e.g. an absence/presence character). Further insights into these issues, and the evidence supporting the alternative relationships of *Haestasaurus*, can be obtained by detailed character mapping.

In order to determine how the support for a given relationship of *Haestasaurus* varies with data set and tree topology, character mapping was carried out in Mesquite 2.75 [130]. Character state distributions were explored by mapping each fore limb character onto the associated strict consensus trees shown in Figs 15–17. Mapping was also carried out using exemplar MPTs and agreement subtrees. These investigations indicate that *Haestasaurus* tends to be excluded from Titanosauriformes (or less inclusive clades such as Somphospondyli, Titanosauria etc.) because it possesses the following 14 character states:

1. The deltopectoral crest does not expand medially across the anterior face of the humerus (character nos. 254 and C225 in the CSM and LSDM/LCDM respectively). This character state distribution tends to exclude *Haestasaurus* from Saltasauridae or Titanosauria.
2. The prominent bulge on the posterolateral surface of the humerus (marking the attachment of the M. latissimus dorsi) is absent (character no. C226 in the LSDM/LCDM). This character state distribution tends to exclude *Haestasaurus* from Saltasauridae.
3. The attachment for the M. scapulohumeralis anterior on the humerus is weakly developed (character nos. 342 and C280 in the CSM and LSDM/LCDM respectively). This character state distribution tends to exclude *Haestasaurus* from Titanosauria (N.B. the derived state also occurs in *Giraffatitan*, but this is most parsimoniously interpreted as resulting from convergence).
4. The lateral and medial anterodistal processes of the humerus remain separated from each other by a notch (character no. C227 in the LSDM/LCDM). This character state distribution tends to exclude *Haestasaurus* from Titanosauria or *Chubutisaurus*+Titanosauria, depending on the precise topology under consideration.
5. Anconeal (= supracondylar) fossa of the humerus is relatively shallow (character no. C228 in the LSDM/LCDM). This character state distribution tends to exclude *Haestasaurus* from Titanosauriformes (see ‘RC2’ above for discussion of this character).
6. The distal articular surface of the humerus does not curve up onto the anterior face of the shaft (character nos. 257 and C229 in the CSM and LSDMLCDM respectively). This character state distribution tends to exclude *Haestasaurus* from Saltasauridae.
7. The distal articular surface of the humerus is flat transversely, rather than divided into two condyles by a shallow groove (character nos. 258 and C230 in the CSM and LSDM/LCDM respectively). This character state distribution tends to exclude *Haestasaurus* from Somphospondyli or Saltasauridae (but there are several instances of state reversal and convergence in this character).
8. The proximal end of the ulna is ‘V’-shaped in outline (character nos. 343 and C281 in the CSM and LSDM/LCDM respectively). This character state distribution tends to exclude *Haestasaurus* from Titanosauria.
9. The distal end of the ulna expands posteriorly (character no. C236 in the LSDM/LCDM). This character state distribution tends to exclude *Haestasaurus* from Titanosauriformes or a less inclusive clade (but there is considerable homoplasy, including the presence of the plesiomorphic state in taxa such as *Opisthocoelicaudia*).
10. The radius is slender (proximal transverse width:proximodistal length ratio is less than 0.3) (character no. C45 in the LSDM/LCDM). This character state distribution tends to exclude *Haestasaurus* from Saltasauridae.
11. The longitudinal ridge on the posterior surface of the radius is absent on the proximal half of the shaft (character no. C232 in the LSDM/LCDM). This character state distribution tends to exclude *Haestasaurus* from Titanosauria or a slightly more inclusive clade (but there is considerable homoplasy in this character).
12. The transverse width of the distal end of the radius is less than twice the width of the shaft at midlength (character nos. 266 and C47 in the CSM and LSDMLCDM respectively). This character state distribution tends to exclude *Haestasaurus* from Titanosauria or *Chubutisaurus*+Titanosauria (but this is highly homoplastic, with taxa such as *Apatosaurus*, *Camarasaurus* and *Giraffatitan* also possessing the derived state [see ‘Description’]).
13. Relatively little anteroposterior compression of the distal end of the radius (mediolateral:anteroposterior width ratio is less than 1.9) (character no. C48 in the LCDM). This character state distribution tends to exclude *Haestasaurus* from Saltasauridae (though note that *Opisthocoelicaudia* lacks the high ratio seen in other saltasaurids).
14. The distal radius possesses posterolateral and posteromedial condyles (character nos. 347 and C285 in the CSM and LSDM/LCDM respectively). This character state distribution tends to exclude *Haestasaurus* from Titanosauria (or possibly Titanosauriformes because the reversal that produces loss of the distal radial condyles in titanosaurs also occurs in taxa such as *Cedarosaurus*, *Lusotitan* and *Venenosaurus*). However, there is considerable homoplasy in this character, with the condyles occurring in some titanosauriforms such as *Giraffatitan* and *Rapetosaurus*.

In contrast, the following eight characters support inclusion of *Haestasaurus* within Titanosauriformes, or a less inclusive clade such as Somphospondyli, Titanosauria or Lithostrotia:

1. A robust humerus (i.e. a humeral RI value of 0.32 or higher for character no. 256 in the CSM, or a minimum midshaft width to humerus proximodistal length ratio of 0.18 or higher for C42 in the LSDM/LCDM). This character state distribution tends to support inclusion of *Haestasaurus* within Lithostrotia (although RI values above 0.32 occur convergently in a few other taxa such as *Suuwassea*).
2. A prominent olecranon process (character nos. 263 and C233 in the CSM and LSDM/LCDM respectively). This character state distribution tends to support inclusion of *Haestasaurus* within Somphospondyli or Titanosauria (depending on the optimisation of missing data). However, as noted in the ‘Description’, several non-titanosaurs also possess relatively large olecranon processes.
3. Robust ulna (character nos. 264 and C50 in the CSM and LSDM/LCDM respectively). This character state distribution tends to support inclusion of *Haestasaurus* within Saltasauridae (but with several convergences elsewhere among sauropods).
4. The proximal anteromedial process of the ulna has a strongly concave articular surface in anterior or posterior view (character no. C234 in the LSDM/LCDM). This character state distribution tends to include *Haestasaurus* within Titanosauriformes, especially Titanosauria.
5. The radius:humerus length ratio is greater than 0.6 (character no. C44 in the LSDM/LCDM). It appears that basal sauropods and eusauropods have values greater than 0.6, but this ratio drops below this value in many titanosauriforms. The presence of values greater than 0.6 in *Diamantinasaurus*, *Haestasaurus*, *Opisthocoelicaudia* and *Rapetosaurus* could therefore be interpreted as a derived reversal to the state seen in basal sauropods.
6. The proximal end of the radius is subtriangular in outline, tapering towards its medial process (character nos. 345 and C283 in the CSM and LSDM/LCDM respectively). This character state distribution tends to support inclusion of *Haestasaurus* within Titanosauriformes (although the derived state also occurs in a few taxa, such as *Aragosaurus* and *Lusotitan*, whose status as titanosauriforms is uncertain or contradicted by some phylogenetic analyses [18,111]).
7. Ridge- or flange-like projection on the medial margin of the radius for attachment of the M. biceps brachii and M. brachialis inferior (character nos. 346 and C284 in the CSM and LSDM/LCDM respectively). This character state distribution tends to support inclusion of *Haestasaurus* within Titanosauriformes (although the derived state is absent in some members of this clade, such as *Saltasaurus*, *Venenosaurus* and *Wintonotitan*).
8. The distal end of the radius is bevelled proximolaterally at 20° or more to the horizontal plane (character nos. 267 and C49 in the CSM and LSDM respectively). This character state distribution tends to support inclusion of *Haestasaurus* within Saltasauridae or a slightly more inclusive clade within Titanosauria (but with several convergent acquisitions elsewhere among sauropods, such as *Apatosaurus*). However, in LCDM MPTs, an angle of 25° or more unites Saltasauridae, to the exclusion of *Haestasaurus*.

Thus the evidence available from character mapping is equivocal, as is expected given the bimodal placement of *Haestasaurus* in the phylogenetic analyses presented above. Moreover, the fact that two separate data sets (CSM and LSDM) produce similar results when conventional parsimony analysis is applied, whereas a radically different topology is generated by the LCDM, suggests that the selection of different methodological approaches is key to the lack of consensus regarding the relationships of *Haestasaurus*. It could be argued that more weight should be given to the LCDM result because this data set avoids some of the problems associated with the discretisation of quantitative characters (e.g. see [132]). Moreover, Goloboff et al. [133] found that the application of implied weighting produced MPTs that are more stable over research time (i.e. topologies change less markedly as new taxa and characters are added) than those produced by conventional parsimony approaches. Despite these theoretical points in favour of the LCDM result, we are sceptical about the suggestion that *Haestasaurus* is a titanosaur. This is partly because the current LCDM MPTs contain a very unexpected topology in which a clade containing *Andesaurus*, *Chubutisaurus* and other taxa, is the sister-taxon to a cluster of traditional brachiosaurids (Fig 17). Such a result has not been found by any previous analysis, and the fact that this topology differs so markedly from the LCDM MPTs found by Mannion et al. [18] somewhat undermines the suggestion that the application of implied weighting produces more stable trees through research time. Finally, the majority of fore limb characters suggest that it is unlikely that *Haestasaurus* is a member of Titanosauria, and most of the few remaining characters support placement within Titanosauriformes rather than less inclusive clades such as Somphospondyli or Titanosauria. Here, therefore, we conclude that *Haestasaurus* is most plausibly interpreted as a non-titanosauriform macronarian, rather than a titanosauriform, somphospondylan or titanosaur, contrary to most recent studies [1,6,17,18,36,75–77]. If correct, then *Haestasaurus* convergently acquired certain features of the fore limb seen in derived titanosaurs. These features include increased robustness of the humerus and ulna, the enlarged olecranon, a concave profile to the anteromedial process of the ulna, and strong proximolateral bevelling of the distal end of the radius. Many of these derived character states were initially considered to be synapomorphies of the Titanosauria, or a less inclusive titanosaurian clade (e.g. [1,6,7,10]), but have subsequently been shown to be more widespread among Sauropoda [17–19]. Presumably, the repeated occurrence of these features in different regions of the sauropod tree reflects convergence in fore limb function, but at present the biomechanical and palaeoecological significance of this phenomenon is unclear.

## Sauropod Evolution in the Early Cretaceous

Jurassic sauropod faunas were dominated by various non-neosauropod eusauropod clades (e.g. mamenchisaurids, turiasaurians) and, in the Late Jurassic, the specialised flagellicaudatans [1,2]. At, or close to, the Jurassic-Cretaceous (J-K) boundary, sauropods underwent a major extinction, in which 60–80% of taxa disappeared [2,23]. Cretaceous sauropod faunas were rather different from those of the Jurassic, being dominated by narrow-tooth-crowned clades (notably rebbachisaurids and titanosaurs with dental slenderness indices [sensu 7] of 3.0 or higher) from the late Early Cretaceous onwards [2,25,134,135]. The earliest Cretaceous (Berriasian-Barremian) therefore represents an interesting ‘transitional’ period in which a few broad-toothed sauropods persisted (but dwindled to extinction) and titanosauriforms and rebbachisaurids radiated. *Haestasaurus* thus potentially represents one of the few non-titanosauriform macronarians that survived into the earliest Cretaceous.

Table 3 summarises those putative Early Cretaceous sauropods that have either been proposed to be non-titanosauriform macronarians, or potentially represent broad-toothed non-macronarians. There are 21 such taxa, but the phylogenetic relationships of most of these are uncertain. Many of the putative non-titanosauriform macronarians are more plausibly interpreted as titanosauriforms, somphospondylans or even titanosaurs. For example, *Xianshanosaurus* was identified as a basal neosauropod by Lü et al. [136], but this was not based on a phylogenetic analysis. The only formal cladistic analyses to examine the relationships of *Xianshanosaurus* are those of Mannion et al. [18] (and subsequent studies such as [80] and here, based on the LSDM and LCDM data sets), which include first-hand observations of this Asian taxon (PDM pers. obs. 2012). These analyses all agree that *Xianshanosaurus* is a titanosaur. When doubtful non-titanosauriform macronarians are excluded, there are only four genera that can be provisionally regarded as true basal macronarians: *Aragosaurus*, *Galveosaurus*, *Haestasaurus*, and the unnamed Dalton Wells sauropod. There are also four other broad-crowned (or at least potentially broad-crowned) sauropods, two of which are probably not macronarians (*Losillasaurus* and *Turiasaurus*) and two that have uncertain affinities (*Oplosaurus* and the unnamed Hell Canyon sauropod). However, it should be noted that the four Spanish taxa (*Aragosaurus*, *Galveosaurus*, *Losillasaurus* and *Turiasaurus*) might actually be Late Jurassic in age (Table 3): if so, then Early Cretaceous non-rebbachisaurid and non-titanosauriform sauropods are very rare indeed.

**Table 3:** Summary of putative Early Cretaceous non-titanosauriform macronarians and other broad-toothed sauropods, according to previous studies.

Taxon	Formation/Age/Country	Identification
* *Aragosaurus*	Villar del Arzobispo Formation (late Tithonian-middle Berriasian), Spain	Identified as a titanosauriform by Canudo et al. [152] and D’Emic [17], but these opinions were not based on inclusion of *Aragosaurus* into a phylogenetic analysis. Recovered as MN in all analyses to date (e.g. [18,80,109,111]; LSDM and LCDM here).
*Cedarosaurus*	Cedar Mountain Formation (Barremian-early Albian), USA	Recovered as MN by Royo-Torres [109, but most other analyses place this taxon as a brachiosaurid [1,17–19], LCDM here) or somphospondylan ([122], CSM here).
*Chubutisaurus*	Cerro Barcino Formation (Aptian-Cenomanian), Argentina	Recovered as a MN by Carballido et al. [15], but most other analyses place this taxon as a somphospondylan [14,17–19], CSM here) or at least a titanosauriform (LCDM here).
*Dongbeititan*	Yixian Formation (Barremian), China	Recovered as MN in the LCDM analysis of Mannion et al. [18], but this is not supported by their LSDM analysis or the LCDM result here.
*Euhelopus*	Mengyin Formation (Barremian-Aptian), China	Recovered as MN by Carballido et al. [15], Carballido and Sander [19] and CSM Here, but most other analyses place this taxon within Somphospondyli ([10,13,17,18], LSDM and LCDM here) or at least a titanosauriform [14]
* *Galveosaurus*	Villar del Arzobispo Formation (late Tithonian-middle Berriasian), Spain	Recovered as EU by Royo-Torres and Upchurch [110] in part, and Royo-Torres et al. [111], but most studies support an MN position ([15,18,19], CSM, LSDM and LCDM here).
* *Haestasaurus*	Hastings Beds Group (late Berriasian-Valanginian), UK	Regarded as a titanosauriform [17], somphospondylan [18,36], or even a titanosaur ([6,75], LCDM here), but here identified as a probable MN (see main text for details).
** *Jobaria*	Tiouaren Formation (Middle or Late Jurassic), Niger	Originally dated as Early Cretaceous by Sereno et al. [153], but more recently proposed as Middle Jurassic in age [145]
*Liubangosaurus*	Napai Formation (Aptian), China	Identified as EU by Mo et al. [154], as a macronarian (LSDM here), a somphospondylan ([18]: [LSDM), and a saltasaurid ([18]: LSDM with implied weights; LCDM] and LCDM here).
** *Losillasaurus*	Villar del Arzobispo Formation (late Tithonian-middle Berriasian), Spain	Identified as a diplodocoid by Casanovas et al. [155], but all subsequent analyses agree that this taxon is EU ([12,16,19,39,156], CSM here).
*/** *Oplosaurus*	Wessex Formation (Barremian), UK	Identified as either EU or MN [1,36].
*Sonorasaurus*	Turney Ranch Formation (late Albian-early Cenomanian), USA	Recovered as MN by Royo-Torres [109], but other analyses place this taxon as a titanosauriform ([17,18], LCDM here).
*Tangvayosaurus*	Grés Supérieurs Formation (Aptian-Albian), Laos	Recovered as MN by Royo-Torres [109], but all other analyses place this taxon in Somphospondyli ([17,18], LSDM and LCDM here).
*Tastavinsaurus*	Forcall and Xert Formations (early Aptian), Spain	Recovered as MN by [14,15,19] and the CSM here, but most studies place this taxon within Titanosauriformes ([17,18,157], LSDM and LCDM here).
** *Turiasaurus*	Villar del Arzobispo Formation (late Tithonian-middle Berriasian), Spain	Recovered as a titanosauriform by Mateus [158], but most studies have identified this taxon as EU ([19,109–111,159], CSM here).
*Unnamed Dalton Wells taxon	Cedar Mountain Formation (Barremian), USA	Initially identified as a titanosaur (e.g. [160]), but more recently interpreted as MN ([161], PU pers. obs. 2013).
*Unnamed Hell Canyon specimen	Lakota Formation (late Berriasian-Valanginian), USA	A *Camarasaurus*-like basal macronarian [162]
*Venenosaurus*	Cedar Mountain Formation (Barremian), USA	Recovered as MN by Royo-Torres [109] and Carballido et al. [14,15], but most other analyses place this taxon as a brachiosaurid ([17–19], LCDM here) or somphospondylan ([122,157], CSM here).
*Wintonotitan*	Winton Formation (Cenomanian-Turonian), Australia	Recovered as MN by Carballido et al. [14,15], but most other analyses place this taxon within Somphospondyli or Titanosauria (e.g. [1,18,19,80,163], CSM, LSDM and LCDM here). Poropat et al. [81] also presented additional character data that support inclusion of *Wintonotitan* within Somphospondyli.
*Xenoposeidon*	Ashdown Formation (late Berriasian-Valanginian), UK	Phylogenetic analysis by Taylor and Naish [37] placed this taxon within Neosauropoda, and Upchurch et al. [36] and Mannion et al. [18] tentatively identified it as MN. The extreme incompleteness of the holotype (and only) specimen means that the affinities of this taxon remain uncertain.
*Xianshanosaurus*	Mangchuan Formation (Cenomanian), China	Identified as a basal neosauropod by Lü et al. [136], but the phylogenetic analyses of Mannion et al. [18], and the LSDM/LCDM here, place this taxon within Somphospondyli or a more restricted clade within Titanosauria.

‘*’ mark those taxa that are accepted here to be probable non-titanosauriform macronarians;

‘**’ mark those taxa that are accepted here to be probable non-neosauropod eusauropods.

Formation and age data were obtained from *Fossilworks* (http://fossilworks.org/), *The Paleobiology Database* (http://paleobiodb.org/#/) (both accessed on 15^th^ August 2014), Upchurch et al. [36] and Mannion et al. [18]. Abbreviations: EU, non-neosauropod eusauropod; MN, non-titanosauriform macronarian.

Current evidence suggests that basal macronarians and other broad-crowned sauropods died out in Gondwana at the end of the Jurassic, and only persisted into the earliest Cretaceous in Europe and North America. This pattern might indicate some regional variations in the J-K boundary diversity decline among sauropods, perhaps reflecting differences in environmental conditions. However, the record of earliest Cretaceous sauropods has probably been strongly influenced by sedimentary rock availability and sampling. In particular, sauropod-bearing deposits of Berriasian-Valanginian age are rare globally, especially in Gondwanan continents. The *Paleobiology Database* (www.paleobiodb.org: accessed 15^th^ August 2014) and *Fossilworks* (www.fossilworks.org: accessed 15^th^ August 2014) list only 13 collections of sauropod body fossils from the Berriasian-Barremian of Gondwana: (1) the Bajada Colorada Formation (late Berriasian-Valanginian), Argentina, which has yielded the diplodocid *Leinkupal* [137]; (2) an unnamed unit (late Hauterivian-early Barremian), Croatia, which has produced the rebbachisaurid *Histriasaurus* and indeterminate titanosauriforms [138,139]; (3) an unnamed unit (Berriasian-Hauterivian), Lebanon, which has yielded two collections of titanosauriform teeth (possibly a brachiosaurid) [140]; (4) the Cabao Formation (Hauterivian-Barremian), Libya, which has yielded an incomplete sauropod tooth [141]; (5) the Irhazer Shale Formation (Berriasian-Valanginian), Niger, which has produced six collections of sauropod remains [142] (but see below); and (6) two collections from the Kirkwood Formation (Berriasian-Valanginian), South Africa, which include “*Algoasaurus*” and other indeterminate sauropods [143,144]. It could be argued that the existence of these Gondwanan deposits means that we do have opportunities to observe Southern Hemisphere basal macronarians and/or broad-crowned taxa from the Early Cretaceous if they were present. Note, however, that the quality and quantity of the specimens from five of these formations is low, and 10 of the localities are limited to only a small part of Gondwana (i.e. the northern part of the Afro-Arabian plate). Moreover, recent studies [145,146] have argued that the age of the Irhazer Formation of Niger is probably Middle Jurassic, rather than Early Cretaceous. Thus, sampling of Early Cretaceous sauropod-bearing deposits in Gondwana would need to improve substantially before we can conclude, with any confidence, that basal macronarians were genuinely absent.

Considerations of fossil record quality aside, we tentatively suggest that the J-K boundary extinction reduced the geographic range of broad-crowned sauropods by exterminating them in most regions apart from Europe and North America. Broad-crowned sauropods, especially non-titanosauriform macronarians, were still relatively diverse and abundant in Europe during the earliest Cretaceous (N.B. but this relative diversity strongly depends on the precise age of the four Spanish genera mentioned earlier). Their presence in the late Berriasian-Valanginian Lakota Formation and Barremian Dalton Wells Quarry implies the persistence of endemic North American lineages from the Late Jurassic into the earliest Cretaceous. Although poor sampling obscures the true geographic distribution of broad-crowned sauropods in the earliest Cretaceous, it seems likely that such taxa were restricted to Laurasia from approximately the Hauterivian onwards, and even here they declined in diversity and eventually disappeared by the end of the Early Cretaceous.

## Conclusion

The English sauropod “*Pelorosaurus*” *becklesii* is based on fore limb elements and a skin impression. This taxon can be diagnosed on the basis of five autapomorphies, and is distinct from *Pelorosaurus conybeari*; therefore the new generic name *Haestasaurus* is warranted. The phylogenetic relationships of *Haestasaurus* remain uncertain, although it can be regarded as a non-brachiosaurid macronarian based on several phylogenetic analyses. Moreover it is likely that *Haestasaurus* is a basal macronarian, perhaps closely related to *Janenschia*, *Tehuelchesaurus* and/or *Camarasaurus*. The evidence for any particular phylogenetic relationship is weak, mainly because of the incompleteness of the holotypic material of *Haestasaurus* and prevalent homoplasy in fore limb characters. Several new characters might make a useful contribution to future phylogenetic analyses, and here they generally reinforce the view that *Haestasaurus* is basal within Macronaria. If *Haestasaurus* is a basal macronarian, then this adds to the evidence that such taxa (also including *Aragosaurus*, *Galveosaurus* and the unnamed Dalton Wells sauropod) survived the Jurassic-Cretaceous boundary decline of diplodocid and broad-toothed sauropods and did not finally die out until some point in the late Early Cretaceous. However, it is not clear whether the occurrence of several Early Cretaceous non-titanosauriform macronarians solely in Europe and North America indicates differential regional patterns in extinction/survival across the J-K boundary, or merely reflects poor sampling of earliest Cretaceous deposits elsewhere.

## Supporting Information

S1 File Character scores for *Haestasaurus* and for six new characters (NC1-6). (DOCX)

S2 File The CSM data matrix formatted for TNT. (TXT)

S3 File The LSDM data matrix formatted for TNT. (TXT)

S4 File The LCDM data matrix formatted for TNT. (TXT)
