# Supplementary material for: The Anatomy and Phylogenetic Relationships of “Pelorosaurus“ becklesii (Neosauropoda, Macronaria) from the Early Cretaceous of England
Source: PLoS One. 2015 Jun 3;10(6):e0125819. doi: 10.1371/journal.pone.0125819 (PMC4454574; doi:10.1371/journal.pone.0125819)
Supplement: S1 File — (DOCX) [file pone.0125819.s001.docx]

Supporting Information File S1

The Anatomy and Phylogenetic Relationships of “*Pelorosaurus*“ *becklesii* (Neosauropoda, Macronaria) from the Early Cretaceous of England

Paul Upchurch, Philip D. Mannion. Michael P. Taylor

This file contains summaries of the character scores assigned to *Haestasaurus* (Section 1) and character scores for the six new characters NC1-6 (Section 2). Tables A-C are included in this file.

Section 1. Character scores for *Haestasaurus*

1.1. Carballido and Sander [19] - CSM

The humerus, ulna and radius can be scored for 15 out of 341 characters in this data set. Thus all characters for *Haestasaurus* are assigned ‘?’ except for the following:

State 0 = characters 254, 257, 259, 263, 266,

State 1 = characters 253, 255, 258, 260-262, 264, 265, 267,

State 2 = character 256

For ease of reference, the 15 characters scored for the CSM are listed below:

253. Humeral deltopectoral attachment, development: prominent (0); reduced to a low crest or ridge (1) ([10]: character no. 160).

254. Humeral deltopectoral crest, shape: relatively narrow throughout length (0); markedly expanded distally (1) ([10]: character no. 161).

255. Humeral midshaft cross-section, shape: circular (0); elliptical (1) ([39]: character 170).

256. Humerus, RI [sensu 31]: Gracile (less than 0,27) (0); medium (0,28-0,32) (1); Robust (more than 0,33) (2) [123].

257. Humeral distal condyles, articular surface shape: restricted to distal portion of humerus (0); exposed on anterior portion of humeral shaft (1) ([10]: character no. 163).

258. Humeral distal condyle, shape: divided (0); flat (1) ([10]: character no. 164).

259. Humeral, lateral margin: medially deflected (0); almost straight until the half length or even more (1) [123].

260. Humeral proximolateral corner, shape: rounded, the dorsal surface is well convex (0); pronounced / square, the dorsal surface low, almost flat (1) ([10]: character no. 159).

261. Ulnar proximal condyle, shape: subtriangular (0); triradiate, with deep radial fossa (1) ([10]: character no. 165).

262. Ulnar proximal condylar processes, relative lengths: subequal (0); unequal, anterior arm longer (1) ([10]: character no. 166).

263. Ulnar olecranon process, development: prominent, projecting above proximal articulation (0); rudimentary, level with proximal articulation (1) ([10]: character no. 167).

264. Ulna, length-to-proximal breadth ratio: gracile (0); stout (1) ([10]: character no. 168).

265. Radial distal condyle, shape: round (0); subrectangular, flattened posteriorly and articulating in front of ulna (1) ([10]: character no. 169).

266. Radius, distal breadth: slightly larger than midshaft breadth (0); approximately twice midshaft breadth (1) ([10]: character no.170).

267. Radius, distal condyle orientation: perpendicular to long axis of shaft (0); bevelled approximately 20º proximolaterally relative to long axis of shaft (1) ([10]: character no. 171).

1.2. Mannion et al. [18] - LSDM and LCDM

Mannion et al. [18] presented two matrices (see main text). The LSDM data set is entirely composed of discrete or discretised quantitative characters, whereas the LCDM data set treated the quantitative characters as continuous data. Both data sets placed the quantitative characters as the first 74 characters listed in the matrix. As a result, humeral, ulnar and radial characters are C41-51 (quantitative) and C223-236 (discrete). For characters C41-51 below, state scores of 0, 1, etc. represent the discretised character scores used in the LSDM, whereas values expressed as ratios represent the continuous data used directly in TNT when analysing the LCDM.

LSDM

State 0 = characters C41, C42, C44, C45, C47, C48, C51.

State 1 = characters C43, C46, C49, C50.

LCDM

C41 = 0.447; C42 = 0.189; C43 = 1.449; C44 = 0.674, C45 = slightly greater than 0.248, C46 = 0.89-0.92, C47 = 1.852, C48 = 1.527, C49 = 21 degrees, C50 = 0.442, C51 = 1.29.

Table A. Summary of the ratios used for C51 in the LCDM (all taxa not listed blow were scored with state '?' by Mannion et al [18] and in the current study). This table shows the ratios used by Mannion et al. [18], and the revised values resulting from the change to the way the lengths of the proximal ulnar processes are measured (see ‘RC3” and Fig. 13 In the main text).

| Taxon | Mannion et al. [18] | New value (RC3) |
| --- | --- | --- |
| *Omeisaurus* | 1.28 | 1.0 |
| *Mamenchisaurus* | 1.04 | 1.10 |
| *Camarasaurus* | 1.34 | 1.34 |
| *Nigersaurus* | 1.18 | 1.40 |
| *Apatosaurus* | 1.03 | 1.0 |
| *Diplodocus* | 1.05 | 1.23 |
| *Alamosaurus* | 1.32 | 1.23 |
| *Angolatitan* | 1.05 | 1.50 |
| *Aragosaurus* | 1.11 | 1.11 |
| *Cedarosaurus* | 2.88 | 2.56 |
| “Cloverly titanosauriform” | 2.15 | 1.86 |
| *Diamantinasaurus* | 1.0 | 1.43 |
| *Europasaurus* | 1.05 | 1.15 |
| *Giraffatitan* | 1.41 | 1.43 |
| *Lapparentosaurus* | 1.12 | 1.12 |
| *Malawisaurus* | 1.05 | 1.42 |
| *Opisthocoelicaudia* | 0.86 | 1.64 |
| *Paluxysaurus* | 1.49 | 1.2 |
| *Haestasaurus* | 1.16 | 1.29 |
| *Rapetosaurus* | 1.14 | 1.63 |
| *Saltasaurus* | ? | 1.47 |
| *Tehuelchesaurus* | 1.25 | 1.50 |
| *Venenosaurus* | 2.028 | 2.0 |

LSDM and LCDM (discrete characters common to both data sets)

State 0 = characters C224-C232, C235, C236.

State 1 = characters C223, C233, C234.

For ease of reference, the 25 characters scored for the LSDM an LCDM are listed below:

C41. Humerus, maximum mediolateral width of proximal end divided by proximodistal length: 0.4 or greater (0); less than 0.4 (1) [18].

C42. Humerus, minimum mediolateral width divided by proximodistal length: 0.15 or greater (0); less than 0.15 (1) ([11], modified and polarity reversed by [18]).

C43. Humerus shaft eccentricity, mediolateral to anteroposterior width ratio at midshaft: greater than 1.5 (usually close to 1.8) (0); 1.5 or lower (usually close to 1.3) (1) ([10,39], polarity reversed by [18]).

C44. Radius to humerus proximodistal length ratio: 0.65 or greater (0); less than 0.65 (1) ([164], modified by [18]).

C45. Radius, maximum diameter of the proximal end divided by proximodistal length: less than 0.3 (0); 0.3 or greater (1) [1,6,7,64].

C46. Radius, mediolateral width of proximal to distal end ratio: 1.0 or greater (0); less than 1.0 (1) ([11], modified, quantified, and polarity reversed by [18]; note that in taxa with a twisted radius, the dimension of the long axis of the distal end is used).

C47. Radius, distal end mediolateral width to midshaft mediolateral width ratio: less than 2.0 (0); 2.0 or greater (1) ([10,122], modified by [18]; note that in taxa with a twisted radius, the dimension of the long axis of the distal end is used).

C48. Radius, distal end mediolateral to anteroposterior width ratio: 1.5 or greater (0); less than 1.5 (1) ([9], quantified and polarity reversed by [18]).

C49. Radius, distal condyle orientation: perpendicular or bevelled less than 20° to long axis of shaft (0); bevelled at least 20° to long axis of shaft (1) ([10,114], modified by [18]; note that in most taxa only the lateral half of the distal end is bevelled, but this is used as the measurement in those instances).

C50. Ulna, ratio of maximum mediolateral width of proximal end to ulna length: gracile, ratio is less than 0.4 (0); stout, ratio is 0.4 or greater (1) ([10,11], modified by [18]).

C51. Ulna, ratio of maximum mediolateral width of proximal end (equivalent to anteromedial arm) to maximum anteroposterior width of proximal end (equivalent to anterolateral arm): less than 2.0 (0); 2.0 or greater (1) ([10], modified and quantified by [18]). N.B. see the revised state definitions presented in RC3 in the main text (see Fig. 13).

C223. Humeral proximolateral corner, shape: rounded, surfaces merge smoothly into each other to produce a transversely rounded proximal end, with the proximal-most point of the lateral margin at a lower level than the remaining lateral half of the proximal surface (0); square, surfaces meet each other at an abrupt angle to produce a ‘squared’ proximal end in anterior view, with the proximal-most point of the lateral margin level with the remaining lateral half of the proximal surface (1) [1,10,12,85], modified by [18]).

C224. Humerus, shape of lateral margin of diaphysis (approximately the middle third of the humerus) in anterior view: concave (0); straight (1) ([11], modified and polarity reversed by [18]).

C225. Humeral deltopectoral crest: restricted to lateral edge of humerus and projects anteriorly or anterolaterally (0); extends medially across the anterior face of the humerus (1) ([1,10], modified by [18]).

C226. Humerus, strong bulge or tuberosity (site for M. latissimus dorsi) close to the lateral margin of the posterior surface, at approximately the level of the distal tip of the deltopectoral crest: absent (0); present (1) [17,27,90].

C227. Humerus, anterior surface of distal lateral condyle: divided by a notch, forming two ridges (0); undivided (1) [17].

C228. Humerus, distal-most part of the posterior surface (supracondylar fossa) is: flat or shallowly concave (0); deeply concave between prominent lateral and medial vertical condylar ridges (1) [1]. N.B. see the revisions to the state definitions in ‘RC2’ in the main text.

C229. Humeral distal condyles, articular surface: flat anteroposteriorly and restricted to distal portion of humerus (0); anteroposteriorly convex so that it curves up onto the anterior and posterior faces of the humerus (1) [1,10].

C230. Humeral distal articular surface, condyles: undivided (0); divided (1) ([10], modified and polarity reversed by [18]).

C231. Radius, strong twist in axis, such that the long axes of the proximal and distal ends are not orientated in the same plane: absent (0); present (1) [18].

C232. Radius, well-developed interosseous ridge that extends along most of the radius length (at least along the distal two-thirds): absent (0); present (1) ([11], modified by [18]).

C233. Ulnar olecranon process, development: absent or only rudimentary, i.e. projecting just above the proximal articulation (0); prominent, projecting well above proximal articulation (1) ([9,64], polarity reversed by [18]).

C234. Ulna, articular surface of anteromedial process is: flat (0); concave along its length (1) [6,7].

C235. Ulna, orientation of anteromedial process: flat or sloping downwards less than 40° (0); sloping downwards at an angle of at least 40° to the horizontal (1) [18].

C236. Ulna, distal end: prominently expanded posteriorly (0); unexpanded (1) [17].

Section 2. Scores for new characters (NC1-6)

Character scores for all taxa in the CSM, LSDM and LCDM for characters NC1-6 (see main text for details).

2.1. CSM

Table B. NC1-6 character scores for the CSM.

|  | NC1  (342) | NC2  (343) | NC3  (344) | NC4  (345) | NC5  (346) | NC6  (347) |
| --- | --- | --- | --- | --- | --- | --- |
| *Plateosaurus_engelhardti* | 0 | 0 | 0 | 0 | 0 | 0 |
| *Chinshakiangosaurus*  *chunghoensis* | ? | ? | ? | ? | ? | ? |
| *Mussaurus_patagonicus* | 0 | 0 | 0 | 0 | 0 | 0 |
| *Antetonitrus_ingenipes* | 0 | 0 | 1 | 0 | 0 | 0 |
| *Lessemsaurus_sauropoides* | 0 | 0 | ? | ? | ? | ? |
| *Gongxianosaurus_shibeiensis* | ? | ? | ? | ? | ? | ? |
| *Amygdalodon_patagonicus* | ? | ? | ? | ? | ? | ? |
| *Isanosaurus_attavipachi* | ? | ? | ? | ? | ? | ? |
| *Vulcanodon_karibaensis* | 0 | ? | ? | ? | 0 | 0 |
| *Tazoudasaurus_naimi* | 0 | 0 | 1 | ? | 0 | 0 |
| *Shunosaurus_lii* | 0 | 0 | ? | ? | ? | ? |
| *Barapasaurus_tagorei* | 0 | 0 | 0 | 0 | 0 | 0 |
| *Cetiosaurus_oxoniensis* | 0 | 0 | ? | ? | ? | ? |
| *Patagosaurus_fariasi* | 0 | 0 | 0 | 0 | 0 | 0 |
| *Omeisaurus* | 0 | 0 | 0 | 0 | ? | 0 |
| *Mamenchisaurus* | 0 | 0 | 0 | 0 | 0 | 0 |
| *Turiasaurus_riodevensis* | 0 | ? | 0 | 1 | 0 | 1 |
| *Losillasaurus_giganteus* | ? | ? | 0 | ? | ? | ? |
| *Jobaria_tiguidensis* | 1 | 0 | 0 | 1 | 0 | 0 |
| *Haplocanthosaurus_priscus* | ? | ? | ? | ? | ? | ? |
| *Camarasaurus* | 0 | 0 | 0 | 0 | ? | 1 |
| *Bellusaurus_sui* | 0 | 0 | 0 | 0 | 0 | 1 |
| *Galvesaurus_herreroi* | 0 | ? | ? | ? | ? | ? |
| *Tehuelchesaurus_benitezii* | 0 | 0 | 0 | 0 | 0 | 1 |
| *Tastavinsaurus_sanzi* | ? | ? | ? | 1 | ? | 1 |
| *Euhelopus_zdanskyi* | 0 | ? | ? | ? | ? | ? |
| *Brachiosaurus_altithorax* | 0 | ? | ? | ? | ? | ? |
| *Giraffatitan_brancai* | 1 | 0 | 0 | 1 | 1 | 1 |
| *Paluxysaurus_jonesi* | 0 | 0 | 0 | 1 | ? | 1 |
| *Venenosaurus_dicrocei* | ? | 0 | 0 | 1 | 0 | 0 |
| *Cedarosaurus* | 0 | 0 | ? | 1 | ? | 0 |
| *Erketu_ellisoni* | ? | ? | ? | ? | ? | ? |
| *Chubutisaurus_insignis* | 0 | ? | ? | ? | ? | ? |
| *Tendaguria_tanzaniensis* | ? | ? | ? | ? | ? | ? |
| *Ligabuesaururs_lenzai* | 0 | ? | ? | ? | ? | ? |
| *Phuwiangosaurus_sirindhornae* | 0 | ? | ? | 1 | ? | ? |
| *Andesaurus_delgadoi* | ? | ? | ? | ? | ? | ? |
| *Mendozasaurus_neguyelap* | ? | ? | ? | ? | ? | ? |
| *Malarguesaurus_florenciae* | ? | ? | ? | ? | ? | ? |
| *Argentinosaurus_hunculensis* | ? | ? | ? | ? | ? | ? |
| *Epachthosaurus_sciuttoi* | 1 | 0 | 1 | 1 | 1 | 1 |
| *Malawisaurus_dixeyi* | 1 | 1 | 0 | 1 | ? | 0 |
| *Nemegtosaurus_mongoliensis* | ? | ? | ? | ? | ? | ? |
| *Rapetosaurus_krausei* | 0 | 1 | 0 | 1 | 1 | 1 |
| *Isisaurus_colberti* | 1 | 1 | ? | ? | ? | ? |
| *Tapuiasaurus_macedoi* | ? | ? | ? | 1 | ? | ? |
| *Trigonosaurus_pricei* | ? | ? | ? | ? | ? | ? |
| *Alamosaurus_sanjuanensis* | 1 | 1 | 0 | ? | ? | ? |
| *Opisthocoelicaudia_skarzynskii* | 1 | 1 | 0 | 0 | 1 | 0 |
| *Neuquensaurus_australis* | 1 | 1 | 1 | 1 | 0 | 0 |
| *Saltasaurus_loricatus* | 1 | 1 | 1 | 1 | 0 | 0 |
| *Amazonsaurus_maranhensis* | ? | ? | ? | ? | ? | ? |
| *Zapalasaurus_bonapartei* | ? | ? | ? | ? | ? | ? |
| *Histriasaurus_bocardeli* | ? | ? | ? | ? | ? | ? |
| *Comahuesaurus_windhanseni* | 0 | ? | ? | ? | ? | ? |
| *Rayososaurus_agrioensis* | ? | ? | ? | ? | ? | ? |
| *Rebbachisaurus_garasbae* | 0 | ? | ? | ? | ? | ? |
| *Cathartesaura_anaerobica* | ? | ? | ? | ? | ? | ? |
| *Limaysaurus_tessonei* | 0 | 0 | 0 | ? | ? | 1 |
| *Demandasaurus_darwini* | ? | ? | ? | ? | ? | ? |
| *Nigersaurus_taqueti* | 0 | 0 | 0 | 0 | 0 | 1 |
| *Suuwassea_emiliae* | 0 | ? | ? | ? | ? | ? |
| *Amargasaurus_cazaui* | 0 | 0 | 0 | 1 | 0 | 1 |
| *Dicraeosaurus_hansemanni* | 0 | 0 | 0 | ? | ? | ? |
| *Brachytrachelopan_messai* | ? | ? | ? | ? | ? | ? |
| *Apatosaurus* | 0 | 0 | 0 | 0 | 0 | 1 |
| *Diplodocus* | 0 | 0 | 0 | 0 | ? | ? |
| *Barosaurus_lentus* | 0 | ? | ? | ? | ? | ? |
| *Europasaurus_holgeri* | 0 | 0 | 0 | ? | ? | ? |
| *Diamantinasaurus matildae* | 0 | 1 | 1 | 1 | 1 | 0 |
| *Wintonotitan_wattsi* | ? | 1 | 0 | 1 | 0 | 0 |
| *Haestasaurus becklesii* | 0 | 0 | 0 | 1 | 1 | 1 |

2.2. LSDM/LCDM

Table C. NC1-6 character scores for the LSDM and LCDM.

|  | NC1  (C280) | NC2  (C281) | NC3  (C282) | NC4  (C283) | NC5  (C284) | NC6  (C285) |  |
| --- | --- | --- | --- | --- | --- | --- | --- |
| *Shunosaurus* | 0 | 0 | ? | ? | ? | ? |  |
| *Omeisaurus* | 0 | 0 | 0 | 0 | ? | 0 |  |
| *Mamenchisaurus* | 0 | 0 | 0 | 0 | 0 | 0 |  |
| *Camarasaurus* | 0 | 0 | 0 | 0 | ? | 1 |  |
| *Nigersaurus* | 0 | 0 | 0 | 0 | 0 | 1 |  |
| *Apatosaurus* | 0 | 1 | 0 | 0 | 0 | 1 |  |
| *Diplodocus* | 0 | 0 | 0 | 0 | ? | ? |  |
| *Abydosaurus* | ? | ? | ? | ? | ? | ? |  |
| *Alamosaurus* | 1 | 1 | 0 | ? | ? | ? |  |
| *Andesaurus* | ? | ? | ? | ? | ? | ? |  |
| *Angolatitan* | 1 | 1 | 0 | 1 | ? | 1 |  |
| *Aragosaurus* | 0 | 0 | 0 | 1 | 0 | 0 |  |
| *Astrophocaudia* | ? | ? | ? | ? | ? | ? |  |
| *Atlasaurus* | ? | ? | ? | ? | ? | ? |  |
| *Australodocus* | ? | ? | ? | ? | ? | ? |  |
| *Baotianmansaurus* | ? | ? | ? | ? | ? | ? |  |
| *Brachiosaurus* | 0 | ? | ? | ? | ? | ? |  |
| *Brontomerus* | ? | ? | ? | ? | ? | ? |  |
| *Cedarosaurus* | 0 | 0 | ? | 1 | ? | 0 |  |
| *Chubutisaurus* | 0 | ? | ? | ? | ? | ? |  |
| *"Cloverly titanosauriform"* | ? | 0 | 0 | ? | ? | ? |  |
| *Daxiatitan* | ? | ? | ? | ? | ? | ? |  |
| *Diamantinasaurus* | 0 | 1 | 1 | 1 | 1 | 0 |  |
| *Dongbeititan* | ? | ? | ? | ? | ? | ? |  |
| *Dongyangosaurus* | ? | ? | ? | ? | ? | ? |  |
| *Erketu* | ? | ? | ? | ? | ? | ? |  |
| *Euhelopus* | 0 | ? | ? | ? | ? | ? |  |
| *Europasaurus* | 0 | 0 | 0 | ? | ? | ? |  |
| *“French Bothriospondylus”* | 0 | ? | ? | ? | ? | ? |  |
| *Fukuititan* | 0 | ? | ? | ? | ? | ? |  |
| *Fusuisaurus* | ? | ? | ? | ? | ? | ? |  |
| *Galveosaurus* | 0 | ? | ? | ? | ? | ? |  |
| *Giraffatitan* | 1 | 0 | 0 | 1 | 1 | 1 |  |
| *Gobititan* | ? | ? | ? | ? | ? | ? |  |
| *HMN MB.R.2091.1-30* | ? | ? | ? | ? | ? | ? |  |
| *Huanghetitan* | ? | ? | ? | ? | ? | ? |  |
| *"Huanghetitan" ruyangensis* | ? | ? | ? | ? | ? | ? |  |
| *Janenschia* | 0 | 0 | 0 | 1 | 0 | 1 |  |
| *Jiangshanosaurus* | ? | ? | ? | ? | ? | ? |  |
| *Lapparentosaurus* | 0 | 0 | 0 | 0 | 0 | 0 |  |
| *Ligabuesaurus* | 0 | ? | ? | ? | ? | ? |  |
| *Liubangosaurus* | ? | ? | ? | ? | ? | ? |  |
| *Lusotitan* | 0 | 0 | ? | 1 | 0 | 0 |  |
| *Malarguesaurus* | ? | ? | ? | ? | ? | ? |  |
| *Malawisaurus* | 1 | 1 | 0 | 1 | ? | 0 |  |
| *Mongolosaurus* | ? | ? | ? | ? | ? | ? |  |
| *Opisthocoelicaudia* | 1 | 1 | 0 | 0 | 1 | 0 |  |
| *Paluxysaurus* | 0 | 0 | 0 | 1 | ? | 1 |  |
| *"Pelorosaurus" becklesii* | 0 | 0 | 0 | 1 | 1 | 1 |  |
| *Phuwiangosaurus* | 0 | ? | ? | 1 | ? | ? |  |
| *Qiaowanlong* | ? | ? | ? | ? | ? | ? |  |
| *Rapetosaurus* | 0 | 1 | 0 | 1 | 1 | 1 |  |
| *Ruyangosaurus* | ? | ? | ? | ? | ? | ? |  |
| *Saltasaurus* | 1 | 1 | 1 | 1 | 0 | 0 |  |
| *Sauroposeidon* | ? | ? | ? | ? | ? | ? |  |
| *Sonorasaurus* | ? | ? | ? | ? | ? | ? |  |
| *Tangvayosaurus* | ? | ? | ? | ? | ? | ? |  |
| *Tastavinsaurus* | ? | ? | ? | 1 | ? | 1 |  |
| *Tehuelchesaurus* | 0 | 0 | 0 | 0 | 0 | 1 |  |
| *Tendaguria* | ? | ? | ? | ? | ? | ? |  |
| *Venenosaurus* | ? | 0 | 0 | 1 | 0 | 0 |  |
| *Wintonotitan* | ? | 1 | 0 | 1 | 0 | 0 |  |
| *Xianshanosaurus* | ? | ? | ? | ? | ? | ? |  |

References 1–159 are listed in the main paper.

164. Yates AM, Kitching J. The earliest known sauropod dinosaur and the first steps towards sauropod locomotion. Proc Roy Soc London, Ser B. 2009;270: 1753–1758.
